# Age-standardized mortality, disability-adjusted life-years and healthy life expectancy in different cultural regions of Guangdong, China: a population-based study of 2005–2015

**DOI:** 10.1186/s12889-020-8420-7

**Published:** 2020-06-05

**Authors:** Xue-yan Zheng, Xiao-jun Xu, Yi-yang Liu, Yan-jun Xu, Si-xing Pan, Xin-ying Zeng, Qian Yi, Ni Xiao, Li-feng Lin

**Affiliations:** 1 Institute of Non-Communicable Disease Control and Prevention, Guangdong Provincial Center for Disease Control And Prevention, 160 Qunxian Road, Panyu District, Guangzhou, Guangdong China; 2 Department of Epidemiology and Biostatistics, School of Public Health, Guangdong Pharmaceutical University, Guangzhou, China; 3 National Center for Chronic and Non-Communicable Disease Control and Prevention, Chinese Center for Disease Control and Prevention, Beijing, China

**Keywords:** Burden of disease, Mortality, Disability-adjusted life-years, Healthy life expectancy, Cultural regions, Guangdong

## Abstract

**Background:**

Guangdong province is dominated by three cultural regions: Canton, Hakka and Hoklo. However, little is known about the disease burden within these regions, particularly because different population,environmental and socioeconomic risk factors might cause different patterns of mortality, disability-adjusted life-years (DALY), life expectancy and healthy life expectancy (HALE). We aimed to compare the patterns of disease burden in Canton, Hakka and Hoklo regions between 2005 and 2015.

**Method:**

We calculated the mortality, YLL, YLD for 116 diseases for different cultural regions between 2005 and 2015. We calculated the DALYs for 116 causes as the sum of YLLs and YLDs. We estimated the life expectancy and HALE by using sex-specific mortality rates and YLDs for the three cultural regions.

**Results:**

With a respective reduction of 22.3, 15.8 and 17.8% in 2015 compared with 2005, the age-standardized DALY rates in 2015 was 19,988.0, 14,396.5 and 20,436.6 in Hakka, Canton and Hoklo region. Canton region had a significantly lower mortality and DALYs in most diseases, followed by Hoklo and Hakka regions. The life expectancy and HALE at birth were highest in Canton region in both 2005 and 2015, than in Hoklo and Hakka region.

**Conclusions:**

Our findings call for improved public health care via the refinement of policy and effective measures for disease prevention. Understanding the environmental and culture-related risk factors of diseases in Hoklo and Hakka regions may help inform public health sectors to reduce the disease burden and the between-region inequality.

## Background

Guangdong is one of the leading developed provinces, with 108 million permanent residents that account for 8% of the population in China. Compared with other provinces, Guangdong has 56 ethnic populations, rendering it the most ethnically diverse province in China. For decades, the Canton, Hakka and Hoklo clans constitute the major Han ethnicity and blend with other ethnic populations within Guangdong province, shaping their own culture in each region. Between 2005 and 2015, Canton, Hakka and Hoklo cultural regions have a respective population ranging from 20 to 26 million, from 10 to 13 million, and from 12 to 13 million, accounting for nearly half of the population in Guangdong province. Currently, these cultural regions have different levels of social and economic development, ordered by Canton, Hoklo and Hakka regions. In addition, each region has kept their own living and cultural habits (e.g., drinking boiled Kongfu tea that led to the high prevalence of esophageal cancer in Hoklo region). The population, environmental and socioeconomic differences among the three cultural regions might have led to different burden of disease.

Measurement of the changes in population health across different geographic regions is informative for identifying the priority for resource allocation to research, policy development and decision making. In the 2016 Global Burden of Diseases study [1], two widely used summary measures have been applied to monitor these changes in the population health: disability-adjusted life-years (DALYs) and healthy life expectancy (HALE). The DALY measures the health loss due to both fatal and non-fatal disease burden, by summing the years-of-life-lost (YLLs) due to premature mortality and years-of-life lived with disability (YLDs) [2–4]. These metrics allow for a direct comparison between different diseases and causes of injury. Conversely, HALE provides with a single summary measure of population health across all causes by weighting the years lived with functional health loss before death [5–8].

Although health challenges during the recent three decades have been documented in China, little is known about the regional differences at provincial level, regarding different population, environmental and socioeconomic risk factors. To our knowledge, this is the first study of the regional-level burden of disease in Guangdong, China. We took advantage of the comprehensive demographic and epidemiological data sets that were assembled, including the census, demographic surveys, the Disease Surveillance Points system, the Chinese Center for Disease Control and Prevention Cause of Death Reporting System, and under-reporting of death survey. To more comprehensively characterize the data, we applied the China-specific garbage code redistribution, incompleteness and socioeconomic variables estimation.

Herein, we examined the age-standardized mortality and DALY rates for 116 causes of death by stratification of the time and sex in Canton, Hakka and Hoklo regions within Guangdong province between 2005 and 2015. Furthermore, we analyzed the time trends to explore the cultural region-related patterns of changes, and examined the cultural region-specific life expectancy based on a comprehensively updated database.

## Methods

**Fig. 1:**
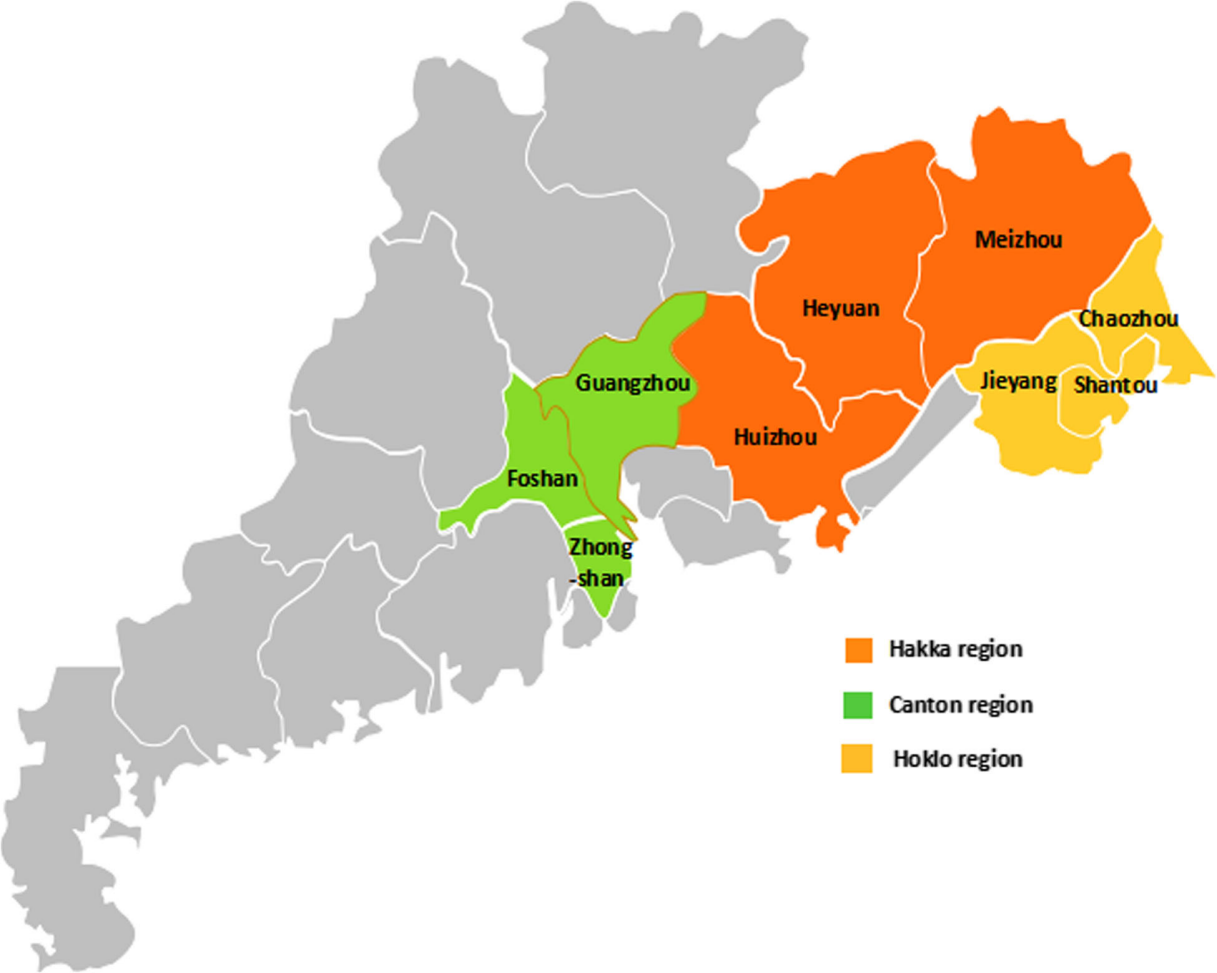
A map of three cultural regions in Guangdong Province

We adopted the method established by the Global Burden of Disease study, previously applied by the WHO and IHME [9–11]. Briefly, we focused on 116 disease cause-specific mortality in Canton (Guangzhou, Foshan and Zhongshan cities), Hakka (Meizhou, Huizhou and Heyuan cities) and Hoklo (Shantou, Chaozhou and Jieyang cities) regions (Fig. 1). Data sources were analyzed for county-level mortality because of the changes in administrative units. Further calculation of the cultural-region mortality was conducted based on the county level.

### Data sources

1. Death data source for estimation of the all-cause and specific disease mortality, DALY, life expectancy and HALE: the main data source for mortality estimates was derived from the Disease Surveillance Points, the Chinese Center for Disease Control and Prevention Cause of Death Reporting System and under-reporting of death survey at county level between 2005 and 2015 [12, 13]. Death information was mandatory in the Disease Surveillance Points, the Chinese Center for Disease Control and Prevention Cause of Death Reporting System in China. However, owing to the incompleteness of the death rate, under-reporting of death survey at county level was conducted between 2005 and 2015. Briefly, the county with an averaged level of mortality rate, population size and socioeconomic levels was selected to represent the city that the county belonged to. The death cases were surveyed through multiple routine measures, including the records from the residential committee, funeral home and police stations. To ascertain the degree of under-reporting, the information of death cases was further compared with the registered information from Chinese Center for Disease Control and Prevention Cause of Death Reporting System.
2. Covariate data source to estimate the mortality of children aged under 5 years (under-five mortality, U5MR) and adults: covariates including the duration of education, gross domestic product (GDP), and the rate of urbanization, were derived from the population census and National statistical yearbook between 2005 and 2015. Regarding the irregular fluctuations, the outlier and deletion of the covariates at county levels, different models were employed for provision of the more appropriate estimation. See Online Supplement for details.
3. Population data source to calculate the age-standardized mortality with the direct standardization method, adjusting for the population structure: the population at county level was derived from census from 2000 and 2010 using the Leslie model [14].

### Regional mortality estimation

The methods we used for regional all-cause mortality estimation were:

- STEP 1: The rate of incompleteness was calculated based on under-reporting of the death survey. Complete age- and sex- all-cause mortality at county level was adjusted with the incompleteness through the formula of all-cause mortality/ (1-incompleteness) (see Online Supplement);
- STEP 2: Under-five mortality and 15–59-year adult mortality were calculated, based on the World Health Organization’s life tables [15];
- STEP 3: Derivation of all-cause mortality and 95% confidence interval at county level was conducted, with the mixed-effect model that included socioeconomic variables of the lagged distributed income, the duration of education, the rate of urbanization, year, under-five mortality and 15–59-year adult mortality [15–19]. The quality of all-cause mortality adjusted with the completeness was deemed eligible for the cultural region calculation when falling within the 95% confidence interval, and ineligible when falling outside of the 95% confidence interval. Ineligible all-cause mortality at county level would be replaced by the estimation of mixed-effect model;
- STEP 4: Qualified mortality rates at the county level were included in a spatiotemporal locally weighted smooth regression (S-T LOESS) model, which was applied to estimate the sex-specific under-five mortality and 15–59-year adult mortality;
- STEP 5: Estimation of age-specific mortality at county level was applied with the model life tables on under-five mortality and 15–59-year adult mortality; and was re-scaled to match the 2015 GBD [8] provincial-level death numbers. The culture-region-level age-specific mortality and city-level estimates were obtained from the re-scaled county-level mortality.

### Cause-specific mortality estimation

We identified and re-distributed the ICD codes not assignable to the underlying causes of death, those with intermediate causes of death, or those that lacked specificity (see details of garbage code re-distribution in Online Supplement).

Crude mortality greater than 3‰ at city levels was included in cause-specific mortality estimation, or re-calculated with S-T LOESS model that included the lagged distributed income, the duration of education, the rate of urbanization and year as the covariates [15–22].

### DALY estimation

We calculated the DALYs by summing the YLLs and YLDs for each cause, location, age group, sex, and year [4, 15]. The YLL corresponded to the number of deaths multiplied by the GBD 2015 reference life expectancy at the age of death by the cause-specific deaths to calculate the cause-specific YLLs. Numerous studies reported that the YLL was to certain extent proportional to YLD in the diseases which lead to both death and disability. YLDs of the diseases leading to both death and disability [23] was calculated based on the proportion for each cause, location, sex, age and year provided by the GBD 2015 study. YLDs of the diseases leading to disability only (but not death) were estimated based on the S-T LOESS model including the covariates of the lagged distributed income, the duration of education, the rate of urbanization and the provincial random effects. To ensure that the sum of YLDs in the lower level of the cause hierarchy equaled the upper level of the cause hierarchy, the YLDs were re-scaled to match the provincial-level YLD numbers in the GBD 2015 study. The cultural region level sex-specific YLD at different age-groups and city-level estimates were obtained at each year. (See Online Supplement).

The census population in 2000 in China was used as the reference population to calculate the age-standardized mortality and DALY with the direct standardization method, adjusting for the population structure. The test of Cochran-Armitage trend was adopted to examine the significance of the trends in all-cause and cause-specific mortality. Poisson regression model was conducted to explore the associations of deaths with socio-demographic factors of the year and cultural region.

### Life expectancy and healthy life expectancy estimation

Final estimates of age-sex-specific mortality for 2005–2015 were employed to compute abridged life tables developed by Sullivan et al. [24]. The same estimates of the YLDs per person for each location, age, sex, and year from 2005 to 2015 were used to calculate the HALE by the age group within abridged decrement life tables [25, 26]. For constructing the life tables, we utilized the estimated age schedules of mortality and disability. The interval of the age group used in the life tables was 5 years for an age group, except for the 0–1 and 1–4 year age groups. In the life expectancy calculation, the common version of the Sullivan life table method was applied. In the HALE calculation, we aimed to take into account the relationship between disability and the time to death. Annual age- and sex- specific years of the life adjusted estimates of the rate of YLD were used as the input to the life table. As the initial step, we stratified the life table population into subpopulations according to their age and sex at death (or the life span). The number of people in the subpopulation with the life span X equaled to the number who would die at the age of X according to the life table. Next, for each population with the same age at death, we estimated the sex- and age-specific schedule of disability. Finally, we estimated the sex-age-specific YLD rates for the total life table population. The total life expectancy and the years lived with disability rate were calculated for the aggregated life table population. Life expectancy and HALE at birth were reported for the three cultural regions.

## Results

### Trends of change in mortality in different regions

**Fig. 2:**
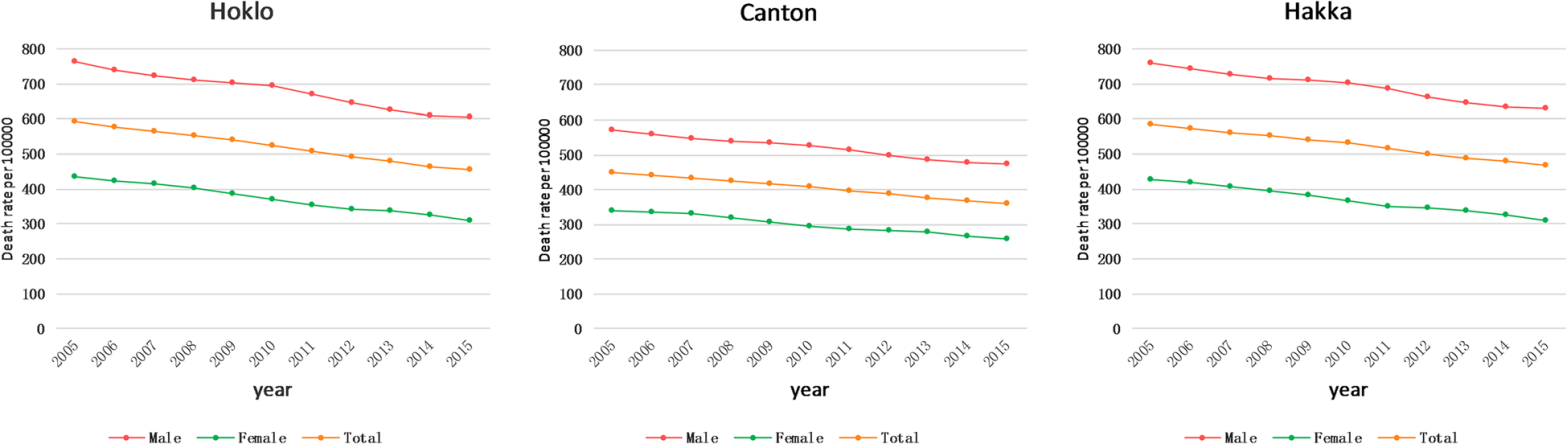
Age-standardized mortality rate by sex in three cultural regions in Guangdong between 2005 and 2015

The all-cause age-standardized mortality rates decreased significantly between 2005 and 2015 in Hakka (586.0 vs. 468.5 per 100,000 population), Canton (449.6 vs. 360.0 per 100,000 population) and Hoklo region (591.8 vs. 453.6 per 100,000 population) (all *P* < 0.05). Additionally, we noted significant differences in all-cause age-standardized mortality among the three cultural regions (*P* < 0.05) (Table 1, Fig. 2).

**Table 1:** Age-Standardized mortality rates per 100,000 people for 116 diseases in Hakka, Canton and Hoklo cultural regions, in 2005 and 2015

Cause of death	Hakka culture region			Canton culture region			Hoklo culture region			*P* value for region difference
	2005	2015	Change	2005	2015	Change	2005	2015	Change	
**All cause of death**	**585.96**	**468.52**	−20.04%^*^	**449.56**	**360.02**	−19.92%^*^	**591.81**	**453.63**	−23.35%^*^	**< 0.0001**^**#**^
**Communicable, maternal, neonatal, and nutritional diseases**	**37.49**	**32.91**	**−12.23%**	**27.13**	**23.74**	**−12.50%**^**#**^	**37.18**	**33.98**	**−8.61%**	**< 0.0001**^**#**^
**HIV/AIDS and tuberculosis**	**9.47**	**8.90**	**−6.02%**	**5.14**	**4.26**	**−17.29%**	**8.44**	**8.00**	**−5.18%**	**< 0.0001**^**#**^
Tuberculosis	8.22	7.21	−12.33%	3.55	2.37	−33.43%^*^	7.02	6.07	−13.50%	< 0.0001^**#**^
HIV/AIDS	1.25	1.69	35.48%^*^	1.59	1.89	18.76%^*^	1.42	1.93	35.94%^*^	0.023^**#**^
**Diarrhoea, lower respiratory and other common infectious diseases**	**18.57**	**15.69**	**−15.51%**	**15.14**	**12.53**	−17.26%^*^	**18.99**	**16.64**	−12.36%^*^	**0.081**
Diarrhoeal diseases	2.72	2.00	−26.56%	2.33	1.56	−33.13%^*^	3.05	2.25	−26.22%	0.012^**#**^
Intestinal infectious diseases	1.39	0.87	−36.96%^*^	1.11	0.80	−27.99%	1.35	0.93	−31.07%	0.047^**#**^
Lower respiratory infections	6.20	8.82	42.34%^*^	5.81	7.49	28.77%^*^	6.34	9.30	46.72%^*^	0.029^**#**^
Meningitis	4.28	2.39	−44.22%^*^	3.00	1.58	−47.32%^*^	4.70	2.63	−44.11%^*^	0.012^**#**^
Measles	2.89	0.91	−68.50%^*^	2.03	0.54	−73.45%^*^	2.47	0.77	−68.73%^*^	< 0.0001^**#**^
**Neglected tropical diseases and malaria**	**0.58**	**0.21**	−64.46%^*^	**0.57**	**0.28**	−50.63%^*^	**0.78**	**0.23**	−70.29%^*^	**< 0.0001**^**#**^
Malaria	0.04	0.03	−31.20%	0.04	0.02	−54.96%	0.04	0.03	−27.96%	0.531
Rabies	0.43	0.12	−71.99%^*^	0.43	0.18	−58.46%^*^	0.58	0.13	−77.01%^*^	< 0.0001^**#**^
Intestinal nematode infections	0.00	0.00	–	0.00	0.00	–	0.00	0.00	–	–
**Maternal disorders**	**0.21**	**0.05**	−74.73%^*^	**0.31**	**0.10**	−67.48%^*^	**0.23**	**0.06**	−74.73%^*^	**0.801**
**Neonatal disorders**	**5.42**	**5.87**	8.27%^*^	**4.15**	**5.30**	**27.69%**	**5.76**	**7.05**	**22.35%**	**0.084**
**Nutritional deficiencies**	**1.11**	**1.19**	7.55%^*^	**0.82**	**0.82**	**0.09%**	**1.07**	**1.13**	6.26%^*^	**< 0.002**^**#**^
Iron-deficiency anaemia	0.01	0.01	−2.44%	0.01	0.01	−16.07%	0.01	0.01	−0.82%	0.828
**Other communicable, maternal,neonatal, and nutritional diseases**	**2.14**	**1.00**	−53.14%^*^	**1.00**	**0.46**	−53.99%^*^	**1.92**	**0.87**	−54.73%^*^	**< 0.0001**^**#**^
Sexually transmitted diseases excluding HIV	0.42	0.21	−49.89%^*^	0.40	0.12	−70.19%^*^	0.44	0.23	−48.51%^*^	0.548
Hepatitis	1.53	0.64	−58.25%^*^	0.51	0.26	−49.61%^*^	1.30	0.50	−61.96%^*^	< 0.0001^**#**^
Acute hepatitis A	0.31	0.17	−45.87%	0.17	0.09	−49.44%^*^	0.31	0.15	−50.26%^*^	0.476
Acute hepatitis B	0.91	0.33	−63.40%^*^	0.21	0.11	−47.90%^*^	0.72	0.23	−67.30%^*^	< 0.0001^**#**^
Acute hepatitis C	0.12	0.06	−52.82%	0.06	0.02	−65.45%	0.12	0.04	−61.71%	0.253
**Non-communicable diseases**	**495.43**	**404.32**	−18.39%^*^	**388.94**	**315.46**	−18.89%^*^	**496.41**	**387.77**	−21.88%^*^	**< 0.0001**^**#**^
**Neoplasms**	**141.78**	**123.89**	**−12.62%**	**120.11**	**98.81**	**−17.74%**	**141.52**	**115.54**	−18.36%^*^	**< 0.0001**^**#**^
Esophageal cancer	8.02	7.13	−11.11%	5.92	4.58	−22.57%	7.39	6.01	−18.67%	< 0.0001^**#**^
Stomach cancer	15.87	14.24	−10.23%	12.34	9.38	−24.02%^*^	15.64	12.99	−16.93%	< 0.0001^**#**^
Liver cancer	28.67	23.59	−17.72%^*^	22.65	16.61	−26.69%^*^	30.13	22.66	−24.79%^*^	0.032^**#**^
Tracheal, bronchus, and lung cancer	34.72	26.62	−23.33%^*^	24.41	20.92	−14.29%	34.12	25.40	−25.55%^*^	< 0.0001^**#**^
Breast cancer	4.88	4.57	−6.25%	6.03	4.64	−23.12%	5.22	4.45	−14.73%	0.482
Cervical cancer	1.17	2.15	84.24%^*^	2.16	3.17	46.65%^*^	1.30	2.23	72.00%^*^	0.666
Uterine cancer	1.89	0.89	−53.11%^*^	1.45	0.59	−59.09%^*^	1.80	0.85	−52.68%^*^	< 0.0001^**#**^
Prostate cancer	0.36	0.36	0.04%	0.34	0.31	−8.77%	0.35	0.33	−4.49%	0.124
Colon and rectum cancer	6.47	8.15	25.93%^*^	7.63	8.01	5.05%^*^	6.30	7.27	15.27%^*^	< 0.0001^**#**^
Nasopharygeal cancer	12.62	9.90	−21.53%^*^	11.25	7.66	−31.93%^*^	12.19	8.75	−28.16%^*^	< 0.0001^**#**^
Pancreatic cancer	2.67	3.39	26.96%^*^	3.17	3.24	2.08%^*^	2.57	3.15	22.55%^*^	< 0.0001^**#**^
Ovarian cancer	1.27	1.52	19.18%^*^	2.05	1.90	−7.71%	1.30	1.46	11.63%^*^	0.005^**#**^
Kidney cancer	1.05	1.17	10.74%^*^	1.31	1.19	−9.13%	1.00	1.03	2.94%^*^	< 0.0001^**#**^
Bladder cancer	1.62	1.06	−34.70%^*^	1.33	0.77	−42.41%^*^	1.51	0.90	−40.49%	< 0.0001^**#**^
Thyroid cancer	0.83	0.69	−16.45%	1.03	0.71	−30.74%	0.92	0.71	−22.48%	0.945
Leukaemia	4.92	4.05	−17.66%^*^	4.32	3.42	−20.90%^*^	4.99	3.84	−23.11%^*^	0.119
**Cardiovascular diseases**	**199.58**	**170.86**	**−14.39%**	**159.57**	**136.96**	−14.17%^*^	**202.06**	**164.90**	−18.39%^*^	**< 0.0001**^**#**^
Rheumatic heart disease	5.80	3.06	−47.17%^*^	4.10	2.26	−45.03%^*^	5.89	2.96	−49.83%^*^	0.007^**#**^
Ischaemic heart disease	64.31	63.29	−1.59%^*^	61.72	60.51	−1.97%^*^	67.37	62.54	−7.17%^*^	< 0.0001^**#**^
**Cerebrovascular disease**	**111.69**	**85.13**	−23.77%^*^	**76.65**	**54.81**	−28.49%^*^	**109.93**	**79.71**	−27.49%^*^	**< 0.0001**^**#**^
Ischaemic stroke	45.92	33.63	−26.75%^*^	30.69	22.49	−26.72%^*^	45.24	29.95	−33.80%^*^	< 0.0001^**#**^
Haemorrhagic stroke	65.77	51.50	−21.70%^*^	45.96	32.32	−29.67%^*^	64.68	49.76	−23.08%	< 0.0001^**#**^
Hypertensive heart disease	10.07	12.06	19.84%^*^	11.21	13.71	22.33%^*^	11.04	12.59	13.99%^*^	0.086
Cardiomyopathy and myocarditis	1.59	1.38	−13.75%	0.99	0.88	−11.02%	1.63	1.34	−17.89%	0.351
Atrial fibrillation and flutter	1.34	1.62	21.04%^*^	1.24	1.45	17.29%^*^	1.35	1.60	18.19%^*^	0.036^**#**^
Aortic aneurysm	1.16	1.22	5.17%	0.85	0.89	4.90%^*^	1.17	1.16	−1.46%^*^	0.034^**#**^
**Chronic respiratory diseases**	**83.27**	**48.71**	−41.51%^*^	**53.79**	**29.80**	−44.61%^*^	**81.17**	**47.14**	−41.93%^*^	**< 0.0001**^**#**^
Chronic obstructive pulmonary disease	76.91	46.37	−39.72%^*^	50.90	28.74	−43.53%^*^	75.36	44.90	−40.43%^*^	< 0.0001^**#**^
Asthma	5.97	1.74	−70.80%^*^	2.68	0.72	−73.25%^*^	5.42	1.65	−69.52%^*^	< 0.0001^**#**^
**Cirrhosis**	**14.46**	**9.65**	−33.24%^*^	**8.35**	**5.44**	−34.90%^*^	**13.84**	**8.86**	−36.03%^*^	**< 0.0001**^**#**^
Cirrhosis due to alcohol use	0.00	0.00	–	0.00	0.00	–	0.00	0.00	–	–
**Digestive diseases**	**9.40**	**6.36**	−32.32%^*^	**5.86**	**3.83**	−34.66%^*^	**9.43**	**6.14**	−34.88%^*^	**< 0.0001**^**#**^
Peptic ulcer disease	3.91	2.34	−40.06%^*^	2.15	1.22	−43.39%^*^	3.98	2.31	−42.03%^*^	0.123
Pancreatitis	1.08	0.72	−33.61%	0.81	0.55	−32.98%	1.00	0.62	−37.45%	< 0.0001^**#**^
**Neurological disorders**	**14.08**	**16.99**	20.65%^*^	**16.29**	**18.51**	13.62%^*^	**14.15**	**17.03**	20.36%^*^	**< 0.0001**^**#**^
Alzheimer’s disease and other dementias	9.18	11.28	22.96%^*^	10.56	11.93	13.04%^*^	9.17	11.31	23.41%^*^	< 0.0001^**#**^
Parkinson’s disease	2.82	3.36	19.09%^*^	3.54	4.28	20.92%^*^	2.90	3.37	16.07%^*^	0.014^**#**^
Epilepsy	1.50	1.84	22.53%^*^	1.64	1.82	10.99%^*^	1.49	1.85	24.31%^*^	0.025^**#**^
Hemicrania	0.00	0.00	–	0.00	0.00	–	0.00	0.00	–	–
**Mental and substance use disorders**	**3.23**	**2.26**	−29.91%^*^	**1.55**	**1.15**	−26.08%^*^	**3.47**	**2.29**	−33.96%^*^	**0.310**
Schizophrenia	0.60	0.67	11.19%	0.44	0.46	4.63%	0.65	0.66	1.74%	0.791
Drug use disorders	1.70	1.06	−37.45%^*^	0.65	0.42	−36.22%^*^	1.82	1.09	−40.02%^*^	0.227
Depressive disorder	0.00	0.00	–	0.00	0.00	–	0.00	0.00	–	
Infantile autism	0.00	0.00	–	0.00	0.00	–	0.00	0.00	–	
**Diabetes, urogenital, blood, and endocrine diseases**	**24.05**	**19.52**	−18.82%^*^	**18.40**	**14.96**	**−18.73%**	**24.72**	**19.14**	**−22.59%**	**< 0.0001**^**#**^
Diabetes mellitus	8.64	6.41	−25.76%^*^	6.05	4.38	−27.70%^*^	8.65	6.06	−30.02%^*^	< 0.0001^**#**^
Chronic kidney disease	9.56	8.77	−8.19%	7.95	6.95	−12.52%	10.18	8.88	−12.79%^*^	0.224
Gynaecological diseases	0.46	0.35	−23.50%	0.36	0.39	7.04%^*^	0.45	0.34	−23.73%	0.305
Haemoglobinopathies and haemolytic anaemias	2.54	1.64	−35.39%^*^	1.88	1.40	−25.51%	2.49	1.54	−37.94%	0.001^**#**^
**Musculoskeletal disorders**	**1.09**	**0.89**	**−17.82%**	**1.09**	**0.85**	−22.33%^*^	**1.08**	**0.84**	**−22.52%**	**0.047**^**#**^
Rheumatoid arthritis	0.43	0.36	−15.99%	0.42	0.34	−18.66%	0.43	0.34	−18.97%	0.146
Osteoarthritis	0.00	0.00	–	0.00	0.00	–	0.00	0.00	–	
Low back and neck pain	0.00	0.00	–	0.00	0.00	–	0.00	0.00	–	
Low back pain	0.00	0.00	–	0.00	0.00	–	0.00	0.00	–	
Neck pain	0.00	0.00	–	0.00	0.00	–	0.00	0.00	–	
**Other non-communicable diseases**	**4.50**	**5.18**	15.07%^*^	**3.93**	**5.16**	**31.57%**	**4.96**	**5.90**	**18.88%**	**0.150**
Congenital anomalies	4.26	4.97	16.51%^*^	3.70	4.91	32.86%	4.68	5.57	19.10%	0.429
Skin and subcutaneous diseases	0.20	0.16	−20.98%	0.19	0.19	−0.65%	0.24	0.26	8.82%	< 0.04^**#**^
Sense organ diseases	0.00	0.00	–	0.00	0.00	–	0.00	0.00	–	
Oral disorders	0.00	0.00	–	0.00	0.00	–	0.00	0.00	–	
**Injuries**	**53.03**	**31.29**	−40.99%^*^	**33.48**	**20.83**	−37.79%^*^	**58.23**	**31.87**	−45.26%^*^	**< 0.0001**^**#**^
**Transport injuries**	**23.03**	**13.17**	−42.81%^*^	**14.21**	**8.07**	−43.17%^*^	**25.68**	**13.00**	−49.37%^*^	**< 0.0001**^**#**^
Road injuries	21.62	12.62	−41.61%^*^	13.70	7.75	−43.44%^*^	23.94	12.32	−48.54%^*^	0.001^**#**^
Other transport injuries	1.41	0.55	−61.15%^*^	0.51	0.33	−35.75%^*^	1.74	0.68	−60.78%^*^	< 0.0001^**#**^
**Unintentional injuries**	**17.58**	**12.28**	−30.17%^*^	**13.62**	**9.63**	−29.30%^*^	**19.69**	**13.20**	−32.97%^*^	**< 0.0001**^**#**^
Falls	4.54	4.05	−10.63%	3.87	3.43	−11.33%	5.09	4.31	−15.22%	0.002^**#**^
Drowning	5.17	3.17	−38.55%^*^	4.23	2.56	−39.48%^*^	5.78	3.39	−41.31%^*^	< 0.0001^**#**^
Fire, heat, and hot substances	1.29	0.66	−48.48%^*^	0.80	0.46	−41.83%^*^	1.45	0.74	−49.38%^*^	0.009^**#**^
Poisonings	2.29	1.38	−40.06%^*^	1.38	0.83	−39.93%^*^	2.54	1.46	−42.58%^*^	0.013^**#**^
**Intentional injury**	**12.21**	**5.76**	−52.82%^*^	**5.51**	**3.06**	−44.43%^*^	**12.62**	**5.59**	−55.72%^*^	**0.692**
Self-harm	9.17	4.54	−50.53%^*^	4.33	2.49	−42.55%^*^	9.51	4.38	−53.96%^*^	0.682
Interpersonal violence	3.04	1.22	−59.75%^*^	1.18	0.57	−51.36%^*^	3.11	1.21	−61.10%^*^	0.108
Other diarrhea, lower respiratory and common infectious disease	1.09	0.70	−36.03%^*^	0.86	0.57	−34.39%^*^	1.09	0.77	−29.62%^*^	0.552
Other neglected tropical disease	0.11	0.06	−47.25%	0.10	0.09	−15.50%	0.15	0.07	−56.55%	0.088
Other nutritional disease	1.10	1.18	7.64%^*^	0.81	0.81	0.28%	1.06	1.12	6.32%^*^	< 0.02^**#**^
Other hepatitis	0.19	0.08	−57.14%^*^	0.07	0.04	−41.87%	0.16	0.06	−60.61%^*^	0.071
Other Communicable, maternal, neonatal, and nutritional diseases	0.19	0.15	−19.36%	0.09	0.09	−6.81%	0.18	0.15	−17.00%	0.352
Other cancer	14.75	14.41	−2.29%^*^	12.71	11.72	−7.82%^*^	14.79	13.50	−8.71%^*^	< 0.0001^**#**^
Other cardiovascular disease	3.62	3.09	−14.54%	2.80	2.45	−12.58%	3.67	3.02	−17.92%	< 0.002^**#**^
Other chronic respiratory disease	0.39	0.60	54.58%^*^	0.21	0.34	56.48%^*^	0.39	0.59	52.51%^*^	0.509
Other Cirrhosis	14.46	9.65	−33.24%^*^	8.35	5.44	−34.90%^*^	13.84	8.86	−36.03%^*^	< 0.0001^**#**^
Other digestive disease	4.41	3.30	−25.15%^*^	2.89	2.06	−28.62%^*^	4.45	3.21	−27.92%	0.008^**#**^
Other neurological disorders	0.58	0.50	−13.09%	0.55	0.47	−14.13%	0.59	0.49	−16.10%	0.682
Other mental and substance use disorders	0.93	0.53	−42.68%^*^	0.46	0.27	−40.83%^*^	0.99	0.53	−46.26%^*^	0.558
Other diabetes, urogenital, blood, and endocrine diseases	2.85	2.34	−17.87%	2.17	1.84	−14.91%	2.95	2.32	−21.49%	0.185^**#**^
Other musculoskeletal disorders	0.65	0.53	−19.03%	0.67	0.51	−24.62%^*^	0.65	0.49	−24.82%	0.164
Others	0.04	0.06	39.49%	0.03	0.06	72.40%	0.05	0.07	48.52%	0.742
Other unintentional injury	4.30	3.01	−29.94%^*^	3.35	2.35	−29.80%^*^	4.83	3.30	−31.68%^*^	0.014^**#**^
Other injury	0.21	0.08	−61.06%^*^	0.14	0.06	−57.37%^*^	0.23	0.09	−63.48%^*^	0.527

^#^Statistical significance using Poisson regression model for comparison of different culture regions (*P* < 0.05)

^*^Statistical significance using the test of Cochran-Armitage trend for year 2005 to 2015 comparison (*P* < 0.05)

From 2005 to 2015, the mortality of communicable, maternal, neonatal, and nutritional diseases (CMNN) decreased by 12.2, 12.5 and 8.6% in Hakka, Canton and Hoklo region, with the reduction being statistically significant in Canton region only (*P* < 0.05). The CMNN mortality differed significantly among the three cultural regions, with Canton being the lowest, followed by Hoklo and Hakka regions (in terms of tuberculosis, meningitis, measles, hepatitis and nutritional deficiency). In addition, the mortality also differed considerably for diarrhea, intestinal infectious diseases, and lower respiratory infections, with Hoklo the highest, followed by Hakka and Canton region (Table 1).

We noted the lowest age-standardized mortality of non-communicable diseases (NCD) between 2005 and 2015 (388.9 vs. 315.5 per 100,000 population) in Canton region, compared with Hakka (495.4 vs. 404.3 per 100,000 population) and Hoklo regions (496.4 vs. 387.8 per 100,000 population). The corresponding reduction was − 18.9, − 18.4% and − 21.9%, respectively (all *P* < 0.05). Of all cardiovascular diseases, we observed a consistently significant reduction in the mortality of rheumatic heart disease, ischemic heart disease, ischemic stroke and hemorrhagic stroke in the three cultural regions (all *P* < 0.05). Additionally, the mortality of cardiovascular diseases of Canton (159.6 vs. 137.0 per 100,000 population) region was significantly lower than that of Hakka (199.6 vs. 170.9 per 100,000 population) and Hoklo (202.1 vs. 164.8 per 100,000 population) regions in 2005 and 2015. We noted a marked difference in the mortality of cancer of all organs among the three cultural regions, except for breast cancer, cervical cancer, prostate cancer, thyroid cancer and leukemia. The mortality of cancer was lowest in Canton region, followed by Hoklo and Hakka region (*P* < 0.0001). Notably, we noted a high mortality for nasopharyngeal, esophageal, tracheal, bronchus and lung cancer, liver and stomach cancer in Hakka and Hoklo regions in both 2005 and 2015. Furthermore, the mortality of chronic respiratory diseases, cirrhosis, digestive diseases, neurological disorders, mental and substance use disorders, and diabetes mellitus differed significantly among the three regions. In contrast to most of the other NCDs, mortality rates of neurological disorders were highest in the Canton region compared with the other two regions (Table 1). Details of sex-specific mortality and DALY rates are demonstrated in Online Supplement (E-Tables 1-4).

There was a marked reduction in injury-related mortality at all cultural regions from 2005 to 2015, particularly transport injuries, drowning, fire, heat and hot substances, poisoning and intentional injury. Except for intentional injuries, the mortality in the all-cause and cause-specific injury differed significantly across the three cultural regions in both 2005 and 2015, especially road injury [(Hakka (21.6 vs. 12.6 per 100,000 population in both 2005 and 2015) vs. Canton (13.7 vs. 7.8 per 100,000 population) vs. Hoklo region (23.9 vs. 12.3 per 100,000 population)] (Table 1).

**Fig. 3:**
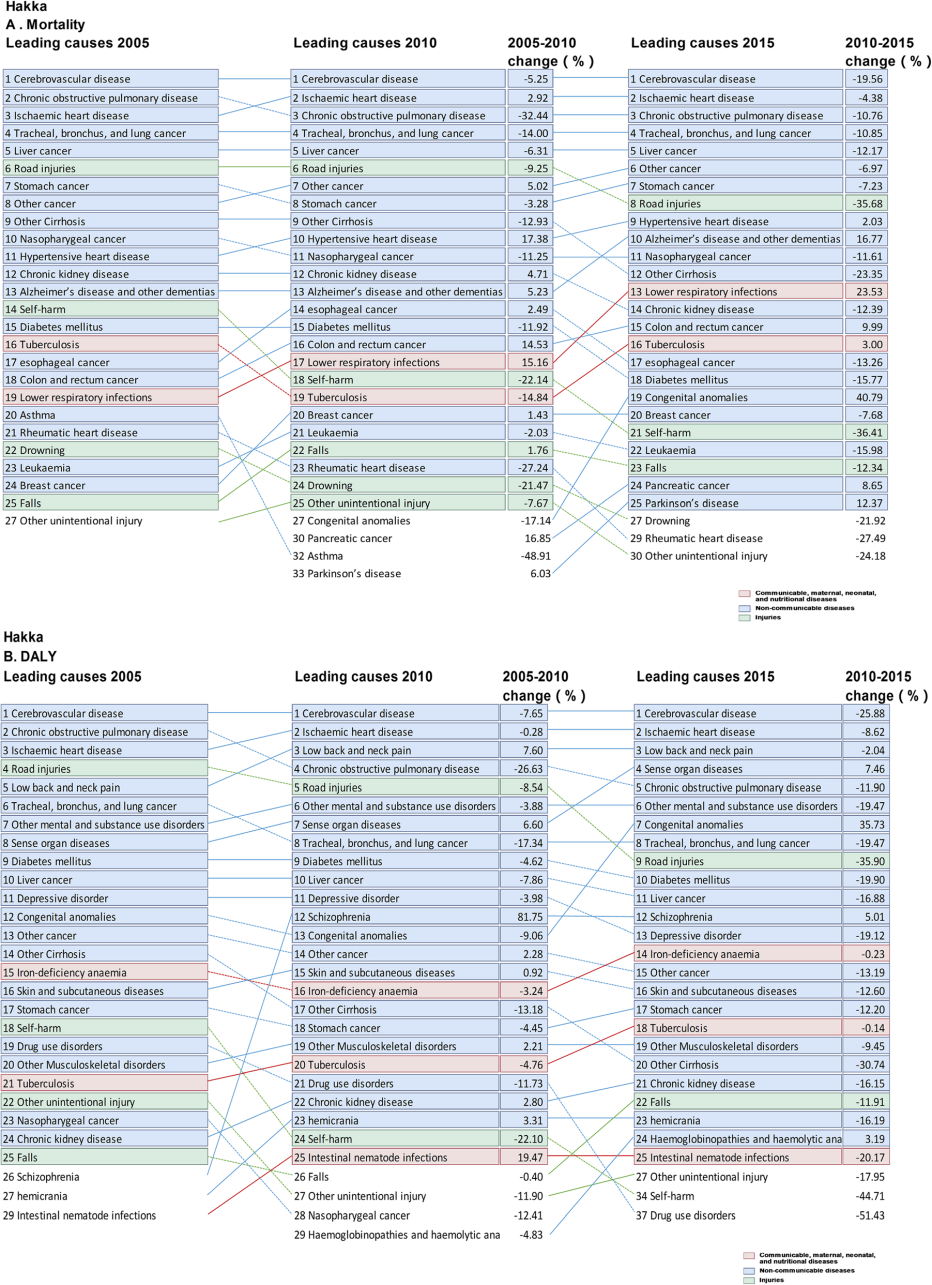
Top 25 causes of age-standardized mortality and DALY rates in Hakka culture region in Guangdong province, with the median percentage change from 2005 to 2015. **a** Age-standardized mortality; **b** DALYs: Age-standardized disability-adjusted life-years

**Fig. 4:**
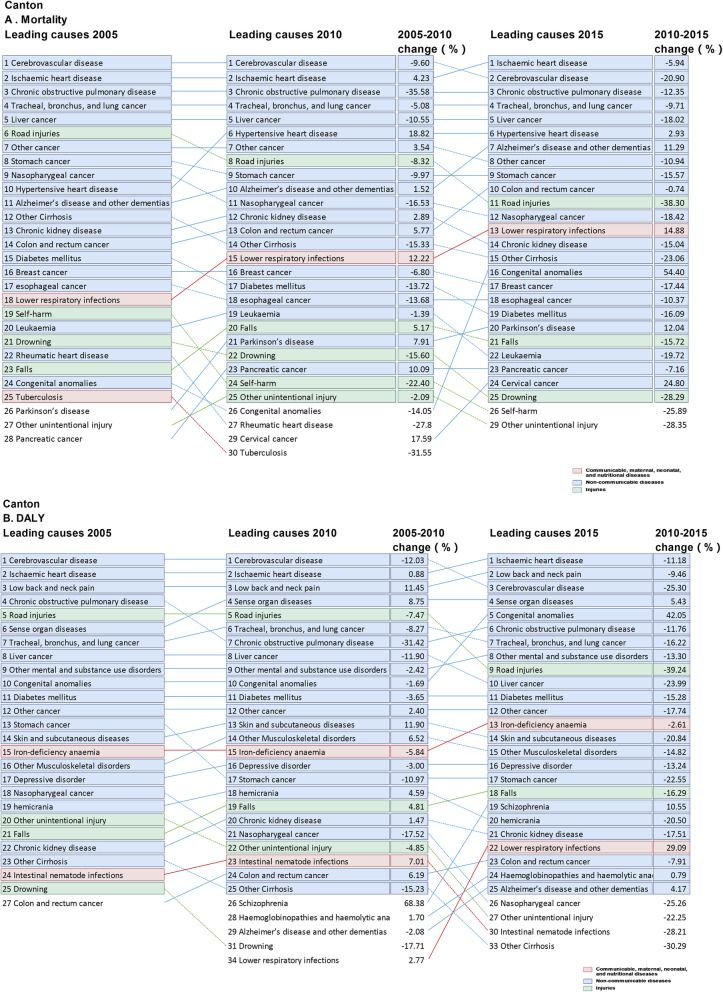
Top 25 causes of mortality and DALY rates in Canton culture region in Guangdong province, with the median percentage change from 2005 to 2015. **a** Age-standardized mortality; **b** DALYs: Age-standardized disability-adjusted life-years

**Fig. 5:**
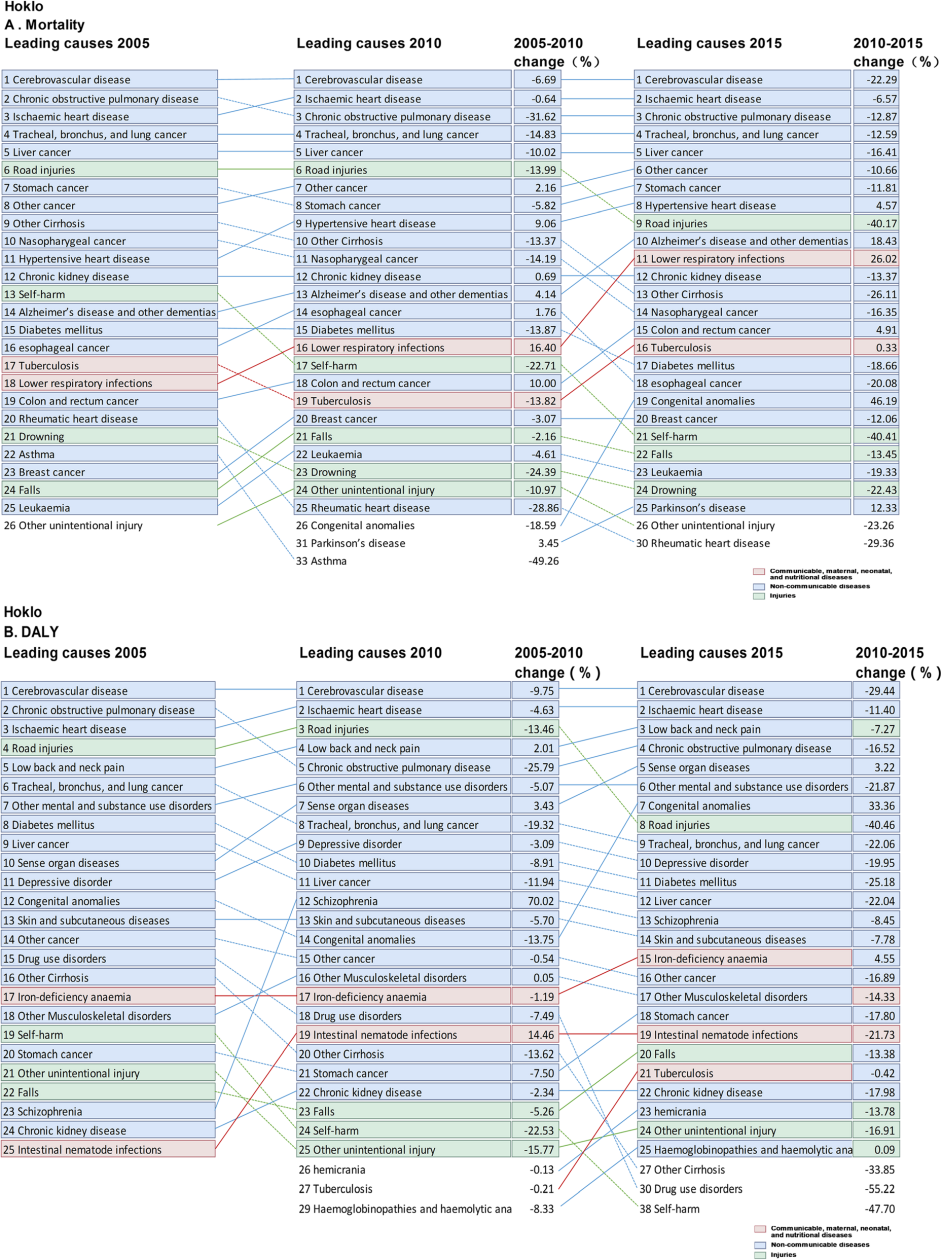
Top 25 causes of mortality and DALY rates in Hoklo culture region in Guangdong province, with the median percentage change from 2005 to 2015. **a** Age-standardized mortality; **b** DALYs: Age-standardized disability-adjusted life-years

There were slight differences in the spectra of the top 10 causes of death among the three regions, with unique leading causes of death within each region. For instance, Alzheimer’s disease ranked top 10 in Canton region only, while chronic kidney disease ranked top 10 in Hoklo and Hakka regions in 2005. In 2015, Alzheimer’s disease became the top 7 cause of death in Canton region, and the ranking of hypertensive heart disease elevated in all cultural regions. Notably, colorectal cancer and lower respiratory infections became the exclusive top 10 cause of death in Canton and Hoklo region in 2015, respectively (Figs. 3, 4 and 5).

The trends of change of all-cause and cause specific mortality in males and females in the three cultural regions were similar to those of the whole study population (E-Figure 1-2).

### Changes in the leading causes of DALY over time

The total age-standardized DALY rates in 2005 were 25,738.3, 17,093.9 and 24,871.8 in Hakka, Canton and Hoklo region, respectively. The corresponding reduction was 22.3, 15.8 and 17.8% from 2005 to 2015. The DALY rates for CMNN fell by 4.5, 9.4 and 10.0% in Hakka, Canton and Hoklo regions, but the reductions of CMNN in Hakka and Hoklo regions were less remarkable for the other DALY rates from 2005 to 2015. The proportion of NCDs accounting for all DALY rates increased, remaining the dominant leading cause of DALY over time in all cultural regions, while injuries decreased. The total DALY rates for NCDs and injury were lower in Canton region than in Hoklo and Hakka regions, between 2005 and 2015. Despite the overall lowering DALY rates for CMNN, NCD and injuries, we noted a major increase in the DALY for some diseases: HIV/AIDS, lower respiratory infections, cervical cancer, atrial fibrillation, epilepsy, schizophrenia, Alzheimer’s disease and dementia, congenital anomalies and sensory organ diseases in the three regions. The DALY rates of tuberculosis, diarrheal diseases, meningitis, measles, neglected tropical diseases, hepatitis, neonatal disorders, nutritional deficiency and lower respiratory infections were lower in Canton region than in Hakka and Hoklo regions. Likewise, the DALY rates of ischemic heart disease, cerebrovascular diseases, cancers (including esophageal, nasopharyngeal, esophageal, tracheal, bronchus, and lung cancer, liver, uterine, bladder and stomach cancer), chronic respiratory diseases, cirrhosis, diabetes, injuries and specially mental and substance use disorders in Hakka and Hoklo regions were significantly higher than in Canton region (Table 2, Figs. 3, 4 and 5).

**Table 2:** Age-standardized disability-adjusted life-years (DALY) rate (per 100,000) for 116 diseases in Hakka, Canton and Hoklo culture regions in 2005 and 2015

All cause of death	Hakka culture region			Canton culture region			Hoklo culture region			*P* value
	2005	2015	Change	2005	2015	Change	2005	2015	Change	
**All cause of death**	**25,738.30**	**19,988.00**	−22.34^*^	**17,093.90**	**14,396.50**	−15.78^*^	**24,871.80**	**20,436.60**	−17.83^*^	**< 0.0001**^#^
**Communicable, maternal, neonatal, and nutritional diseases**	**2790.66**	**2666.13**	−4.46^*^	**1990.19**	**1803.29**	−9.39^*^	**2795.57**	**2515.09**	−10.03^*^	**< 0.0001**^#^
**HIV/AIDS and tuberculosis**	**330.86**	**358.49**	**8.35**	**196.40**	**179.54**	−8.58^*^	**383.21**	**388.06**	1.27^*^	**< 0.0001**^#^
Tuberculosis	288.13	286.30	−0.64^*^	141.89	106.78	−24.74^*^	345.39	328.49	−4.89^*^	< 0.0001^#^
HIV/AIDS	42.73	72.19	68.94^*^	54.51	72.75	33.46^*^	37.83	59.57	57.47^*^	< 0.0001^#^
**Diarrhoea, lower respiratory and other common infectious diseases**	**711.32**	**597.00**	−16.07^*^	**531.49**	**410.33**	−22.80^*^	**718.61**	**541.28**	−24.68^*^	**0.062**
Diarrhoeal diseases	101.27	90.23	−10.90^*^	68.42	52.25	−23.63^*^	80.26	71.07	−11.45^*^	< 0.0001^#^
Intestinal infectious diseases	13.89	10.00	−28.01^*^	12.93	9.86	− 23.74^*^	13.76	8.45	−38.59^*^	0.724
Lower respiratory infections	164.06	259.81	58.36^*^	144.61	191.86	32.67^*^	155.69	229.19	47.21^*^	< 0.0001^#^
Meningitis	117.92	71.61	−39.27^*^	72.30	42.32	−41.47^*^	100.06	58.64	−41.40^*^	< 0.0001^#^
Measles	172.89	50.61	−70.73^*^	126.39	30.16	−76.14^*^	202.08	59.95	−70.33^*^	< 0.0001^#^
**Neglected tropical diseases and malaria**	**520.70**	**400.65**	−23.06^*^	**337.72**	**271.49**	−19.61^*^	**417.23**	**357.02**	−14.43^*^	**< 0.0001**^#^
Malaria	1.74	1.03	−40.80^*^	1.83	0.66	−63.93^*^	1.65	0.89	−46.06^*^	< 0.0001^#^
Rabies	28.54	6.61	−76.84^*^	26.22	12.07	−53.97^*^	19.93	5.70	−71.40^*^	< 0.0001^#^
Intestinal nematode infections	333.62	298.88	−10.41^*^	199.42	153.19	−23.18^*^	256.50	244.64	−4.62^*^	< 0.0001^#^
**Maternal disorders**	**14.89**	**5.29**	−64.47^*^	**20.99**	**7.50**	−64.27^*^	**14.64**	**5.02**	−65.71^*^	**0.279**
**Neonatal disorders**	**614.72**	**732.54**	19.17^*^	**491.65**	**578.33**	17.63^*^	**625.34**	**654.49**	4.66^*^	**< 0.0001**^#^
**Nutritional deficiencies**	**474.19**	**490.59**	3.46^*^	**342.68**	**316.20**	−7.73^*^	**499.87**	**485.01**	−2.97^*^	**< 0.0001**^#^
Iron-deficiency anaemia	422.28	436.27	3.31^*^	299.59	274.73	−8.30^*^	447.77	432.29	−3.46^*^	< 0.0001^#^
**Other communicable, maternal,neonatal, and nutritional diseases**	**123.99**	**81.57**	−34.21^*^	**69.27**	**39.91**	−42.38^*^	**136.67**	**84.21**	−38.38^*^	**< 0.0001**^#^
Sexually transmitted diseases excluding HIV	51.81	31.84	−38.54^*^	38.54	13.25	−65.62^*^	56.05	34.00	−39.34^*^	< 0.0001^#^
Hepatitis	47.47	24.63	−48.11^*^	20.43	16.04	−21.49^*^	55.10	27.38	−50.31^*^	< 0.0001^#^
Acute hepatitis A	11.93	10.25	−14.08^*^	7.32	5.21	−28.83^*^	12.38	10.68	−13.73^*^	0.062
Acute hepatitis B	26.53	10.32	−61.10^*^	8.40	7.69	−8.45^*^	32.73	11.99	−63.37^*^	< 0.0001^#^
Acute hepatitis C	3.17	1.26	−60.25^*^	1.75	0.57	−67.43^*^	3.29	1.60	−51.37^*^	< 0.0001^#^
**Non-communicable diseases**	**19,758.00**	**15,582.50**	−21.13^*^	**13,383.90**	**11,525.90**	−13.88^*^	**19,227.10**	**16,256.30**	−15.45^*^	**< 0.0001**^#^
**Neoplasms**	**3948.53**	**2891.63**	−26.77^*^	**3194.09**	**2397.33**	−24.94^*^	**3941.57**	**3142.67**	−20.27^*^	**< 0.0001**^#^
esophageal cancer	183.09	134.50	−26.54^*^	142.85	99.42	−30.40^*^	199.78	164.78	−17.52^*^	< 0.0001^#^
Stomach cancer	405.49	308.33	−23.96^*^	310.70	214.23	−31.05^*^	411.30	345.05	−16.11^*^	< 0.0001^#^
Liver cancer	802.96	551.27	−31.35^*^	590.71	395.57	−33.03^*^	763.51	584.75	−23.41^*^	0.001^#^
Tracheal, bronchus, and lung cancer	995.58	626.03	−37.12^*^	654.80	503.17	−23.16^*^	1005.01	668.99	−33.43^*^	< 0.0001^#^
Breast cancer	145.22	121.48	−16.35^*^	163.21	121.10	−25.80^*^	135.40	125.60	−7.24^*^	0.747
Cervical cancer	29.21	52.75	80.59^*^	48.78	72.70	49.04^*^	26.22	50.55	92.79^*^	0.010^#^
Uterine cancer	44.14	18.29	−58.56^*^	31.45	9.99	−68.24^*^	46.82	19.00	−59.42^*^	< 0.0001^#^
Prostate cancer	8.30	6.82	−17.83	8.20	6.31	−23.05^*^	8.55	7.84	−8.30	< 0.0001^#^
Colon and rectum cancer	156.54	169.17	8.07^*^	187.59	183.46	−2.20^*^	161.73	194.72	20.40^*^	< 0.0001^#^
Nasopharygeal cancer	315.14	206.17	−34.58^*^	286.20	176.43	−38.35^*^	327.84	239.86	−26.84^*^	< 0.0001^#^
Pancreatic cancer	58.98	67.11	13.78^*^	71.78	66.31	−7.62^*^	61.90	74.10	19.71^*^	< 0.0001^#^
Ovarian cancer	29.13	32.11	10.23^*^	45.56	39.85	−12.53	28.77	33.66	17.00^*^	< 0.0001^#^
Kidney cancer	20.87	19.51	−6.52^*^	27.22	21.81	−19.88^*^	22.51	23.18	2.98^*^	< 0.0001^#^
Bladder cancer	34.62	17.30	−50.03^*^	28.81	13.64	−52.66^*^	37.86	21.62	−42.89^*^	< 0.0001^#^
Thyroid cancer	20.16	13.61	−32.49^*^	21.45	12.54	−41.54^*^	18.19	13.46	−26.00^*^	0.273
Leukaemia	228.80	158.44	−30.75^*^	194.89	140.81	−27.75^*^	224.07	165.17	−26.29^*^	< 0.0001^#^
**Cardiovascular diseases**	**4292.37**	**3093.99**	−27.92^*^	**2908.59**	**2274.07**	−21.82^*^	**4218.82**	**3249.63**	−22.97^*^	**< 0.0001**^#^
Rheumatic heart disease	196.97	93.83	−52.36^*^	104.52	56.33	−46.11^*^	191.94	97.25	−49.33^*^	0.029^#^
Ischaemic heart disease	1372.88	1160.05	−15.50^*^	1152.25	1032.42	−10.40^*^	1310.31	1193.98	−8.88^*^	< 0.0001^#^
**Cerebrovascular disease**	**2321.07**	**1478.05**	−36.32^*^	**1369.14**	**899.75**	−34.28^*^	**2337.35**	**1599.92**	−31.55^*^	**< 0.0001**^#^
Ischaemic stroke	962.06	597.31	−37.91^*^	560.86	392.07	−30.09^*^	967.38	675.00	−30.22^*^	< 0.0001^#^
Haemorrhagic stroke	1359.01	880.74	−35.19^*^	808.29	507.69	−37.19^*^	1369.97	924.92	−32.49^*^	< 0.0001^#^
Hypertensive heart disease	171.87	164.18	−4.47^*^	145.62	154.92	6.39^*^	155.44	156.60	0.75^*^	< 0.0001^#^
Cardiomyopathy and myocarditis	67.02	56.00	−16.44^*^	31.45	28.65	−8.90^*^	64.62	55.30	−14.42^*^	0.001^#^
Atrial fibrillation and flutter	47.78	56.10	17.41^*^	35.69	44.47	24.60^*^	46.92	58.21	24.06^*^	< 0.0001^#^
Aortic aneurysm	29.87	25.59	−14.33	18.44	17.56	−4.77^*^	29.29	27.46	−6.25	< 0.0001^#^
**Chronic respiratory diseases**	**1637.99**	**1000.40**	−38.93^*^	**904.81**	**545.24**	−39.74^*^	**1656.14**	**1041.28**	−37.13^*^	**< 0.0001**^#^
Chronic obstructive pulmonary disease	1426.92	884.04	−38.05^*^	835.85	505.83	−39.48^*^	1449.62	937.06	−35.36^*^	< 0.0001^#^
Asthma	192.13	89.54	−53.40^*^	61.23	26.79	−56.25^*^	188.97	79.16	−58.11^*^	< 0.0001^#^
**Cirrhosis**	**435.60**	**249.30**	−42.77^*^	**228.36**	**135.34**	−40.73^*^	**455.87**	**274.54**	−39.78^*^	**< 0.0001**^#^
Cirrhosis due to alcohol use	0.75	0.82	9.33^*^	0.72	0.82	13.89^*^	0.77	0.88	14.29^*^	0.014^#^
**Digestive diseases**	**396.72**	**278.26**	−29.86^*^	**199.95**	**149.06**	−25.45^*^	**384.09**	**289.64**	−24.59^*^	**< 0.0001**^#^
Peptic ulcer disease	134.96	73.14	−45.81^*^	61.65	34.26	−44.43^*^	129.65	74.93	−42.21^*^	0.551
Pancreatitis	21.22	12.08	−43.07^*^	15.88	9.48	− 40.30^*^	23.23	14.37	−38.14^*^	< 0.0001^#^
**Neurological disorders**	**736.75**	**699.57**	−5.05^*^	**668.45**	**631.02**	−5.60^*^	**725.72**	**698.53**	−3.75^*^	**< 0.0001**^#^
Alzheimer’s disease and other dementias	159.18	171.88	7.98^*^	173.57	177.04	2.00^*^	159.87	174.48	9.14^*^	< 0.0001^#^
Parkinson’s disease	74.07	70.28	−5.12^*^	80.65	81.91	1.56^*^	73.22	71.84	−1.88^*^	< 0.0001^#^
Epilepsy	67.32	81.31	20.78^*^	64.49	78.07	21.06^*^	65.54	80.67	23.09^*^	< 0.0001^#^
hemicrania	307.23	264.57	−13.89^*^	240.39	199.87	−16.86^*^	300.10	259.84	−13.42^*^	< 0.0001^#^
**Mental and substance use disorders**	**2731.12**	**2193.60**	−19.68^*^	**1022.46**	**960.20**	−6.09^*^	**2524.35**	**2177.44**	−13.74^*^	**< 0.0001**^#^
Schizophrenia	346.57	539.44	55.65^*^	111.62	207.78	86.15^*^	303.54	579.32	90.85^*^	< 0.0001^#^
Drug use disorders	449.50	186.23	−58.57^*^	62.12	30.01	−51.69^*^	371.76	159.38	−57.13^*^	< 0.0001^#^
Depressive disorder	778.66	604.07	−22.42^*^	287.74	242.16	−15.84^*^	729.79	566.76	−22.34^*^	< 0.0001^#^
infantile autism	174.82	135.85	−22.29^*^	83.89	76.65	−8.63^*^	162.63	131.51	−19.14^*^	< 0.0001^#^
**Diabetes, urogenital, blood, and endocrine diseases**	**1779.26**	**1384.84**	−22.17^*^	**1075.26**	**946.26**	−12.00^*^	**1665.06**	**1412.85**	−15.15^*^	**< 0.0001**^#^
Diabetes mellitus	820.07	558.89	−31.85^*^	419.41	342.39	−18.36^*^	772.48	590.16	−23.60^*^	< 0.0001^#^
Chronic kidney disease	345.98	277.13	−19.90^*^	234.71	196.45	−16.30^*^	317.28	273.49	−13.80^*^	< 0.0001^#^
Gynaecological diseases	153.83	148.49	−3.47^*^	107.62	99.16	−7.86 ^*^	144.09	150.96	4.77^*^	0.244
Haemoglobinopathies and haemolytic anaemias	283.33	259.98	−8.24^*^	174.98	179.36	2.50^*^	260.54	255.86	−1.80^*^	< 0.0001^#^
**Musculoskeletal disorders**	**1694.31**	**1616.82**	−4.57^*^	**1481.89**	**1512.38**	2.06^*^	**1657.90**	**1760.05**	6.16^*^	**< 0.0001**^#^
Rheumatoid arthritis	56.21	61.70	9.77^*^	46.44	55.64	19.81^*^	55.89	73.18	30.94^*^	< 0.0001^#^
Osteoarthritis	147.59	182.70	23.79^*^	126.18	165.03	30.79^*^	153.44	205.27	33.78^*^	< 0.0001^#^
Low back and neck pain	1068.61	1010.80	−5.41^*^	1019.89	1029.16	0.91^*^	1096.16	1155.43	5.41^*^	< 0.0001^#^
Low back pain	569.02	533.84	−6.18^*^	548.66	555.13	1.18^*^	583.39	611.18	4.76^*^	< 0.0001^#^
Neck pain	499.60	476.95	−4.53^*^	471.23	474.03	0.59^*^	512.77	544.25	6.14^*^	< 0.0001^#^
**Other non-communicable diseases**	**2105.39**	**2174.14**	3.27^*^	**1700.02**	**1974.99**	16.17^*^	**1997.56**	**2209.71**	10.62^*^	**< 0.0001**^#^
Congenital anomalies	584.40	672.16	15.02^*^	446.03	622.86	39.65^*^	547.95	676.35	23.43^*^	< 0.0001^#^
Skin and subcutaneous diseases	542.34	471.68	−13.03^*^	310.09	274.66	−11.43^*^	435.85	384.40	−11.80^*^	< 0.0001^#^
Sense organ diseases	795.19	848.91	6.76^*^	704.87	808.17	14.66^*^	834.64	956.11	14.55^*^	< 0.0001^#^
Oral disorders	179.49	175.50	−2.22^*^	140.20	152.86	9.03^*^	175.63	187.98	7.03^*^	< 0.0001^#^
**Injuries**	**3189.58**	**1739.29**	−45.47^*^	**1719.80**	**1067.36**	−37.94^*^	**2849.14**	**1665.14**	− 41.56^*^	**< 0.0001**^#^
**Transport injuries**	**1362.87**	**692.02**	−49.22^*^	**734.36**	**417.83**	−43.10^*^	**1201.41**	**688.81**	−42.67^*^	**< 0.0001**^#^
Road injuries	1262.68	650.63	−48.47^*^	709.54	398.92	−43.78^*^	1121.45	657.52	−41.37^*^	< 0.0001^#^
Other transport injuries	100.19	41.39	−58.69^*^	24.82	18.91	−23.81^*^	79.97	31.29	−60.87^*^	< 0.0001^#^
**Unintentional injuries**	**1206.87**	**796.01**	−34.04^*^	**760.03**	**537.22**	−29.32^*^	**1050.09**	**721.60**	−31.28^*^	**< 0.0001**^#^
Falls	351.26	288.25	−17.94^*^	237.65	208.51	−12.26^*^	306.90	269.26	−12.26^*^	< 0.0001^#^
Drowning	284.75	153.98	−45.92^*^	196.80	107.00	−45.63^*^	247.19	135.73	−45.09^*^	< 0.0001^#^
Fire, heat, and hot substances	84.61	40.09	−52.62^*^	37.98	21.51	−43.36^*^	71.74	33.44	−53.39^*^	< 0.0001^#^
Poisonings	108.29	49.18	−54.58^*^	49.20	23.83	−51.57^*^	93.20	43.87	−52.93^*^	< 0.0001^#^
**Intentional injury**	**604.52**	**245.09**	−59.46^*^	**217.08**	**108.35**	−50.09^*^	**583.96**	**248.85**	−57.39^*^	**< 0.0001**^#^
Self-harm and interpersonal violence	412.28	167.03	−59.49^*^	150.58	74.45	−50.56^*^	396.15	170.64	−56.93^*^	< 0.0001^#^
Interpersonal violence	192.24	78.05	−59.40^*^	66.50	33.90	−49.02^*^	187.81	78.22	−58.35^*^	< 0.0001^#^
Other diarrhea, lower respiratory and common infectious disease	141.29	114.75	−18.78^*^	106.84	83.87	−21.50^*^	166.76	113.99	−31.64^*^	< 0.0001^#^
Other neglected tropical disease	156.80	94.13	−39.97^*^	110.26	105.57	−4.25^*^	139.16	105.79	−23.98^*^	< 0.0001^#^
Other nutritional disease	51.91	54.32	4.64^*^	43.09	41.47	−3.76^*^	52.11	52.72	1.17^*^	< 0.0001^#^
Other hepatitis	5.84	2.79	−52.23^*^	2.97	2.57	−13.47^*^	6.70	3.12	−53.43^*^	< 0.0001^#^
Other Communicable, maternal, neonatal, and nutritional diseases	24.70	25.10	1.62^*^	10.30	10.62	3.11^*^	25.53	22.83	−10.58^*^	< 0.0001^#^
Other cancer	470.29	388.74	−17.34	379.87	319.99	−15.76^*^	462.12	410.32	−11.21	< 0.0001^#^
Other cardiovascular disease	84.91	60.18	−29.12^*^	51.47	39.97	−22.34^*^	82.94	60.89	−26.59^*^	< 0.0001^#^
Other chronic respiratory disease	18.93	26.83	41.73^*^	7.73	12.62	63.26^*^	17.56	25.06	42.71^*^	< 0.0001^#^
Oother Cirrhosis	434.85	248.48	−42.86^*^	227.64	134.52	−40.91^*^	455.10	273.65	−39.87^*^	< 0.0001^#^
Other digestive disease	240.54	193.04	−19.75^*^	122.43	105.32	−13.98^*^	231.21	200.34	−13.35^*^	< 0.0001^#^
Other Neurological disorders	128.95	111.54	−13.50^*^	109.35	94.14	−13.91^*^	126.99	111.71	−12.03^*^	< 0.0001^#^
Other mental and substance use disorders	981.56	728.01	−25.83^*^	477.09	403.61	−15.40^*^	956.63	740.48	−22.59^*^	< 0.0001^#^
Other diabetes, urogenital, blood, and endocrine diseases	176.05	140.34	−20.28^*^	138.54	128.90	−6.96	170.67	142.38	−16.58^*^	0.007^#^
Other Musculoskeletal disorders	421.90	361.62	−14.29	289.38	262.56	−9.27^*^	352.41	326.18	−7.44	< 0.0001^#^
Others	3.97	5.89	48.36^*^	98.84	116.44	17.81	3.49	4.87	39.54^*^	< 0.0001^#^
Other unintentional injury	377.97	264.52	−30.02^*^	238.41	176.36	−26.03^*^	331.06	239.31	−27.71^*^	< 0.0001^#^
Other injury	15.32	6.18	−59.66^*^	8.33	3.96	−52.46^*^	13.68	5.88	−57.02^*^	< 0.0001^#^

^*^Statistical significance using the test of Cochran-Armitage trend for year 2005 to 2015 comparison (*P* < 0.05); ^#^statistical significance using Poisson regression model for comparison of different culture regions (*P* < 0.05)

The trends of change of the leading cause of DALY rates in males and females in the three culture regions were similar to those of the whole study population (E-Figure 3-4).

### Region-specific life expectancy and HALE

Compared with Hakka and Hoklo regions, the life expectancy at birth was highest in Canton region in both 2005 (74.7 years) and 2015 (76.3 years). The life expectancy increased in both males (72.8 years vs. 74.7 years) and females (76.8 years vs. 78.2 years) from 2005 to 2015 in Canton region. Also, the HALE increased from 69.0 years to 69.6 years, with increases from 67.4 to 68.3 years among males and from 70.7 to 71.1 years among females between 2005 and 2015. The increase of life expectancy and HALE in Hakka and Hoklo regions was, however, greater than that in Canton region (Table 3).

**Table 3:** Region- and sex-specific life expectancy and healthy life expectancy at birth in 2005 and 2015

Year	Sex	Parameter	Region		
			Hakka	Canton	Hoklo
2005	All	life expectancy	72.00	74.74	71.79
		healthy life expectancy	64.10	68.95	63.64
	Male	life expectancy	69.49	72.83	69.30
		healthy life expectancy	62.00	67.35	61.64
	Female	life expectancy	74.89	76.79	74.62
		healthy life expectancy	66.55	70.71	65.93
2015	All	life expectancy	74.24	76.34	74.38
		healthy life expectancy	65.21	69.60	65.58
	Male	life expectancy	71.96	74.65	72.17
		healthy life expectancy	63.23	68.29	63.71
	Female	life expectancy	77.05	78.22	76.97
		healthy life expectancy	67.65	71.09	67.78

From 2005 to 2015, the gap between the life expectancy at birth and HALE increased from 7.9 years to 9.0 years in Hakka region, from 5.8 to 6.7 years in Canton region, and from 8.2 to 8.8 years in Hoklo region. Similar increase was noted in males and females between 2005 and 2015 in all cultural regions. In addition, the gap between the life expectancy at birth and HALE was higher in females than in males in all culture regions (Table 3).

## Discussion

### General findings

We have demonstrated the age-standardized mortality, the rate of DALY, and HALE in Hakka, Canton and Hoklo cultural regions. The different burden of disease might have stemmed from the different living habits, cultural heritage and socioeconomic status.

Key findings included: 1) There was a dominant but progressively decreased burden of non-communicable diseases; 2) There was regional and temporal variation in the age-standardized mortality and DALY rates for the top causes of death and certain diseases (including tuberculosis, cerebrovascular diseases, chronic respiratory diseases, depressive disorder, injuries, diarrhea and certain cancers); 3) Some causes (including cerebrovascular diseases, ischemic heart disease, chronic obstructive pulmonary disease, lung cancer and road injuries) were both in the top ten list of mortality and DALYS, while sensory organ diseases, neck and low back pain, congenital anomalies, neonatal disorders, and other mental and substance use disorders were not in the top ten list of mortality; 4) The mortality and DALY rates of colorectal cancer, lower respiratory infections, tuberculosis, Parkinson’s disease and Alzheimer’s disease ranked higher in the three cultural regions; 5) The life expectancy and HALE at birth were highest in Canton in both 2005 and 2015. Additionally, we noted greater gaps between the life expectancy at birth and HALE in the three cultural regions in 2015 than in 2005.

### Interpretation of findings

Canton, Hoklo and Hakka are three main cultural regions, with nearly half of the population in Guangdong province. The lowest mortality and DALY rates with the highest life expectancy and HALE were observed in Canton region, followed by Hoklo and Hakka regions. Canton is the coastal region with developed metropolis such as Guangzhou and Foshan where economic reforms initially took place, whereas Hakka is the mountainous region and Hoklo is another coastal region with significantly less developed cities and counties.

The three cultural regions shared the same top five leading causes of death including cerebrovascular disease, ischemic heart disease, COPD, lung cancer and liver cancer as the whole country [12]. In addition, we noted a substantial contribution of Alzheimer’s disease, colorectal cancer, nasopharyngeal cancer and lower respiratory disease in these cultural regions, raising the importance of disease control and prevention in Guangdong.

### Communicable, maternal, neonatal, and nutritional diseases

Previous studies have reported the experience in disease control and prevention in Canton region and part of Hoklo region [27–35], including comprehensive surveillance network, universal immunization, establishment of the rapid response and a preparedness team, closure of the live poultry market and intensified government control for Dengue fever. These efforts might have collectively contributed to the major reduction in morbidity of infectious diseases. Similar to the pattern of change in all-cause mortality, the lowest mortality and DALY of CMNN were noted in Canton region, followed by Hoklo and Hakka regions. We noted the lowest burden of diarrhea, intestinal and lower respiratory infectious diseases in Canton region, which might due to better sanitary conditions than in the other regions. In addition, some epidemiological studies showed that the population in Hoklo region has developed special habits of ingestion of raw salted shellfish, oyster and fish. These might have led to the high DALY and mortality rates of diarrhea and intestinal infectious diseases due to the insufficient heating during preparation [36, 37].

### Cardiovascular diseases

Cardiovascular disease remained the leading cause of mortality and DALY (lowest in Canton, followed by Hoklo, and Hakka regions). Similar to the 2013 China GBD study [12], cerebrovascular disease mortality remains the leading cause of premature mortality in China including Guangdong province. Notably, in 2015 cerebrovascular disease was the top leading cause of death in both Hoklo and Hakka regions, and was the top second in Canton region. Hypertension, alcohol drinking, smoking, westernization of dietary patterns and physical inactivity, which led to high systolic blood pressure, total cholesterol, and fasting plasma glucose, are the main determinants of the high cerebrovascular disease burden in China [38–40]. Based on the national non-communicable disease and nutrition survey in different disease surveillance points, the rate of alcohol drinking, smoking, sodium and oil consumption was lowest in Canton, followed by Hoklo and Hakka regions. This could partially explain the difference in mortality and DALY patterns of cerebrovascular disease [41, 42]. A systematic review suggested that hypertension wais the main determinant of the high cerebrovascular disease burden in China [39]. Treatment for hypertension is therefore a key strategy for controlling cerebrovascular disease [43]. The difference among the three cultural regions in the mortality of hypertensive heart diseases was not statistically significant, which was frequently related to the mortality of cerebrovascular diseases. The negative correlation with the income per capita (which was associated with better knowledge and awareness among the residents) might help partially explain for the regional differences in cerebrovascular disease mortality.

### Cancer

Cancer mortality and DALYs rates decreased in the three cultural regions. The better health care condition and treatment accessibility could help explain the lower mortality and DALY of liver, esophageal, stomach and tracheal, bronchial and lung cancer in Canton region. Moreover, our findings are not simply a function of socioeconomic development. The decreased burden of cancer might be attributed to the effective strategies and measures. Primary prevention with smoking cessation and alcohol drinking reduction campaigns could be related to the changes in lung, oesophageal, liver, and stomach cancer prevalence [44, 45]. Hepatitis B virus and hepatitis C virus have been well recognized as the predisposing factor to liver cancer. Novel effective treatments for hepatitis C have been introduced for reducing liver cancer mortality in China [46, 47], which might contribute to a further decrease in the disease burden in the future. The extensive coverage of hepatitis B vaccination has contributed to the reduced liver cancer mortality and DALY [12]. Screening and eradication of *Helicobacter pylori* infection is recommended in China for the susceptible population (who are at risk of having gastric cancer) [48], which might contribute to the decreased mortality of gastric cancer. Given that the sum of cervical and uterine cancer mortality remained rather constant between 2005 and 2015, the changes in death certification and/or the cause of death coding practices could have led to the increased mortality from cervical cancer, which was accompanied by a decrease in uterine cancer mortality.

In addition, some regional factors could not be neglected when taking the substantial burden of cancer into consideration. The high prevalence of Epstein-Barr virus infection could have consistently accounted for nasopharyngeal cancer [49] in the three cultural regions. In particular, we noted a higher disease burden of nasopharyngeal, esophageal and stomach cancer in Hoklo region than in Canton region. This could be related to the preference for liquor drinking, hot tea drinking, and fermented fish sauce ingestion in Hoklo region [50–53]. According to previous surveillance reports, the prevalence of esophageal cancer was relatively high in Hoklo and Hakka regions and Nan-an county (Fujian province). In our study, the mortality and DALYs of nasopharyngeal, esophageal and stomach cancer were highest in Hakka region, which has been neglected for years. The risk factors (high consumption of pickled vegetables, processed meat and spirits, and a family history) might have collectively contributed to the high prevalence and mortality in Hakka region [54]. However, the mortality and DALYs of cervical, colorectal and pancreatic cancer consistently increased in all three cultural regions, calling for continuous surveillance and effective intervention to reduce the disease burden.

### Other NCDs

Increased DALYs could be observed in some NCDs including sensory organ diseases, neck and low back pain, congenital anomalies, neonatal disorders and other mental and substance use disorders in the three cultural region. Likewise, DALYs of these diseases in Canton region were lower than in the other regions, highlighting the finding that higher socioeconomic status may lead to a greater input and convenient accessibility to health care resources. Despite the progress in increasing the life expectancy that contributed to the reduction of YLLs, there remain some issues related to the mitigation of the risks of YLDs which only occurred in some NCDs including sensory organ diseases, neck and low back pain, congenital anomalies, neonatal disorders. There is also a need to focus on shortening the duration of the functional health loss among the ageing population in different cultural regions with different population, environmental and socioeconomic characteristics. The persisting high incidence and prevalence highlights the importance of our findings, both in terms of the amelioration of suffering and the reduction of health-care costs. More emphasis should be paid to the prevention of disabling illnesses and mitigation of the adverse effects.

### Injuries

There was a remarkable reduction in transport injury, unintentional injury and intentional injury. Similar to previous studies, the mortality of transport and unintentional injury in more developed regions (Canton) was lower than in the less developed regions (Hakka and Hoklo) [12, 55, 56]. Effective and low-cost strategies including comprehensive education, seat belts and helmet use for motor cyclists, traffic separation, traffic calming and drunk driving interventions, and legislation coverage for injury prevention have been implemented in Canton region, which could have provided substantial benefits for the lower transport and unintentional injury burden [57–59].

### Life expectancy and HALE

Overall, our findings mirrored the findings of the 2016 GBD study, which reported that countries with lower socio-demographic Index (SDI) had higher mortality and lower life expectancy than those with higher SDI [1, 25]. Because the age-standardized mortality is decreasing whereas the life expectancy is increasing globally, it is important to estimate the quality of the years of life gained because this may offer the basis for planning health care policy to all citizens. Population in Canton region could have a greater access to health care and other non-health-specific interventions including poverty alleviation, education and family planning [24]. An increase in the total years of functional health lost (the life expectancy minus HALE) might indicate a reduction in mortality or a rise in morbidity. Furthermore, there was a greater reduction in mortality and progressive reduction in morbidity that resulted in a greater increase in the life expectancy and a less prominent increase in HALE.

### Limitations and strengths

There are notable limitations of our study. First, the estimates remained conservative because of the limited covariates included in the model specification and model parameter estimation, although the incompleteness was also calculated. Second, the quality of certification and coding as assessed through the fraction of garbage codes varied substantially across the regions (highest in Hoklo region, followed by Hakka and Canton regions). This indicated the imbalanced quality of death registration in each region. Third, there existed considerable differences for the estimates of certain types of cancer from different sources of data. Fourth, the relationship between DALY, HALE, mortality and culture region cannot be used for causal inference. Fifth, the YLDs were calculated using different methods based on diseases leading to both death and disability, or only leading to disability because data sources of prevalence and disability weights in China are still lacking. This could not comprehensively reflect the local conditions. Finally, the national census population in 2000 was used as the reference population to calculate the age-standardized mortality and DALY rates in this study, which hinder comparability with other regions and countries.

Nonetheless, our study findings remained robust. Between 2005 and 2015, the data quality in the three cultural regions has been improved significantly through the following efforts: systematic training of health care staffs, better cooperation among different governmental departments, better death report and surveillance, and the redistribution of garbage codes that followed the methods proposed by the WHO. Although the magnitude of quality improvement was different among the three cultural regions, the mortality, DALY, life expectancy and HALE approximated to the true estimates to the regional differences.

### Public health implications

In light of the greater life span, the postponement of the retirement age has led to multiple health issues including sensory organ diseases, and neck and low back pain. In addition, a lack of progress in reducing the DALYs, accompanied by congenital anomalies, neonatal disorders, neoplasm and cardiovascular diseases, necessitates a careful planning by the governments and health-care providers to ensure an adequate funding and staffs for disease prevention, treatment and rehabilitation services. Despite more spending on health in Canton region, the expenditure remains insufficient, calling for consolidated funding to support additional health care services. In addition, the remarkable burden of mental and substance use disorders, highlighting the importance of considering psychosocial problems which are readily overlooked.

Canton region has achieved greater success in reducing the disease mortality and DALYs. This could reflect the establishment of rapid response and a preparedness team, intensified government control of infectious diseases and comprehensive interventions for NCD control and prevention. Greater efforts should be endeavored to improve the primary prevention through avoidance of the known risk factors and sustained surveillance, and secondary prevention through early detection, treatment and affordable screening for precancerous lesions [60]. However, intervention to reduce the burden of local high-mortality diseases (i.e., diarrhea, infectious diseases, nasopharyngeal cancer) in Hoklo and Hakka regions is warranted in light of the unique environmental and cultural factors (e.g. eating habit). Collectively, understanding the regional discrepancies and similarities in disease burden may help provide not only interventions at the individual level, but also policy development at the governmental level.

## Conclusion

We have revealed the burden of disease in three main cultural regions during a 10-year period in Guangdong province. Understanding the environmental and cultural risk factors for local diseases might provide insights into the future public health measures to lower the disease burden.

## Supplementary information

**Additional file 1.** Supplementary e-Appendix.

## Data Availability

The datasets generated and/or analyzed during the current study are not publicly available but are available from the corresponding author on reasonable request.

## Abbreviations

CMNN: Communicable, maternal, neonatal, and nutritional diseases
DALY: Disability-adjusted life-years
HALE: Healthy life expectancy
HBV: Hepatitis B virus
HCV: Hepatitis C virus
NCD: Non-communicable disease
SDI: Socio-demographic Index
YLD: Years-of-life lived with disability
YLL: Years-of-life-lost

## References

[1] GBD 2016 DALYs and HALE Collaborators (2017). Global, regional, and national disability-adjusted life-years (DALYs) for 333 diseases and injuries and healthy life expectancy (HALE) for 195 countries and territories, 1990–2016: a systematic analysis for the Global Burden of Disease Study 2016. Lancet.

[2] Murray CJ (1994). Quantifying the burden of disease: the technical basis for disability adjusted life years. Bull World Health Organ.

[3] GBD 2016 Mortality Collaborators (2017). Global, regional, and national under 5 mortality, adult mortality, age-specific mortality, and life expectancy, 1970–2016: a systematic analysis for the Global Burden of Disease Study 2016. Lancet.

[4] GBD 2016 Disease and Injury Incidence and Prevalence Collaborators (2017). Global, regional, and national incidence, prevalence, and years lived with disability for 328 diseases and injuries for 195 countries, 1990–2016: a systematic analysis for the Global Burden of Disease Study 2016. Lancet.

[5] Mathers CD, Sadana R, Salomon JA, Murray CJ, Lopez AD (2001). Healthy life expectancy in 191 countries, 1999. Lancet.

[6] Murray CJ, Vos T, Lozano R (2012). Disability-adjusted life years (DALYs) for 291 diseases and injuries in 21 regions, 1990-2010: a systematic analysis for the global burden of disease study 2010. Lancet.

[7] Murray CJ, Barber RM, Foreman KJ (2015). Global, regional, and national disabilityadjusted life years (DALYs) for 306 diseases and injuries and healthy life expectancy (HALE) for 188 countries, 1990-2013: quantifying the epidemiological transition. Lancet.

[8] Kassebaum NJ, Arora M, Barber RM (2016). Global, regional, and national disabilityadjusted life-years (DALYs) for 315 diseases and injuries and healthy life expectancy (HALE), 1990-2015: a systematic analysis for the global burden of disease study 2015. Lancet.

[9] Mathers CD, Loncar D (2006). Projections of global mortality and burden of disease from 2002 to 2030. PLoS Med.

[10] WHO methods and data sources for life tables 1990–2016. http://www.who.int/healthinfo/statistics/LT_method.pdf?ua=1. Last accessed: 7 Dec 2019.

[11] WHO methods and data sources for global causes of death 2000–2011. http://www.who.int/healthinfo/statistics/GHE_TR2013-3_COD_MethodsFinal.pdf?ua=1. Last accessed: 7 Dec 2019.

[12] Zhou M, Wang H, Zhu J, Chen W, Wang L, Liu S (2016). Cause-specific mortality for 240 causes in China during 1990-2013: a systematic subnational analysis for the Global Burden of Disease Study 2013. Lancet.

[13] Yu W. National Disease Surveillance System monitoring causes of death 2010. 1st ed. Beijing: Military Medical Science Press; 2012.

[14] Cooke D, Leon JA (1976). Stability of population growth determined by 2 X 2 Leslie matrix with density-dependent elements. Biometrics.

[15] GBD 2016 Causes of Death Collaborators (2017). Global, regional and national age-sex specific mortality for 264 causes of death, 1980–2016: a systematic analysis for the Global Burden of Disease Study 2016. Lancet.

[16] Mathers CDD, Ma Fat M, Inoue C, Rao P, Lopez AD (2005). Counting the dead and what they died from: an assessment of the global status of cause of death data. Bull World Health Organ.

[17] Murray CJL, Lopez AD, Murray CJL, Lopez AD (1996). Alternative visions of the future: projecting mortality and disability, 1990–2020. The global burden of disease.

[18] Murray CJL, Lopez AD (1996). The global burden of disease: a comprehensive assessment of mortality and disability from diseases, injuries and risk factors in 1990 and projected to 2020.

[19] Peto R, Lopez AD, Boreham J, Thun M, Heath JC (1992). Mortality from tobacco in developed countries: indirect estimation from National Vital Statistics. Lancet.

[20] Mathers CD, Lopez AD, Murray CJL, Ezzati M, Jamison DT (2006). The burden of disease and mortality by condition: data, methods and results for 2001. Global burden of disease and risk factors.

[21] Lozano R, Naghavi M, Foreman K (2012). Global and regional mortality from 235 causes of death for 20 age groups in 1990 and 2010: a systematic analysis for the global burden of disease study 2010. Lancet.

[22] Murray CJ, Ezzati M, Flaxman AD (2012). GBD 2010: design, definitions, and metrics. Lancet..

[23] Murray CJL, Lopez (1994). Global Comparative assessment in the health sector: Quantifying disability: data, methods and results.

[24] Sullivan DF (1971). A single index of mortality and morbidity. HSMHA Health Rep.

[25] GBD 2017 DALYs and HALE Collaborators (2018). Global, regional, and national disability-adjusted life-years (DALYs) for 359 diseases and injuries and healthy life expectancy (HALE) for 195 countries and territories, 1990–2017: a systematic analysis for the Global Burden of Disease Study 2017. Lancet.

[26] Gakidou E, Cowling K, Lozano R, Murray CJ (2010). Increased educational attainment and its effect on child mortality in 175 countries between 1970 and 2009: a systematic analysis. Lancet.

[27] Fu C, Wang S (2016). Nosocomial infection control in healthcare settings: Protection against emerging infectious diseases. Infect Dis Poverty.

[28] Lam NS, Long X, Su XZ, Lu F (2018). Artemisinin and its derivatives in treating helminthic infections beyond schistosomiasis. Pharmacol Res.

[29] Jing QL, Cheng Q, Marshall JM, Hu WB, Yang ZC, Lu JH (2018). Imported cases and minimum temperature drive dengue transmission in Guangzhou, China: evidence from ARIMAX model. Epidemiol Infect.

[30] Huang X, Huang Q, Dun Z, Huang W, Wu S, Liang J (2016). Nontyphoidal Salmonella infection, Guangdong Province, China, 2012. Emerg Infect Dis.

[31] Huang DC, Wang JF (2018). Monitoring hand, foot and mouth disease by combining search engine query data and meteorological factors. Sci Total Environ.

[32] Zhong N (2004). Management and prevention of SARS in China. Philos Trans R Soc Lond Ser B Biol Sci.

[33] Wu J, Lu J, Faria NR, Zeng X, Song Y, Zou L (2016). Effect of live poultry market interventions on influenza a(H7N9) virus, Guangdong, China. Emerg Infect Dis.

[34] Liu T, Zhu G, He J, Song T, Zhang M, Lin H (2017). Early rigorous control interventions can largely reduce dengue outbreak magnitude: experience from Chaozhou, China. BMC Public Health.

[35] Guo P, Liu T, Zhang Q, Wang L, Xiao J, Zhang Q (2017). Developing a dengue forecast model using machine learning: a case study in China. PLoS Negl Trop Dis.

[36] Lunestad BT, Maage A, Roiha IS, Myrmel M, Svanevik CS, Duinker A (2016). An outbreak of Norovirus infection from shellfish soup due to unforeseen insufficient heating during preparation. Food Environ Virol.

[37] Alfano-Sobsey E, Sweat D, Hall A, Breedlove F, Rodriguez R, Greene S (2012). Norovirus outbreak associated with undercooked oysters and secondary household transmission. Epidemiol Infect.

[38] He J, Klag MJ, Wu Z, Whelton PK (1995). Stroke in the People’s republic of China. II. Meta-analysis of hypertension and risk of stroke. Stroke.

[39] Yong H, Foody J, Linong J (2013). A systematic literature review of risk factors for stroke in China. Cardiol Rev.

[40] Kyu HH, Bachman VF, Alexander LT, Mumford JE, Afshin A, Estep K (2016). Physical activity and risk of breast cancer, colon cancer, diabetes, ischemic heart disease, and ischemic stroke events: systematic review and dose-response meta-analysis for the global burden of disease study 2013. BMJ.

[41] Su X, Rong T, Liu Q, Zhao J, Long H, Wen Z (2005). Analysis of the smoking status in residents in Guangzhou. Zhong Guo Zhong Liu.

[42] Pan B, Du L, Luo B, Liu W, Chen J, Wang J (2008). Smoking behavior and risk factors in residents in Guangzhou. Zhong Guo Man Bing Yu Fang Kong Zhi.

[43] Aiyagari V, Gorelick PB (2009). Management of blood pressure for acute and recurrent. Stroke.

[44] Chan SSC, Cheung YTD, Wong DCN, Jiang CQ, He Y, Yang L, et al. Promoting smoking cessation in China: a foot-in-the-door approach to tobacco control advocacy. Glob Health Promot. 2017. 10.1177/1757975917720799. 10.1177/1757975917720799 28853637

[45] Yong J, Lin D, Tan XR (2017). Primary prevention of cardiovascular disease in older adults in China. World J Clin Cases.

[46] Ferenci P, Bernstein D, Lalezari J (2014). ABT-450/r–ombitasvir and dasabuvir with or without ribavirin for HCV. N Engl J Med.

[47] Gaetano J (2014). Benefit-risk assessment of new and emerging treatments for hepatitis C: focus on simeprevir and sofosbuvir. Drug Healthc Patient Saf.

[48] Shen L, Shan YS, Hu HM (2013). Management of gastric cancer in Asia: resource-stratified guidelines. Lancet Oncol.

[49] Ho CS (2017). Beating 'Guangdong cancer': a review and update on nasopharyngeal cancer. Hong Kong Med J.

[50] Tan HZ, Lin WJ, Huang JQ, Dai M, Fu JH, Huang QH (2017). Updated incidence rates and risk factors of esophageal cancer in Nan'ao island, a coastal high-risk area in southern China. Dis Esophagus.

[51] Tang WR, Chen ZJ, Lin K, Su M, Au WW (2015). Development of esophageal cancer in Chaoshan region, China: association with environmental, genetic and cultural factors. Int J Hyg Environ Health.

[52] Zhang DH, Chen JY, Hong CQ, Yi DQ, Wang F, Cui W (2014). High-risk human papillomavirus infection associated with telomere elongation in patients with esophageal squamous cell carcinoma with poor prognosis. Cancer.

[53] Ke L, Yu P, Zhang ZX (2002). Novel epidemiologic evidence for the association between fermented fish sauce and esophageal cancer in South China. Int J Cancer.

[54] Huang G, Zheng Z, Liu Q, Liu J (2000). Risk factors for esophageal cancer in residents living in Hakka region: a case-control study. Guang Dong Yi Xue.

[55] Dong X, Peek-Asa C, Yang J, Wang S, Chen X, Chi G (2011). The association of road safety knowledge and risk behaviour with paediatric road traffic injury in Guangzhou, China. Inj Prev.

[56] Wang SY, Li YH, Chi GB (2008). Injury-related fatalities in China: an under-recognised public-health problem. Lancet.

[57] Stevenson M, Yu J, Hendrie D, Li LP, Ivers R, Zhou Y (2008). Reducing the burden of road traffic injury: translating high-income country interventions to middle-income and low-income countries. Inj Prev.

[58] Li L, Scherpbier R, Wu J, Zhu X, Zhang W, Zhang L (2015). Legislation coverage for child injury prevention in China. Bull World Health Organ.

[59] Shi X, Wang T, Nie C, Wang H, Luo L, Qi Y, Jiang Z (2018). Epidemiologic features and intervention effect of fall injury among rural school-aged children in Southwest China: a short-term cohort study. Int J Inj Control Saf Promot.

[60] Lertkhachonsuk AA, Yip CH, Khuhaprema T, Chen DS, Plummer M, Jee SH (2013). Cancer prevention in Asia: resource-stratified guidelines from the Asian oncology summit 2013. Lancet Oncol.

