# Supplementary material for: Age-standardized mortality, disability-adjusted life-years and healthy life expectancy in different cultural regions of Guangdong, China: a population-based study of 2005–2015
Source: BMC Public Health. 2020 Jun 5;20:858. doi: 10.1186/s12889-020-8420-7 (PMC7275520; doi:10.1186/s12889-020-8420-7)
Supplement: Supplementary file 1 — Additional file 1. Supplementary e-Appendix. [file 12889_2020_8420_MOESM1_ESM.docx]

**Supplementary e-Appendix**

**Age-standardized mortality, disability-adjusted life-years and healthy life expectancy in different cultural regions of Guangdong, China: A population-based study of 2005-2015**

Xue-yan Zheng^1^, Xiao-jun Xu^1^, Yi-yang Liu^1,2^, Yan-jun Xu^1^, Si-xing Pan^1,2^, Xin-ying Zeng^3^, Qian Yi^1,2^, Ni Xiao^1^, Li-feng Lin^1*^

1 Institute of Non-communicable disease control and prevention, Guangdong provincial center for disease control and prevention, Guangdong, China.

2 Department of Epidemiology and Biostatistics, School of Public Health, Guangdong Pharmaceutical University, Guangzhou, China.

1. National Center for Chronic and Non-Communicable Disease Control and Prevention, Chinese Center for Disease Control and Prevention, Beijing, China.

*** Correspondence author:** **Li-feng Lin.** Institute of Non-communicable disease control and prevention, Guangdong provincial center for disease control and prevention, Guangdong, China. Address: 160 Qunxian Road, Panyu district, Guangzhou, Guangdong, China. Fax: 020-31051821, Phone: 020-31051821, E-mail: [1396320174 @qq.com](mailto:17491430@qq.com).

Index

**Methods.......................................................2**

**Results..........................................................41**

**Discussion..........................................................76**

**Methods**

**
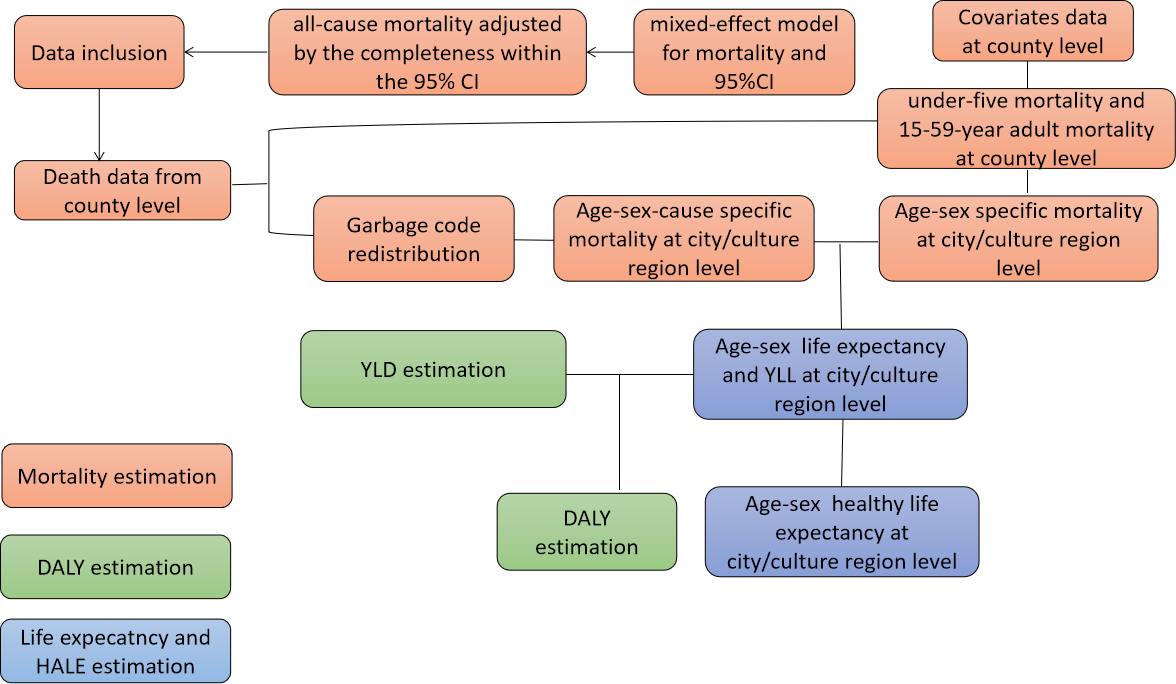
**

1. **Figure 1 Overview flow chart of age-standardized mortality, disability-adjusted life-years and healthy life expectancy in different cultural regions of Guangdong, China**

**Data source**

1. Death data source for the estimation of the all-cause and specific disease mortality, DALY, life expectancy and HALE: the main data source for mortality estimates was derived from the Disease Surveillance Points, the Chinese Center for Disease Control and Prevention Cause of Death Reporting System and under-reporting of death survey at county levels between 2005 and 2015 [1-3]. Death information was mandatory in the Disease Surveillance Points, the Chinese Center for Disease Control and Prevention Cause of Death Reporting System in China. However, owing to the incompleteness of the death rate, under-reporting of death survey at county levels was conducted between 2005 and 2015. Briefly, the county with an averaged level of mortality rate, population size and socioeconomic levels was selected to represent the city that the county belonged to. The death cases were surveyed through multiple routine measures, including the records from the residential [committee](C:/Program%20Files%20(x86)/Youdao/Dict/7.5.2.0/resultui/dict/javascript:;), [funeral](C:/Program%20Files%20(x86)/Youdao/Dict/7.5.2.0/resultui/dict/?keyword=funeral) home and police stations. To ascertain the degree of under-reporting, the information of death cases was further compared with the registered information from Chinese Center for Disease Control and Prevention Cause of Death Reporting System.
2. Covariate data source to estimate for the mortality of children aged under five years (under-five mortality, U5MR) and adults: covariates including the duration of education, gross domestic product (GDP), and the rate of urbanization were derived from the population census and National statistical yearbook between 2005 and 2015. Regarding the irregular [fluctuation](file:///D:\\Users\\nfernandez\\Program%20Files%20(x86)\\Youdao\\Dict\\7.5.0.0\\resultui\\dict\\)s, the [outlier](file:///D:\\Users\\nfernandez\\Program%20Files%20(x86)\\Youdao\\Dict\\7.5.0.0\\resultui\\dict\\)and [deletion](javascript:;) of the covariates at county levels, different models were employed for provision of the more appropriate estimation. Further details are described below.
3. Population data source was applied to analyze the age-standardized mortality with the direct standardization methods, adjusting for the population structure: the population at county level was derived from census from 2000 and 2010 using the Leslie model [4].

**Covariates for estimation**

Covariates (the duration of education, GDP, and the rate of urbanization) were included to estimate the mortality of children aged under five years (under-five mortality, U5MR) and adults. Regarding the irregular [fluctuation](file:///D:\\Users\\nfernandez\\Program%20Files%20(x86)\\Youdao\\Dict\\7.5.0.0\\resultui\\dict\\)s, the [outlier](file:///D:\\Users\\nfernandez\\Program%20Files%20(x86)\\Youdao\\Dict\\7.5.0.0\\resultui\\dict\\)and [deletion](javascript:;) of the covariates at county levels, different models were employed for the provision of the more appropriate estimation. The moving weighted average method was applied to estimate the lagged distributed income (LDI), which reflected the 5-year lag of the GDP. Logit model was employed to estimate the duration of education and the rate of urbanization [5-6]. The formulae are summarized as follows:

1. **Lagged Distributed Income (LDI)**: Moving weighted average method was applied to estimate the LDI, which reflected the 5-year lag of the GDP.

1. **Duration of education:** the fluctuation of the annual duration of education remained low, therefore the logit model was employed to estimate the annual duration of education based on the census data of 2000 and 2010.

1. **Urbanization rate:** Similar to the methods for estimating the duration of education, the logit model was employed to estimate the rate of urbanization based on the census data of 2000 and 2010.

$P\text{n}=\frac{\text{exp}\left(\text{n}\times\text{r}+\text{logit2000}\right)}{1+\text{exp}\left(\text{n}\times\text{r}+\text{logit2000}\right)}$, $\text{r}=\frac{\text{logit2010-logit2000}}{10}$

**Methods for estimation of the all-cause mortality at culture-region-level and city-level**

**
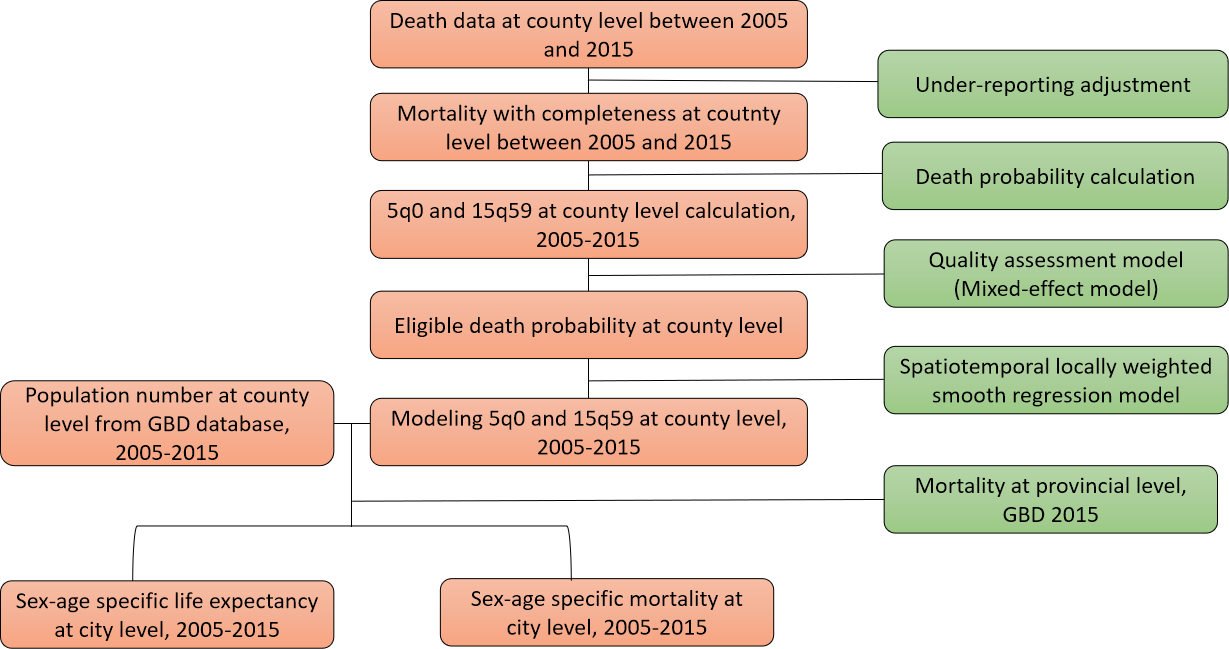
**

**E-Figure 2. Flow chart for estimation of the all-cause mortality at culture-region-level and city-level**

Data sources were analyzed for the county-level mortality because of the changes in administrative units. Further calculation of cultural-region mortality was conducted based on the county level. The method we have applied for cultural-region- and county-level all-cause mortality estimation included the following analytical or computational steps:

1. Because of the incompleteness of the reported data in each system, the rate of under-reporting was calculated for more accurate estimates of mortality. The rate of incompleteness was calculated based on the under-reporting of the death survey. Briefly, the county with a similar level of mortality rate, population size and social-economic levels was selected to represent the city that the county belonged to, for surveying the death cases through multiple routine measures (including the records from the residential [committee](file:///D:\\Users\\nfernandez\\Program%20Files%20(x86)\\Youdao\\Dict\\7.5.2.0\\resultui\\dict\\javascript:;), [funeral](file:///D:\\Users\\nfernandez\\Program%20Files%20(x86)\\Youdao\\Dict\\7.5.2.0\\resultui\\dict\\%3fkeyword=funeral) home and police stations). To ascertain the degree of under-reporting, the information of death cases was further subject to the comparison with all the registered information from Chinese Center for Disease Control and Prevention Cause of Death Reporting System.

Information collected in the death system and survey was summarized as follow:

|  |  | **Death information system** | | |
| --- | --- | --- | --- | --- |
| **Survey** |  | Yes | No | Total |
|  | Yes | C | N2 | C+N2 |
|  | No | N1 | Y=(N1*N2)/C | N1 + Y |
|  | Total | C + N1 | N2 + Y | N=C+N1+N2+Y |

$C\text{ompleteness}=\frac{C+N1}{N}$; $I\text{ncompleteness}=\frac{N2+Y}{N}$
 OR

*M*_real_: real mortality rate, *M*_reported_: reported mortality rate

1. Under-five mortality and 15-59-year adult mortality were calculated according to WHO’s life tables [2];
2. All-cause mortality and its 95% confidence interval (95%CI) at county levels were derived based on the mixed-effect model that included the socioeconomic variables of the lagged distributed income, the duration of education, urbanization rate, year, under-five mortality and 15-59-year adult mortality [7-11]. The quality of all-cause mortality adjusted by the completeness of the data was deemed eligible when falling within the 95%CI, or otherwise deemed ineligible for inclusion in the next step;

Log_q: logit model of death probability

1. The qualified mortality rates at county level were included to estimate the sex-specific under-five mortality and 15-59-year adult mortality for all counties based on the spatiotemporal [locally weighted smooth regression (S-T LOESS)](http://www.so.com/link?m=aWw%2FEsi9BOiI8vXjwSYP%2Fn1b9ib9Jbx%2BjpjsQaB3i1L6faYwbi7PbBSqdXlZSClqdForS3O4%2FXvQnbXWbl1Ct8RGDAmPjtucSdZ5mNp4aaK8ANvPDVlEiI6WneGQ%3D) model;

Under-five mortality calculation formula:

*Edu_i,t_*: the level of education of the mother in *i* county in *t* year; *LDI_i,t_*: lagged distributed income; *URB_i,t_*: urbanization rate; *γ_i,t_*_:_ random effect in *i* city in *t* year; ε_i,t_: random effect in *i* culture region in *t* year

Adult mortality formula:

*Edu_i,t_*: the level of education of the population in *i* county in *t* year; *LDI_i,t_*: lagged distributed income; *URB_i,t_*: urbanization rate; *γ_i,t_*_:_ random effect in *i* culture region level in *t* year; ; ε_i,t_: random effect in *i* city in *t* year.

5) An estimate of the age-specific mortality at county level was estimated by modeling the life tables from the summary measures of under-five mortality and 15-59-year adult mortality (5q0 and 59q15, respectively). Furthermore, these estimates were re-scaled to match the 2015 GBD provincial-level estimates. Finally, age-specific mortality at different age-groups at city and culture region levels would be obtained from the above-mentioned re-scaled county-level mortality.

6) The direct standardization method was adopted to adjust for the impact of the population structure. The census population in China in year 2000 was used as the reference population.

**Cause-specific mortality estimation**

**
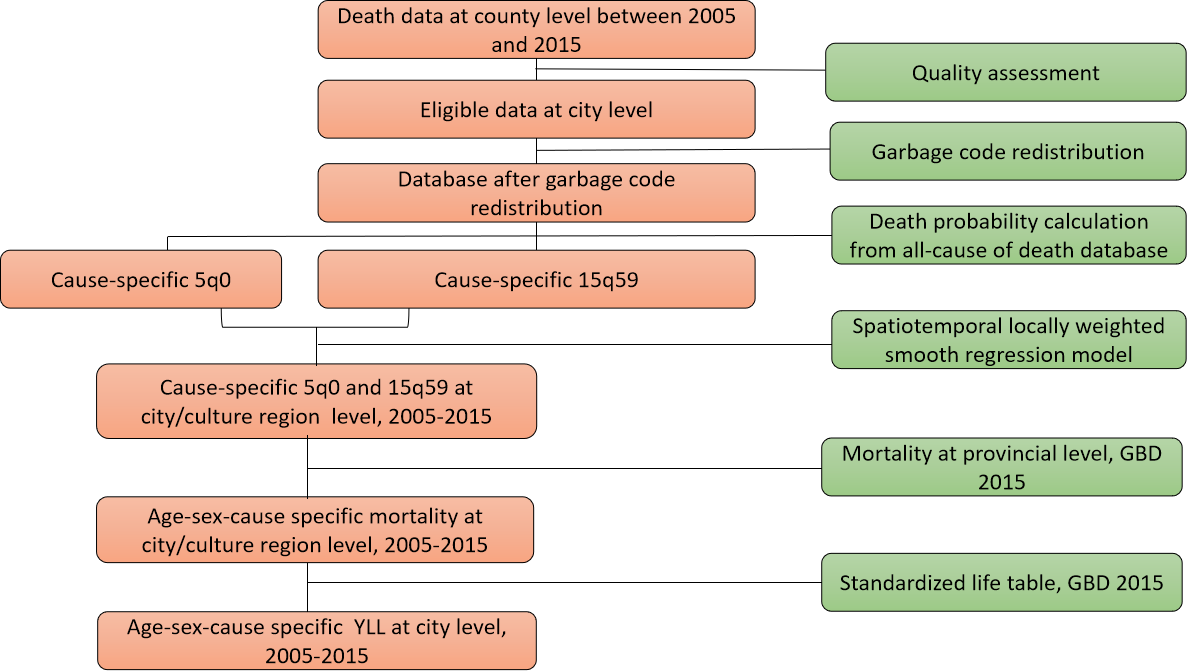
**

**E-Figure 3. Flow chart for estimation of the cause-specific mortality at culture-region-level and city-level**

We identified and re-distributed, by age and sex groups, the ICD-10 codes which cannot be assigned to the underlying causes of death, those that were intermediate causes of death rather than the underlying causes, or those that lacked specificity in coding. The proportion of deaths with the underlying cause coded to a short list of garbage codes was calculated and reassigned by using the previously published methods [2-3, 12]:

1. Symptoms, signs and ill-defined conditions (ICD10 codes R00‐R99);
2. Injuries undetermined whether intentional or unintentional (Y10-Y34, Y87.2);
3. Ill-defined cancers (C76, C80, and C97);
4. Ill-defined cardiovascular diseases (I47.2, I49.0, I46, I50, I51.4, I51.5, I51.6, I51.9 and I70.9).
5. **Redistribution of the garbage codes of cardiovascular death causes**

Ill-defined causes of cardiovascular death were re-distributed to ischemic heart disease and the group of other cardiovascular causes of death according to WHO by specific age and sex groups [2-3, 12]. The number of ischemic heart disease after garbage code re-distribution equaled the number of ischemic heart disease before garbage code re-distribution, plus the product of the number of cardiovascular disease garbage code and the coefficient from the table below.

**E-table 1. Coefficient of garbage code of cardiovascular disease redistribution for specific age and sex from WHO [3]**

|  | Male | Female |
| --- | --- | --- |
| <1 year | 0 | 0 |
| 1 to 4 | 0 | 0 |
| 5 to 9 | 0 | 0 |
| 10 to 14 | 0 | 0 |
| 15 to 19 | 0 | 0 |
| 20 to 24 | 0 | 0 |
| 25 to 29 | 0 | 0 |
| 30 to 34 | 0 | 0 |
| 35 to 39 | 0 | 0 |
| 40 to 44 | 0.11 | 0 |
| 45 to 49 | 0.04 | 0 |
| 50 to 54 | 0.04 | 0.1 |
| 55 to 59 | 0.20 | 0.14 |
| 60 to 64 | 0.16 | 0.12 |
| 65 to 69 | 0.25 | 0.25 |
| 70 to 74 | 0.26 | 0.20 |
| 75 to 79 | 0.23 | 0.17 |
| 80 to 84 | 0.03 | 0.06 |
| ≥85 | 0.03 | 0.06 |

1. **Cancer garbage code redistribution**

Except for the hepatic, pancreatic, ovarian and lung cancer, the cancer of other organs or systems with unspecified sites were re-distributed pro-rata to all sites. Additionally, we re-distributed cancer of the uterus, part unspecified (C55) pro-rata to cervix uteri (C53) and corpus uteri (C54).

1. The formula of the number of specific ill-defined cancers in the short list (Ns) after re-distribution:

Ns_after_ = Ns_before_* (1+Nt_gab_/Nt_before_)

Ns_after_: the number of specific ill-defined cancer deaths in the short list after re-distribution; Ns_before_: the number of specific ill-defined cancer deaths before re-distribution; Nt_gab_: the total number of garbage code cancer deaths; Nt_before_: the total number of cancer deaths before re-distribution.

1. The formula of number of other cancers (No) after redistribution:

No_after_ = (No_before_-Nt_gab_) * (1+Nt_gab_/Nt_before_)

No_after_: the number of other cancers after re-distribution; No_before_: the number of other cancers before re-distribution

1. **Redistribution of the injury garbage code and unspecified death causes**

Ill-defined injury codes (Y10-Y34, Y87.2) were re-distributed pro‐rata to specific death cause of injury. Additionally, we re-distributed the deaths coded to symptoms, signs and ill-defined conditions pro-rata to all non-injury causes of death. Here, we demonstrate an example of the ill-defined injury in a specific category:

M_after,inj_ = M_before,inj_ * (1+ M_gab,inj_/M_total,inj_)
M_after,inj_: the mortality rate of injury in specific category after re-distribution; M_before,inj_: the mortality rate of injury in specific category before re-distribution; M_gab,inj_: the mortality rate of ill-defined injury cause; M_total,inj_: the total mortality rate of injury

The fraction of deaths assigned to the major garbage codes varies extensively, even across the high-SDI (socio-demographic index) countries. Because of the potential bias, death data source at county level with more than 5% of the ICD code inaccuracy was excluded. Crude mortality greater than 3‰ at city levels would be included in the cause-specific mortality estimation, or otherwise re-calculated by using the spatiotemporal [locally weighted smooth regression (S-T LOESS)](http://www.so.com/link?m=aWw%2FEsi9BOiI8vXjwSYP%2Fn1b9ib9Jbx%2BjpjsQaB3i1L6faYwbi7PbBSqdXlZSClqdForS3O4%2FXvQnbXWbl1Ct8RGDAmPjtucSdZ5mNp4aaK8ANvPDVlEiI6WneGQ%3D) model that included the lagged distributed income, the duration of education, the rate of urbanization and the year as the covariates [7-15].

**Estimation of disability-adjusted life-years (DALYs)**

1. **DALYs**: We calculated the DALYs as the sum of YLLs and YLDs for each cause, location, age group, sex, and year [11, 16]. The census population in 2000 in China was used as the reference population to calculate the DALY with the direct standardization method, adjusting for the population structure.

DALY= YLL+YLD

1. **YLL:** The YLL basically corresponds to the number of deaths multiplied by the standard life expectancy at the age at which death occurs. The basic formula for YLL, is the following for a given cause, age and sex:
    YLL = N × L

N:  number of deaths for a given cause, age and sex

L: standard life expectancy at age of death in years, from GBD 2015 (E-Table 2).

**E-Table 2 Standardized life table**

| Age group | Life expectancy |
| --- | --- |
| 0- | 86.59408 |
| 1- | 85.76748 |
| 5- | 81.80519 |
| 10- | 76.83121 |
| 15- | 71.85669 |
| 20- | 66.90784 |
| 25- | 61.96704 |
| 30- | 57.02344 |
| 35- | 52.09721 |
| 40- | 47.19935 |
| 45- | 42.35229 |
| 50- | 37.58122 |
| 55- | 32.89429 |
| 60- | 28.28719 |
| 65- | 23.78644 |
| 70- | 19.41143 |
| 75- | 15.26225 |
| 80- | 11.45059 |
| 85- | 8.152726 |
| 90- | 5.526635 |
| 95- | 3.713400 |
| 100- | 2.500702 |
| 105- | 1.621429 |
| 110- | 1.372479 |

**YLD:**

**
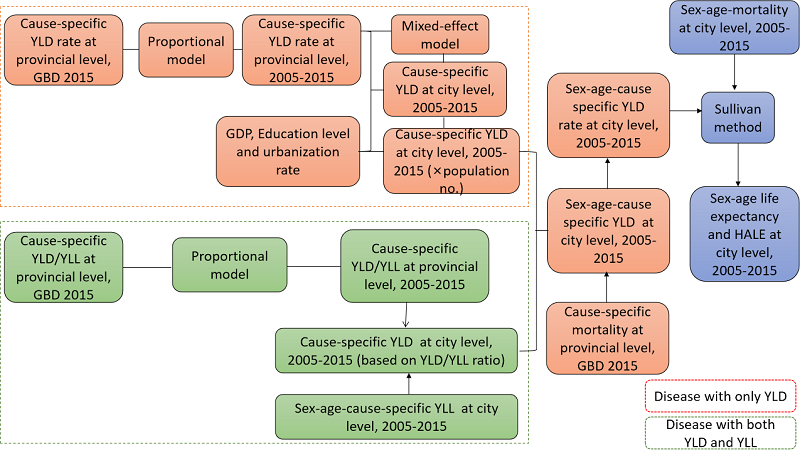
**

**E-Figure 4. Flow chart for estimation of the YLD and HALE at culture-region-level and city-level**

1. The YLL was to some extent proportional to the YLD in the diseases which could both lead to death and disability [17]. YLDs of the diseases both leading to death and disability was calculated based on the proportion for each cause, location, sex, age and year provided by GBD 2015.

1. YLDs of the diseases only leading to disability (but not death) were estimated based on the S-T LOESS model including the covariates of the lagged distributed income, the duration of education, the rate of urbanization and city-level and cultural region-level random effects. To ensure that the sum of YLDs in the lower category diseases equaled that of the upper category disease, the YLDs were re-scaled to match the 2015 GBD provincial-level death numbers. The cultural region level sex-specific YLD at different age-groups and city-level estimates were obtained.

*Edu_i,t_*: the level of education of the mother in *i* county in *t* year; *LDI5_i,t_*: lagged distributed income after 5 years; *URB_i,t_*: urbanization rate; *γ_i,t_*_:_ random effect in *i* culture region level in *t* year; ε_i,t_: random effect in *i* city in *t* year.

To ensure that the sum of YLDs in the lower level of the cause hierarchy equaled that of the upper level of the cause hierarchy, the YLDs were adjusted to match the provincial-level YLD numbers from GBD 2015 study. The cultural region-level sex-specific YLD at different age-groups and city-level estimates were obtained at each year.

**Estimation of life expectancy and healthy life expectancy (HALE)**

Final estimates of age-sex-specific mortality rates for years 2005-2015 were used to compute abridged life tables with the use of the methods developed by Sullivan et al. [16]. The same estimates of the YLDs per person for each location, age, sex, and year from 2005 to 2015 were used to establish the HALE by the age group within abridged multiple ­decrement life tables [18]. For constructing the life tables, we utilized the estimated age schedules of mortality and disability. The interval of the age group used in the life tables was 5 years for an age group, except for the 0-1 and 1-4 year age groups. In the life expectancy calculation, the common version of the Sullivan life table method was applied. In the HALE calculation, we aimed to take into account the relationships between disability and time to death. Annual age- sex-specific years of the life adjusted estimates of YLD rate were used as input to the life table. As the initial step, we stratified the life table population into subpopulations according to their age and sex at death (or the life span). The number of people in subpopulation with the life span X equaled the number who would die at the age of X according to the life table. Next, for each population with the same age at death, we estimated the sex-age-specific schedule of disability. Finally, we estimated the sex-age-specific YLD rates for the total life table population. The total life expectancy and the years lived with disability rate were calculated for the aggregated life table population.

Life expectancy and healthy life expectancy at birth were reported for the three cultural regions.

lx：No. of individuals surviving at the age of x in the life tables;

L_i_: Years lived in interval from the age of x to x+5;

w: The last age group;

YLD_ri_: YLD rate at different age group

**Other analyzes**

The direct standardization method was adopted to adjust for the impact of the population age-structure. The census population in year 2000 was used as the reference population. The test of Cochran-Armitage trend was used to examine the significance of trends in mortality. Poisson regression model was conducted to explore for the associations of deaths with the socio-demographic factors of year and cultural region.

**E-Table 3. ICD-10 codes for causes from different levels.**

| level | Cause | ICD-10 Code |
| --- | --- | --- |
| 1 | Communicable,maternal, neonatal, and nutritional diseases | A00-A00.9,A01.0-A14,A15-A28.9,A31,A31.0,A31.8-A39.9,A42-A44.9,A48.1,A48.2,A48.4-A48.52,A49.1,A50-A58,A63-A63.8,A65-A70,A74,A74.8-A75.9,A77-A96.9,A98-A98.8,B00-B06.9,B10-B10.89,B15-B17.2,B19.1-B19.21,B20-B27.99,B29.4,B33-B33.8,B37-B37.2,B37.5-B37.89,B38-B54.0,B55.0,B56-B60.8,B63,B65-B65.9,B67-B72.0,B74.3-B75,B77-B78.9,B80-B81.8,B83-B83.8,B90-B91,B94.1,B94.2,B95-B95.5,D50-D52.0,D52.8-D53.9,D62-D63.8,D64.1-D64.3,D64.8-D64.9,D69.9,E00-E02,E40-E46.9,E51-E61.9,E63-E64.0,E64.2-E64.9,F07.1,G00.0-G00.8,G03-G03.8,G04-G05.8,G14,G14.6,H70-H70.93,I00,I02,I02.9,J01-J01.91,J02.0,J03.0-J03.01,J05-J05.11,J09-J15.8,J16-J16.9,J20-J21.9,J36,J36.0,K67.0-K67.8,K74.7,K74.8,K93.0,M03.1,M12.1-M12.19,M49.0,M49.1,M73.0,M73.1,M89.6-M89.69,N70-N71.9,N73-N74.8,N96,N98-N98.9,O00-O07.9,O09-O16.9,O20-O26.93,O28-O36.93,O40-O48.1,O60-O77.9,O80-O92.79,O96-P03.1,P03.6-P05.9,P07-P15.9,P19-P29.9,P35-P39.9,P50-P61.9,P70,P70.3-P72.9,P74-P78.9,P80-P81.9,P83-P84,P90-P94.9,P96,P96.3,P96.4,P96.8-P96.89,R19.7 |
| 2 | HIV/AIDS and tuberculosis | A10-A14,A15-A19.9,B20-B24.9,B45-B45.9,B90-B90.9,K67.3,K93.0,M49.0,P37.0 |
| 3 | Tuberculosis | A10-A14,A15-A19.9,B90-B90.9,K67.3,K93.0,M49.0,P37.0 |
| 3 | HIV/AIDS | B20-B24.9,B45-B45.9 |
| 2 | Diarrhea, lower respiratory, and other common infectious diseases | A00-A00.9,A01.0-A09.9,A33-A37.91,A39-A39.9,A83-A87.9,B01-B02.9,B05-B05.9,B94.1,F07.1,G00.0-G00.8,G03-G03.8,G04-G05.8,H70-H70.93,J01-J01.91,J05-J05.11,J09-J15.8,J16-J16.9,J20-J21.9,J36,J36.0,P23-P23.9,P35.8,R19.7 |
| 3 | Diarrheal diseases | A00-A00.9,A02-A04.1,A04.3,A04.5-A07,A07.2-A07.4,A08-A09.9,R19.7 |
| 3 | Intestinal infectious diseases | A01.0-A01.4,A04.2,A04.4,A07.0,A07.1,A07.8,A07.9 |
| 3 | Lower respiratory infections | J09-J15.8,J16-J16.9,J20-J21.9,P23-P23.9 |
| 3 | Meningitis | A39-A39.9,A87-A87.9,G00.0-G00.8,G03-G03.8 |
| 3 | Measles | B05-B05.9 |
| 2 | Neglected tropical diseases and malaria | A68-A68.9,A69.2-A69.5,A75-A75.9,A77-A79.9,A82-A82.9,A90-A96.9,A98-A98.8,B50-B54.0,B55.0,B56-B58,B59-B60.8,B65-B65.9,B67-B72.0,B74.3-B75,B77-B78.9,B80-B81.8,B83-B83.8,P37.1,P37.3,P37.4 |
| 3 | Malaria | B50-B54.0,P37.3,P37.4 |
| 3 | Intestinal nematode infections | B77-B77.9 |
|  |  |  |
| 2 | Maternal disorders | N96,N98-N98.9,O00-O07.9,O09-O16.9,O20-O26.93,O28-O36.93,O40-O48.1,O60-O77.9,O80-O92.79,O96-O99.91 |
| 2 | Neonatal disorders | P00-P03.1,P03.6-P05.9,P07-P15.9,P19-P22.9,P24-P29.9,P36-P36.9,P38-P39.9,P50-P61.9,P70,P70.3- P72.9,P74-P78.9,P80-P81.9,P83-P84,P90-P94.9,P96,P96.3,P96.4,P96.8-P96.89 |
| 2 | Nutritional deficiencies | D50-D52.0,D52.8-D53.9,D62-D63.8,D64.1-D64.3,D64.8-D64.9,D69.9,E00-E02,E40-E46.9,E51-E61.9,E63- E64.0,E64.2-E64.9,M12.1-M12.19 |
| 3 | Iron-deficiency anemia | D50-D50.9,D62-D63.8,D64.1-D64.3,D64.8-D64.9,D69.9 |
| 2 | Other communicable, maternal, neonatal, and nutritional diseases | A20-A28.9,A31,A31.0,A31.8-A32.9,A38-A38.9,A42-A44.9,A48.1,A48.2,A48.4-A48.52,A49.1,A50-A58,A63-A63.8,A65-A67.9,A69-A69.1,A69.8-A70,A74,A74.8-A74.9,A80-A81.9,A88-A89.9,B00-B00.9,B03,B04,B06-B06.9,B10-B10.89,B15-B17.2,B19.1-B19.21,B25-B27.99,B29.4,B33-B33.8,B37-B37.2,B37.5-B37.89,B38-B44.9,B46-B49.9,B58.0-B58.9,B63,B91,B94.2,B95-B95.5,G14,G14.6,I00,I02,I02.9,J02.0,J03.0-J03.01,K67.0-K67.2,K67.8,K74.7,K74.8,M03.1,M49.1,M73.0,M73.1,M89.6-M89.69,N70-N71.9,N73-N74.8,P35-P35.3,P35.9,P37,P37.2,P37.5-P37.9 |
| 3 | Sexually transmitted diseases excluding HIV | A50-A58,A63-A63.8,B63,K67.0-K67.2,M03.1,M73.0,M73.1,N70-N71.9,N73-N74.8 |
| 3 | Hepatitis | B15-B17.2,B19.1-B19.21,B94.2,P35.3 |
| 4 | Acute hepatitis A | B15-B15.9 |
| 4 | Acute hepatitis B | B16-B17.0,B19.1-B19.11,P35.3 |
| 4 | Acute hepatitis C | B17.1-B17.11,B19.2-B19.21 |
| 1 | Non-communicable diseases | A31.1,A31.2,A46,A46.0,B18-B18.9,B37.3-B37.49,B37.9,C00-C13.9,C15-C25.9,C30-C34.92,C37-C38.8,C40-C41.9,C43-C45.9,C47-C54.9,C56-C57.8,C58,C58.0,C60-C63.8,C64-C67.9,C68.0-C68.8,C69-C75.8,C81-C86.6,C88-C97.9,D00.00-D00.2,D01.0-D01.3,D02.0-D02.3,D03-D06.9,D07.0-D07.2,D07.4,D07.5,D09.0,D09.2-D09.8,D10.0-D10.7,D11-D12.9,D13.0-D13.7,D14.0-D14.32,D15-D16.9,D22-D25.9,D26.0,D26.1,D27-D27.9,D28.0-D28.7,D29.0-D29.8,D30.0-D30.8,D31-D36,D36.1-D36.7,D37.01-D37.5,D38.0-D38.5,D39.1-D39.8,D40.0-D40.8,D41.0-D41.8,D42-D43.9,D44.0-D44.8,D45-D47.9,D48.0-D48.62,D49.2-D49.4,D49.6,D49.81,D52.1,D55-D58.9,D59.0-D59.3,D59.5,D59.6,D60-D61.9,D64.0,D64.4,D66-D69.8,D70-D75.89,D76-D78.89,D80-D83.9,D84.0-D84.8,D86-D86.9,D89-D89.3,E03-E07.1,E09-E16.9,E20-E34.8,E36-E36.8,E65-E68,E70-E85.29,E87.71,E88-E89.9,F00-F03.91,F06.2,F10-F11.99,F13-F16.99,F18-F23.9,F25-F29.9,F50.0-F50.1,G10-G13.8,G20-G26.0,G30-G31.9,G35-G37.9,G40-G41.9,G45-G46.8,G47.3-G47.39,G61-G61.9,G70-G73.7,G90-G90.9,G93.7,G95-G95.9,G97-G97.9,I01-I01.9,I02.0,I05-I09.9,I11-I13.9,I20-I25.9,I27.1,I28-I28.8,I30-I31.1,I31.8,I31.9,I33-I42.9,I47-I48.92,I51.0-I51.6,I60-I61.9,I62.0-I62.03,I63-I63.9,I65-I66.9,I67.0-I67.3,I67.5-I67.7,I69.0-I69.198,I69.20-I69.398,I70.2-I70.8,I71-I78.9,I80-I89.9,I91.9,I95.2,I95.3,I97-I98.2,I98.9,J30-J35.9,J37-J47.9,J60-J63.8,J65-J68.9,J70-J70.9,J82,J84-J84.9,J91-J92.9,J95-J95.9,K20-K22.9,K25-K29.91,K31-K31.89,K35-K38.9,K40-K46.9,K50-K52.9,K55-K57.93,K58.0-K62.9,K63.5,K64-K64.9,K66.8,K67,K68-K68.9,K70-K70.9,K71.3-K71.51,K71.7,K72.1-K74.69,K74.9,K75.2-K77.8,K80-K83.9,K85-K86.9,K90-K92,K92.8-K92.89,K94-K95.89,L00-L05.92,L08-L08.9,L10-L14.0,L51-L51.9,L88-L89.95,L93-L93.2,L97-L98.499,M00-M03.0,M03.2,M03.6,M05-M09.8,M30-M36.8,M40-M43.19,M65-M65.08,M71.0-M71.19,M86-M87.19,M88-M89.09,M89.5-M89.59,M89.7-M89.9,N00-N08.8,N10-N12.9,N14-N16.8,N18-N18.9,N20-N23.0,N25-N32.0,N32.3,N32.4,N34-N34.3,N36-N36.9,N39-N39.2,N41-N41.9,N44-N45.9,N49-N51.8,N65-N65.1,N72,N72.0,N75-N77.8,N80-N81.9,N83-N83.9,N84.0,N84.1,N87-N87.9,N99-N99.9,P03.2-P03.5,P70.0-P70.2,P96.0-P96.2,P96.5,Q00-Q07.9,Q10.4-Q18.9,Q20-Q28.9,Q30- Q36,Q37-Q45.9,Q50-Q87.89,Q89-Q89.8,Q90-Q93.9,Q95-Q95.9,Q97-Q97.9,Q99- Q99.8,R50.2,R50.82,R50.83,R73-R73.9,R78.0-R78.5,R95,X45-X45.9,X49-X49.9 |
| 2 | Neoplasms | C00-C13.9,C15-C25.9,C30-C34.92,C37-C38.8,C40-C41.9,C43-C45.9,C47-C54.9,C56-C57.8,C58,C58.0,C60-C63.8,C64-C67.9,C68.0-C68.8,C69-C75.8,C81-C86.6,C88-C97.9,D00.00-D00.2,D01.0-D01.3,D02.0-D02.3,D03-D06.9,D07.0-D07.2,D07.4,D07.5,D09.0,D09.3-D09.8,D10.0-D10.7,D11-D12.9,D13.0-D13.7,D14.0-D14.32,D15,D16.9,D22-D25.9,D26.0,D26.1,D27-D27.9,D28.0-D28.7,D29.0-D29.8,D30.0-D30.8,D31-D36,D36.1-D36.7,D37.01-D37.5,D38.0-D38.5,D39.1-D39.8,D40.0-D40.8,D41.0-D41.8,D42-D43.9,D44.0-D44.8,D45-D47.9,D48.0-D48.62,D49.2-D49.4,D49.6,D49.81,K31.7,K62.0,K62.1,K63.5,N84.0,N84.1,N87-N87.9 |
| 3 | Esophageal cancer | C15-C15.9,D00.1,D13.0 |
| 3 | Stomach cancer | C16-C16.9,D00.2,D13.1,D37.1 |
| 3 | Liver cancer | C22-C22.9,D13.4 |
| 3 | Lung, bronchus, and trachea cancer | C33-C34.92,D02.1-D02.3,D14.2-D14.32,D38.1 |
| 3 | Breast cancer | C50-C50.929,D05-D05.92,D24-D24.9,D48.6-D48.62,D49.3 |
| 3 | Cervical cancer | C53-C53.9,D06-D06.9,D26.0,D26.1,N84.1 |
| 3 | Uterine cancer | C54-C54.9,D25-D25.9,N84.0,N87-N87.9 |
| 3 | Prostate cancer | C61-C61.9,D29.1,D40.0 |
| 3 | Colon and rectum cancer | C18-C20.0,C20.9-C21.8,D01.0-D01.3,D12-D12.9,D37.3-D37.5,K62.0,K62.1,K63.5 |
| 3 | Nasopharynx cancer | C11-C11.9,D00.08,D10.6,D37.05 |
| 3 | Pancreatic cancer | C25-C25.9,D13.6,D13.7 |
| 3 | Ovarian cancer | C56-C56.9,D27-D27.9,D39.1-D39.12 |
| 3 | Kidney cancer | C64-C65.9,D30.0-D30.12,D41.0-D41.12 |
| 3 | Bladder cancer | C67-C67.9,D09.0,D30.3,D41.4-D41.8,D49.4 |
| 3 | Thyroid cancer | C73-C73.9,D09.3-D09.8,D34-D34.9,D44.0 |
| 3 | Leukemia | C91-C95.92 |
| 2 | Cardiovascular diseases | G45-G46.8,I01-I01.9,I02.0,I05-I09.9,I11-I11.9,I20-I25.9,I28-I28.8,I30-I31.1,I31.8,I31.9,I33-I42.9,I47-I48.92,I51.0-I51.6,I60-I61.9,I62.0-I62.03,I63-I63.9,I65-I66.9,I67.0-I67.3,I67.5-I67.7,I69.0-I69.198,I69.20-I69.398,I70.2-I70.8,I71-I78.9,I80-I83.93,I86-I89.9,I91.9 |
| 3 | Rheumatic heart disease | I01-I01.9,I02.0,I05-I09.9 |
| 3 | Ischemic heart disease | I20-I25.9 |
| 3 | Cerebrovascular disease | G45-G46.8,I60-I61.9,I62.0-I62.03,I63-I63.9,I65-I66.9,I67.0-I67.3,I67.5-I67.7,I69.0-I69.198,I69.20-I69.398 |
| 4 | Ischemic stroke | G45-G46.8,I63-I63.9,I65-I66.9,I67.2,I67.3,I67.5,I67.6,I69.3-I69.398 |
| 4 | Hemorrhagic stroke | I60-I61.9,I62.0-I62.03,I67.0,I67.1,I67.7,I69.0-I69.198,I69.20-I69.298 |
| 3 | Hypertensive heart disease | I11-I11.9 |
| 3 | Cardiomyopathy and myocarditis | I40-I42.9,I51.4-I51.6 |
| 3 | Atrial fibrillation and flutter | I48-I48.92 |
| 3 | Aortic aneurysm | I71-I71.9 |
| 2 | Chronic respiratory diseases | D86-D86.2,D86.89,D86.9,G47.3-G47.39,J30-J35.9,J37-J47.9,J60-J63.8,J65-J68.9,J70-J70.1,J70.8,J70.9,J82,J84- J84.9,J91-J92.9 |
| 3 | Chronic obstructive pulmonary disease | J40-J44.9,J47-J47.9 |
| 3 | Asthma | J45-J46.9 |
| 2 | Cirrhosis | B18-B18.9,D09.2-D09.22,I85-I85.9,K70-K70.9,K71.3-K71.51,K71.7,K72.1-K74.69,K74.9,K75.8- K76.0,K76.6,K76.7,K76.9 |
| 3 | Cirrhosis due to alcohol use | D09.2-D09.22 |
| 2 | Digestive diseases | I84-I84.9,K20-K22.9,K25-K29.91,K31-K31.6,K31.8-K31.89,K35-K38.9,K40-K42.9,K44-K46.9,K50- K52.9,K55-K57.93,K58.0-K62,K62.2-K62.6,K62.8-K62.9,K64-K64.9,K66.8,K67,K68-K68.9,K75.2-K75.4,K76.1-K76.5,K76.8-K76.89,K77-K77.8,K80-K83.9,K85-K86.9,K90-K90.9,K92.8-K92.89,M09.1 |
| 3 | Pepticulcer disease | K25-K28.9,K31,K31.1-K31.6,K31.8,K31.82-K31.89 |
| 3 | Pancreatitis | K85-K86.9 |
| 2 | Neurological disorders | F00-F03.91,G10-G13.8,G20-G21.0,G21.2-G24,G24.1-G25.0,G25.2,G25.3,G25.5,G25.8-G26.0,G30-G31.9,G35-G37.9,G40-G41.9,G61-G61.9,G70-G72,G72.2-G73.7,G90-G90.9,G95-G95.9,M33-M33.99 |
| 3 | Alzheimer disease and other dementias | F00-F03.91,G30-G31.9 |
| 3 | Parkinson disease | G20-G21.0,G21.2-G22.0 |
| 3 | Epilepsy | G40-G41.9 |
| 2 | Mental and substance use disorders | F06.2,F10-F11.99,F13-F16.99,F18-F23.9,F25-F29.9,F50.0-F50.1,G72.1,P96.1,Q86.0,R78.0-R78.5,X45- X45.9,X49-X49.9 |
| 3 | Schizophrenia | F06.2,F20-F23.9,F25-F29.9 |
| 3 | Drug use disorders | F11-F11.99,F13-F16.99,F18-F19.99,P96.1,R78.1-R78.5,X49-X49.9 |
| 2 | Diabetes, urogenital, blood, and endocrine diseases | B37.3-B37.49,B37.9,D52.1,D55-D58.9,D59.0-D59.3,D59.5,D59.6,D60-D61.9,D64.0,D64.4,D66-D69.8,D70-D75.89,D76-D78.89,D80-D83.9,D84.0-D84.8,D86.3-D86.87,D89-D89.3,E03-E07.1,E09-E16.9,E20-E34.8,E36-E36.8,E65-E68,E70-E85.29,E87.71,E88-E89.9,G21.1-G21.19,G24.0-G24.09,G25.1,G25.4,G25.6-G25.79,G72.0,G93.7,G97-G97.9,I12-I13.9,I95.2,I95.3,I97-I98.2,I98.9,J70.2-J70.5,J95-J95.9,K43-K43.9,K62.7,K91-K92,K94-K95.89,M87.1-M87.19,N00-N08.8,N10-N12.9,N14-N16.8,N18-N18.9,N20-N23.0,N25-N32.0,N32.3,N32.4,N34-N34.3,N36-N36.9,N39-N39.2,N41-N41.9,N44-N45.9,N49-N51.8,N65-N65.1,N72,N72.0,N75-N77.8,N80-N81.9,N83-N83.9,N99-N99.9,P03.2-P03.5,P70.0-P70.2,P96.2,P96.5,R50.2,R50.82,R50.83,R73-R73.9 |
| 3 | Diabetes mellitus | E10-E10.11,E10.3-E11.1,E11.3-E12.1,E12.3-E13.11,E13.3-E14.1,E14.3-E14.9,P70.0-P70.2,R73-R73.9 |
| 3 | Chronic kidney disease | E10.2-E10.29,E11.2-E11.29,E12.2,E13.2-E13.29,E14.2,I12-I13.9,N02-N08.8,N15.0,N18-N18.9 |
| 3 | Gynecological diseases | B37.3-B37.49,B37.9,E28.2,N72,N72.0,N75-N77.8,N80-N81.9,N83-N83.9 |
| 3 | Hemoglobinopathies and hemolytic anemias | D55-D58.9,D59.1,D59.3,D59.5,D60-D61.09,D61.2-D61.9,D64.0,D64.4 |
| 2 | Musculoskeletal disorders | I27.1,L93-L93.2,M00-M03.0,M03.2,M03.6,M05-M09.0,M09.2,M09.8,M30-M32.9,M34-M36.8,M40- M43.19,M65-M65.08,M71.0-M71.19,M86-M87.09,M88-M89.09,M89.5-M89.59,M89.7-M89.9 |
| 3 | Rheumatoid arthritis | M05-M06.9,M08.0-M08.89 |
| 2 | Other non-communicable diseases | A31.1,A31.2,A46,A46.0,L00-L05.92,L08-L08.9,L10-L14.0,L51-L51.9,L88-L89.95,L97-L98.499,P96.0,Q00-Q01.9,Q02-Q04.9,Q05-Q05.9,Q06-Q07.9,Q10.4-Q18.9,Q20-Q28.9,Q30-Q36,Q37-Q45.9,Q50-Q86,Q86.1-Q87.89,Q89-Q89.8,Q90-Q93.9,Q95-Q95.9,Q97-Q97.9,Q99-Q99.8,R95 |
| 3 | Congenital anomalies | P96.0,Q00-Q07.9,Q10.4-Q18.9,Q20-Q28.9,Q30-Q36,Q37-Q45.9,Q50-Q86,Q86.1-Q87.89,Q89-Q89.8,Q90- Q93.9,Q95-Q95.9,Q97-Q97.9,Q99-Q99.8 |
| 3 | Skin and subcutaneous diseases | A31.1,A31.2,A46,A46.0,L00-L05.92,L08-L08.9,L10-L14.0,L51-L51.9,L88-L89.95,L97-L98.499 |
| 1 | Injuries | V00-V86.99,V87.2,V87.3,V88.2,V88.3,V90,V90.0,V90.01-V98.8,W00-W46.2,W49-W62.9,W64-W70.9,W73-W81.9,W83-W94.9,W97.9,W99-X06.9,X08-X40.9,X43-X43.9,X46-X48.9,X50-X54.9,X57-X58.9,X60-Y08.9,Y35-Y84.9,Y87.1,Y88-Y88.3,Y89.0,Y89.1 |
| 2 | Transport injuries | V00-V86.99,V87.2,V87.3,V88.2,V88.3,V91-V91.9,V93-V98.8 |
| 3 | Road injuries | V01-V04.99,V06-V80.929,V82-V82.9,V87.2,V87.3 |
| 3 | Other transport injuries | V00-V00.898,V01-V04.99,V05-V05.99,V06-V09.9,V10-V19.9,V20-V29.9,V30-V79.9,V80-V80.929,V81-V81.9,V82-V82.9,V83-V86.99,V87.2,V87.3,V88.2,V88.3,V91-V91.9,V93-V98.8 |
| 2 | Unintentional injuries | V90,V90.0,V90.01-V90.9,V92-V92.9,W00-W46.2,W49-W62.9,W64-W70.9,W73-W81.9,W83- W94.9,W97.9,W99-X06.9,X08-X29.9,X40-X40.9,X43-X43.9,X46-X48.9,X50-X54.9,X57-X58.9,Y38.9- Y84.9,Y88-Y88.3 |
| 3 | Falls | W00-W19.9 |
| 3 | Drowning | V90,V90.0,V90.01-V90.9,V92-V92.9,W65-W70.9,W73-W74.9 |
| 3 | Fire, heat , and hot substances | X00-X06.9,X08-X19.9,X20.5 |
| 3 | Poisonings | X40-X40.9,X43-X43.9,X46-X48.9 |
| 2 | Self-harm and interpersonal violence | X60-Y08.9,Y87.1 |
| 3 | Self-harm | X60-X84.9 |
| 3 | Interpersonal violence | X85-Y08.9,Y87.1 |
| 2 | Others | U00-U04,U05-U99 |
| 4 | Other diarrheal diseases, lower respiratory and common infectious disease | A00-A00.9,A01.0-A01.09,A01.1-A01.4,A02-A02.9,A03-A03.9,A04,A04.1-A04.8, A04.9,A05-A05.9,A06-A06.9,A07,A07.0,A07.1, A07.2,A07.3,A07.4,A07.8,A07.9,A08,A08.0-A09.9,A33-A37.91,A39-A39.9,A83-A86.4,A87-A87.9,B01-B02.9,B94.1,F07.1,J01-J01.91,G00.0,G00.1,G00.2-G00.8,G03-G03.8,G04-G05.8,J05-J05.11,J09-J15.8,J16-J16.9,J20-J21.9,J36,J36.0,P23-P23.9,P35.8,H70-H70.93,R19.7 |
| 3 | Other neglected tropical diseases | A68-A68.9,A69.2-A69.5,A75-A75.9,A77-A79.9,A82-A82.9,A90-A91.9,A92-A94.0,A95-A96.9,A98-A98.8,B55.0-B56.9,B57-B57.5,B58,B59-B60.8,B65-B69.9,B70-B72.0,B74.3-B75,B78-B78.9,B80-B81.8,B83-B83.8,P37.1 |
| 3 | Other nutritional deficiencies | D51-D52.0,D52.8-D53.9,E00-E02,E40-E46.9,E51-E61.9,E63-E64,E64.0,E64.2,E64.3,M12.1-M12.19 |
| 4 | Other hepatitis | B17.2 |
| 2 | Other communicable, maternal, neonatal, and nutritional diseases | A20-A28.9,A31,A31.0,A31.8-A32.9,A38-A38.9,A42-A44.9,A48.1,A48.2,A48.4-A48.52,A49.1,A50-A53.9,A54-A56.8,A57-A58,A63-A63.8,A65-A67.9,A69-A69.1,A69.8-A70,A74,A74.8-A74.9,A80-A81.9,A88-A89.9,B00-B00.9,B03,B04,B06-B06.9,B10-B10.89,B20.0,B20.1-B23.9,B24.0,B25-B27.99,B29.4,B33-B33.8,B37-B37.2,B37.5-B37.89,B38-B44.9,B45-B45.9,B46-B49.9,B58.0-B58.9,B91,B95-B95.5,G14,G14.6,I00,I02,I02.9,J02.0,J03.0-J03.01,K67.0,K67.1,K67.2,K67.8,K74.7,K74.8,M89.6-M89.69,M03.1,M49.1,M73.0,M73.1,N96,N98-N98.9,O09-O09.93,O10-O16.9,O20-O20.9,O21-O22.93,O24-O25.3,O26-O26.93,O28-O31.8,O32-O33.9,O34-O36.93,O40-O43.199,O43.2-O43.239,O43.8-O43.93,O44-O46.93,O47-O48.1,O60-O63.9,O64-O66.9,O68-O69.9,O70-O84.9,O67-O67.9,O70,O72-O72.3,O23-O23.93,O85-O86.89,O87-O90.9,O91-O91.23,O92-O92.79,O96-O99.9,P00-P01.6,P01.7-P03.1,P03.6-P03.9,P04-P05.9,P07-P07.39,P08-P09,P10-P15.9,P19-P19.9,P20-P21.9,P22-P22.9,P24-P24.9,P25-P29.9,P35-P35.2,P35.9,P36-P36.9,P37,P37.2,P37.5-P37.9,P38-P39.9,P50-P59.9,P60-P61.2,P61.3-P61.9,P70,P70.3-P72.9,P74-P76.9,P77-P77.9,P78-P78.9,P80-P81.9,P83-P84,P90-P91.9,P92-P94.9,P96,P96.3,P96.4,P96.8-P96.89 |
| 3 | Other neoplasms | C00-C08.9,C09-C10.9,C12-C13.9,C17-C17.9,C20.8,C23-C24.9,C30-C32.9,C37-C38.8,C40-C41.9,C43-C43.9,C44,C45-C45.9,C47-C49.9,C51-C52.9,C57-C57.8,C58,C58.0,C60-C60.9,C62-C62.92,C63-C63.8,C66-C66.9,C68.0-C68.8,C69-C69.92,C70-C72.9,C74-C75.8,C81-C81.99,C82-C86.6,C88-C90.32,C96-C97.9D00.00-D00.07,D02.0,D03-D03.9,D04-D04.9,D07.0-D07.2,D07.4,D07.5,D10.0-D10.5,D10.7,D11-D11.9,D13.2-D13.39,D13.5,D14.0,D14.1,D15-D16.9,D22-D23.9,D28.0-D28.7,D29.0,D29.2-D29.8,D30.2-D30.22,D30.4-D30.8,D31-D33.9,D35-D36,D36.1-D36.7,D37.01-D37.04,D37.2,D37.09,D38.0,D38.2-D38.5,D39.2-D39.8,D40.1-D40.8,D41.2-D41.3,D42-D43.9,D44.1-C44.99,D45-D47.9,D48.0-D48.5,D49.2,D49.6,D49.81,K31.7 |
| 3 | Other cardiovascular and circulatory diseases | I28-I28.8,I30-I31.1,I31.8,I31.9,I33-I33.9,I34-I38.9,I39-I39.9,I47-I47.9,I51.0-I51.3,I70.2-I70.799,I70.8,I72-I72.9,I73-I73.9,I74-I78.9,I80-I83.93,I86- I89.9,I91.9 |
| 3 | Other chronic respiratory diseases | G47.3-G47.39,J30-J35.9,J37-J39.9,J61,J61.0,,J60-J63.8,J62-J62.9,J63-J63.8,J65,J65.0,J66-J68.9,J70-J70.1,J70.8,J70.9,J82,J84-J84.9,J91-J92,J92.0,J92.9,D86-D86.2,D86.89,D86.9 |
| 3 | Other digestive diseases | I84-I84.9,K20-K22.9,K29-K29.91,K31.0,K31.81-K31.819,K35-K37.9,K38-K38.9,K40-K42.9,K44-K46.9,K50-K52.9,K55-K55.9,K56-K56.9,K57-K57.93,K58.0,K58.9-K62,K62.2-K62.6,K62.8- K62.9,K64-K64.9,K66.8,K67,K68-K68.9,K75.2-K75.4,K76.1-K76.5,K76.8-K76.89,K77-K77.8,K80-K83.9.K90-K90.9,K92.8-K92.89,M09.1 |
| 3 | Other neurological disorders | G10-G13.8,G23-G24,G24.1-G25.0,G25.2,G25.3,G25.5,G25.8-G26.0,G35-G35.9,G36-G37.9,G61-G61.9,G70-G72,G72.2- G73.7,G90-G90.9,G95-G95.9,M33-M33.99 |
| 3 | other metal and substance use disorders | F10-F10.99,F11-F11.99,F13-F13.99,F14-F14.99,F15-F15.99,F16-F16.99,F18-F19.99,F50.0-F50.1,G72.1,P96.1,Q86.0,R78.0,R78.1,R78.2,R78.3-R78.5,X45-X45.9,X49-X49.9 |
| 3 | Other diabetes, urogenital, blood, and endocrine diseases | B37.3-B37.49,B37.9,D52.1,D55-D55.9,D56-D56.9,D57-D57.819,D58-D58.9,,D59.0,D59.1,D59.2,D59.3,D59.5,D59.6,D60-D61.09,D61.1,D61.2-D61.9,D64.0,D64.4,D66-D69.8,D70-D75.89,D76-D78.89,D80-D83.9,D84.0-D84.8,D86.3-D86.87,D89-D89.3,E03-E07.1,E09-E09.9,E10.2-E10.29,E11.2-E11.29,E12.2,E13.2-E13.29,E14.2,E15-E16.9,E20-E28.1,E28.2,E28.3-E34.8,E36-E36.8,E65-E68,E70-E85.29,E87.71,E88-E89.9,G21.1-G21.19,G24.0-G24.09,G25.1,G25.4,G25.6-G25.79,G72.0,G93.7,G97-G97.9,I12-I13.9,I95.2,I95.3,I97-I98.2,I98.9,J70.2-J70.5,J95-J95.9,K43-K43.9,K62.7,K91-K92,K94-K95.89,M87.1-M87.19,N00-N01.9,N02-N02.9,N03-N06.9,N07-N08.8,N10-N12.9,N14-N14.4,N15.0,N15,N15.1-N16.8,N20-N23.0,N25-N32.0,N32.3,N32.4,N34-N34.3,N32.3,N32.4,N34-N34.3,N36-N36.9,N39-N39.2,N41-N41.9,N44-N45.9,N49-N51.8,N65-N65.1,N72,N72.0,N75-N77.8,N80-N80.9,N81-N81.9,N83-N83.9,N99-N99.9,P03.2-P03.5,P96.2,P96.5,R50.2,R50.82,R50.83 |
| 3 | Other musculoskeletal disorders | I27.1,L93-L93.2,M00-M03.0,M03.2,M03.6,M07-M08,M08.9-M09.0,M09.2,M09.8,M30-M32.9,M34-M36.8,M40-M43.19,M65-M65.08,M71.0-M71.19,M86-M87.09,M88-M89.09,M89.5-M89.59,M89.7-M89.9 |
| 3 | Other unintentional injuries | W20-W38.9,W20-W31.9,W32-W34.9,W35-W38.9,W39-W39.9,W40-W43.9,W44-W45,W45.3-W45.9,W45.0-W45.2,W46-W46.2,W49-W52,W52.0-W52.2,W52.3,W52.0-W52.2,W52.4-W62.9,W64-W64.9,W75-W76.9,W77-W77.9,W78-W80.9,W81-W81.9,W83-W84.9,W85-W94.9,W97.9,W99-W99.9,X20-X20.4,X20.6-X29.9,X50-X54.9,X57-X58.9,Y38.9-Y84.9,Y88-Y88.3 |
| 3 | Other injury | X30-X39.9,Y35-Y38.893,X85-X92.9,X93-X94.0,,X94.1,X94.2,X94.3-X94.7,X94.8,X94.9-X95.9,X96-X96.4,X96.5,X96.6-X98.9,X99-X99.9,Y00-Y08.9,Y87.1 |

**Reference**

1. Mathers CD, Loncar D. Projections of global mortality and burden of disease from 2002 to 2030. PLoS Med. 2006 Nov;3(11):e442.
2. WHO methods and data sources for life tables 1990-2016. <http://www.who.int/healthinfo/statistics/LT_method.pdf?ua=1>. Access: Aug, 2018.
3. WHO methods and data sources for global causes of death 2000‐2011. <http://www.who.int/healthinfo/statistics/GHE_TR2013-3_COD_MethodsFinal.pdf?ua=1.> Access: Aug, 2018.
4. Cooke D, Leon JA. Stability of population growth determined by 2 X 2 Leslie matrix with density-dependent elements. Biometrics. 1976; 32(2):435-442.
5. Zhou M, Wang H, Zhu J, Chen W, Wang L, Liu S, et al. [Cause-specific mortality for 240 causes in China during 1990-2013: a systematic subnational analysis for the Global Burden of Disease Study 2013.](https://www.ncbi.nlm.nih.gov/pubmed/26510778) Lancet. 2016; 387:251-72.
6. National Disease Surveillance System monitoring causes of death 2010. Chinese Center for Disease Control and Prevention, Beijing, Military Medical Science Press, 2012, ISBN 978-7-80245-827-7.
7. Mathers CD, Fat DM, Inoue M, Rao C and Lopez AD. "Counting the dead and what they died from: an assessment of the global status of cause of death data". [Bull World Health Organ.](https://www.ncbi.nlm.nih.gov/pubmed/?term=Counting+the+dead+and+what+they+died+from:+an+assessment+of+the+global+status+of+cause+of+death+data) 2005; 83(3):171-7.
8. Murray CJL and Lopez AD. 1996. "Alternative visions of the future: projecting mortality and disability, 1990-2020". In The Global Burden of Disease, edited by Murray, C. J.L and A. D. Lopez Vol. 1, Global Burden of Disease and Injury Series. (Cambridge: Harvard University Press).
9. Murray CJL and Lopez AD. 1996. The Global Burden of Disease: a comprehensive assessment of mortality and disability from diseases, injuries and risk factors in 1990 and projected to 2020. 1 ed., Global Burden of Disease and Injury Series. Cambridge: Harvard University Press.
10. Peto R, Lopez AD, Boreham J, Thun M, and Heath Jr C. 1992. "Mortality from tobacco in developed countries: indirect estimation from National Vital Statistics". Lancet 339, no. 8804:1268-1278.
11. GBD 2016 Causes of Death Collaborators. Global, regional, and national age-sex specific mortality for 264 causes of death, 1980-2016: a systematic analysis for the Global Burden of Disease Study 2016. Lancet. 2017; 390 :1151-1210.
12. Mathers CD, Lopez AD, Murray CJL, Ezzati M, Jamison DT. The burden of disease and mortality by condition: data, methods and results for 2001. Global burden of disease and risk factors. New York, Oxford University Press, 2006. p. 45-240.
13. Lim SS, Vos T, Flaxman AD, et al. A comparative risk assessment of burden of disease and injury attributable to 67 risk factors and risk factor clusters in 21 regions, 1990-2010: a systematic analysis for the Global Burden of Disease Study 2010. Lancet. 2012; 380(9859):2224-2260.
14. Lozano R, Naghavi M, Foreman K, et al. Global and regional mortality from 235 causes of death for 20 age groups in 1990 and 2010: a systematic analysis for the Global Burden of Disease Study 2010. Lancet. 2012; 380(9859):2095-2128.
15. Murray CJ, Ezzati M, Flaxman AD, et al. GBD 2010: design, definitions, and metrics. Lancet. 2012; 380(9859): 2063-2066.
16. GBD 2016 Disease and Injury Incidence and Prevalence Collaborators. Global, regional, and national incidence, prevalence, and years lived with disability for 328 diseases and injuries for 195 countries, 1990-2016: a systematic analysis for the Global Burden of Disease Study 2016. Lancet 2017; 390: 1211-59.
17. Murray CJL,Lopez,et al.Global Comparative assessment in the health sector: Quantifying disability: data, methods and results. Geneva: World Health Organization. 1994.
18. Sullivan DF. A single index of mortality and morbidity. HSMHA Health Rep 1971; 86: 347-54.

**Results**

**E-Table 1. Age-Standardized mortality rates for 116 diseases of male in Hakka, Canton and Hoklo cultural regions, between 2005 and 2015**

| **All cause of death** | **Hakka culture region** | | | **Canton culture region** | | | **Hoklo culture region** | | | **P value** |
| --- | --- | --- | --- | --- | --- | --- | --- | --- | --- | --- |
|  | **2005** | **2015** | **Change** | **2005** | **2015** | **Change** | **2005** | **2015** | **Change** |  |
| All cause of death | 761.50 | 603.42 | -20.76 | 572.39 | 471.97 | -17.54^*^ | 760.15 | 628.86 | -17.27^*^ | <0.001^#^ |
| Communicable, maternal, neonatal, and nutritional diseases | 44.68 | 42.55 | -4.77^*^ | 33.14 | 28.93 | -12.70^*^ | 46.22 | 42.16 | -8.78^*^ | <0.001^#^ |
| HIV/AIDS and tuberculosis | 11.85 | 11.85 | 0.00^*^ | 8.30 | 6.55 | -21.08 | 13.69 | 13.36 | -2.41^*^ | <0.001^#^ |
| Tuberculosis | 9.61 | 8.68 | -9.68^*^ | 5.59 | 3.57 | -36.14^*^ | 11.74 | 10.70 | -8.86^*^ | <0.001^#^ |
| HIV/AIDS | 2.24 | 3.17 | 41.52^*^ | 2.71 | 2.98 | 9.96^*^ | 1.94 | 2.67 | 37.63^*^ | 0.109 |
| Diarrhoea, lower respiratory and other common infectious diseases | 22.16 | 20.57 | -7.18^*^ | 17.94 | 15.50 | -13.60^*^ | 21.51 | 19.38 | -9.90^*^ | 0.097 |
| Diarrhoeal diseases | 3.72 | 2.37 | -36.29 | 2.44 | 1.40 | -42.62 | 3.33 | 2.18 | -34.53^*^ | 0.122 |
| Intestinal infectious diseases | 0.73 | 0.92 | 26.03 | 1.13 | 0.94 | -16.81 | 0.65 | 0.72 | 10.77 | 0.889 |
| Lower respiratory infections | 8.77 | 12.54 | 42.99^*^ | 7.87 | 10.04 | 27.57^*^ | 8.45 | 11.89 | 40.71 ^*^ | 0.467 |
| Meningitis | 5.65 | 2.95 | -47.79^*^ | 3.37 | 1.66 | -50.74^*^ | 5.36 | 2.79 | -47.95^*^ | <0.001^#^ |
| Measles | 2.13 | 0.92 | -56.81^*^ | 2.10 | 0.75 | -64.29^*^ | 2.58 | 1.02 | -60.47^*^ | <0.001^#^ |
| Neglected tropical diseases and malaria | 0.83 | 0.36 | -56.63^*^ | 0.67 | 0.24 | -64.18^*^ | 0.78 | 0.32 | -58.97 | 0.011^#^ |
| Malaria | 0.09 | 0.06 | -33.33 | 0.08 | 0.03 | -62.50 | 0.08 | 0.05 | -37.50 | 0.988 |
| Rabies | 0.60 | 0.20 | -66.67^*^ | 0.48 | 0.15 | -68.75^*^ | 0.57 | 0.18 | -68.42^*^ | 0.397 |
| Intestinal nematode infections | 0.00 | 0.00 | 0.00 | 0.00 | 0.00 | 0.00 | 0.00 | 0.00 | 0.00 | - |
| Maternal disorders | 0.00 | 0.00 | 0.00 | 0.00 | 0.00 | 0.00 | 0.00 | 0.00 | 0.00 | - |
| Neonatal disorders | 6.24 | 7.39 | 18.43^*^ | 4.39 | 5.45 | 24.15^*^ | 5.95 | 6.27 | 5.38^*^ | <0.001^#^ |
| Nutritional deficiencies | 0.96 | 1.15 | 19.79^*^ | 0.61 | 0.66 | 8.20 | 1.10 | 1.29 | 17.27^*^ | 0.002^#^ |
| Iron-deficiency anemia | 0.01 | 0.01 | 0.00 | 0.01 | 0.01 | 0.00 | 0.01 | 0.01 | 0.00^*^ | 0.994 |
| Other communicable, maternal, neonatal, and nutritional diseases | 2.64 | 1.24 | -53.03^*^ | 1.23 | 0.53 | -56.91^*^ | 3.19 | 1.55 | -51.41^*^ | <0.001^#^ |
| Sexually transmitted diseases excluding HIV | 0.48 | 0.30 | -37.50 | 0.42 | 0.15 | -64.29^*^ | 0.54 | 0.30 | -44.44 | 0.428 |
| Hepatitis | 1.95 | 0.75 | -61.54^*^ | 0.72 | 0.29 | -59.72^*^ | 2.40 | 1.04 | -56.67^*^ | <0.001^#^ |
| Acute hepatitis A | 0.27 | 0.23 | -14.81 | 0.20 | 0.14 | -30.00^*^ | 0.28 | 0.26 | -7.14 | 0.275 |
| Acute hepatitis B | 1.21 | 0.34 | -71.90^*^ | 0.31 | 0.08 | -74.19 | 1.59 | 0.55 | -65.41^*^ | <0.001^#^ |
| Acute hepatitis C | 0.22 | 0.08 | -63.64 | 0.11 | 0.03 | -72.73^*^ | 0.23 | 0.10 | -56.52^*^ | 0.62 |
| Non-communicable diseases | 633.73 | 515.74 | -18.62^*^ | 492.61 | 414.67 | -15.82^*^ | 636.92 | 541.95 | -14.91^*^ | <0.001^#^ |
| Neoplasms | 196.53 | 166.17 | -15.45^*^ | 156.93 | 133.64 | -14.84^*^ | 198.37 | 178.20 | -10.17^*^ | <0.001^#^ |
| esophageal cancer | 12.79 | 10.76 | -15.87^*^ | 10.54 | 8.59 | -18.50^*^ | 13.79 | 12.63 | -8.41 | <0.001^#^ |
| Stomach cancer | 24.70 | 20.24 | -18.06^*^ | 18.00 | 13.97 | -22.39^*^ | 25.33 | 22.11 | -12.71^*^ | <0.001^#^ |
| Liver cancer | 51.78 | 40.32 | -22.13^*^ | 38.65 | 29.93 | -22.56^*^ | 49.33 | 41.31 | -16.26^*^ | <0.001^#^ |
| Tracheal, bronchus, and lung cancer | 43.89 | 38.80 | -11.60^*^ | 33.25 | 31.30 | -5.86^*^ | 44.43 | 40.37 | -9.14^*^ | <0.001^#^ |
| Breast cancer | 0.99 | 0.64 | -35.35 | 0.72 | 0.45 | -37.50 | 0.98 | 0.66 | -32.65^*^ | 0.663 |
| Cervical cancer | 0.00 | 0.00 | 0.00 | 0.00 | 0.00 | 0.00 | 0.00 | 0.00 | 0.00 | - |
| Uterine cancer | 0.00 | 0.00 | 0.00 | 0.00 | 0.00 | 0.00 | 0.00 | 0.00 | 0.00 | - |
| Prostate cancer | 0.81 | 0.72 | -11.11 | 0.78 | 0.68 | -12.82^*^ | 0.83 | 0.78 | -6.02 | 0.335 |
| Colon and rectum cancer | 8.28 | 9.84 | 18.84^*^ | 8.94 | 10.50 | 17.45^*^ | 8.75 | 11.19 | 27.89^*^ | <0.001^#^ |
| Nasopharyngeal cancer | 20.56 | 14.22 | -30.84^*^ | 17.55 | 12.00 | -31.62^*^ | 21.59 | 16.13 | -25.29^*^ | <0.001^#^ |
| Pancreatic cancer | 3.74 | 4.30 | 14.97 | 4.07 | 4.09 | 0.49 | 3.98 | 4.70 | 18.09 | 0.011^#^ |
| Ovarian cancer | 0.00 | 0.00 | 0.00 | 0.00 | 0.00 | 0.00 | 0.00 | 0.00 | 0.00 | - |
| Kidney cancer | 1.29 | 1.31 | 1.55 | 1.53 | 1.47 | -3.92 | 1.39 | 1.49 | 7.19 | 0.001^#^ |
| Bladder cancer | 1.71 | 1.19 | -30.41^*^ | 1.50 | 1.00 | -33.33^*^ | 1.93 | 1.43 | -25.91^*^ | <0.001^#^ |
| Thyroid cancer | 0.88 | 0.69 | -21.59 | 0.85 | 0.65 | -23.53 | 0.79 | 0.65 | -17.72 | 0.812 |
| Leukemia | 5.96 | 4.79 | -19.63 | 5.04 | 4.11 | -18.45^*^ | 5.97 | 5.10 | -14.57^*^ | <0.001^#^ |
| Cardiovascular diseases | 246.63 | 210.97 | -14.46^*^ | 202.23 | 180.18 | -10.90^*^ | 243.66 | 218.88 | -10.17 | <0.001^#^ |
| Rheumatic heart disease | 4.55 | 2.68 | -41.10^*^ | 3.43 | 2.18 | -36.44^*^ | 4.47 | 2.79 | -37.58^*^ | <0.001^#^ |
| Ischemic heart disease | 101.62 | 93.94 | -7.56^*^ | 93.15 | 88.55 | -4.94^*^ | 98.16 | 95.47 | -2.74^*^ | <0.001^#^ |
| Cerebrovascular disease | 121.55 | 91.92 | -24.38^*^ | 88.83 | 68.61 | -22.76^*^ | 123.18 | 99.05 | -19.59^*^ | <0.001^#^ |
| Ischemic stroke | 49.26 | 34.19 | -30.59 | 37.13 | 26.71 | -28.06^*^ | 50.37 | 37.86 | -24.84 | 0.044^#^ |
| Hemorrhagic stroke | 72.29 | 57.73 | -20.14^*^ | 51.70 | 41.90 | -18.96^*^ | 72.81 | 61.20 | -15.95^*^ | <0.001^#^ |
| Hypertensive heart disease | 9.36 | 13.36 | 42.74^*^ | 9.43 | 13.44 | 42.52^*^ | 8.49 | 12.26 | 44.41^*^ | <0.001^#^ |
| Cardiomyopathy and myocarditis | 2.11 | 1.79 | -15.17 | 1.33 | 1.21 | -9.02 | 2.04 | 1.81 | -11.27 | 0.236 |
| Atrial fibrillation and flutter | 1.53 | 1.86 | 21.57^*^ | 1.44 | 1.73 | 20.14 | 1.51 | 1.89 | 25.17^*^ | 0.568 |
| Aortic aneurysm | 1.73 | 1.77 | 2.31 | 1.29 | 1.40 | 8.53 | 1.71 | 1.86 | 8.77 | 0.056 |
| Chronic respiratory diseases | 103.71 | 66.65 | -35.73^*^ | 71.11 | 44.89 | -36.87 | 108.37 | 71.17 | -34.33^*^ | <0.001^#^ |
| Chronic obstructive pulmonary disease | 100.24 | 64.55 | -35.60^*^ | 68.82 | 43.36 | -37.00^*^ | 104.11 | 68.65 | -34.06^*^ | <0.001^#^ |
| Asthma | 2.95 | 1.30 | -55.93 | 1.99 | 1.04 | -47.74^*^ | 3.73 | 1.68 | -54.96^*^ | <0.001^#^ |
| Cirrhosis | 21.21 | 14.12 | -33.43^*^ | 12.74 | 8.46 | -33.59^*^ | 22.19 | 15.18 | -31.59^*^ | <0.001^#^ |
| Cirrhosis due to alcohol use | 0.00 | 0.00 | 0.00 | 0.00 | 0.00 | 0.00 | 0.00 | 0.00 | 0.00 | - |
| Digestive diseases | 12.36 | 8.47 | -31.47^*^ | 6.96 | 4.79 | -31.18^*^ | 12.58 | 8.92 | -29.09^*^ | 0.03^#^ |
| Peptic ulcer disease | 5.75 | 3.50 | -39.13^*^ | 2.96 | 1.81 | -38.85^*^ | 5.76 | 3.63 | -36.98^*^ | <0.001^#^ |
| Pancreatitis | 1.33 | 0.80 | -39.85^*^ | 0.93 | 0.57 | -38.71^*^ | 1.47 | 0.93 | -36.73^*^ | 0.002^#^ |
| Neurological disorders | 14.13 | 16.02 | 13.38^*^ | 15.38 | 17.73 | 15.28 ^*^ | 14.16 | 16.59 | 17.16^*^ | <0.001^#^ |
| Alzheimer’s disease and other dementias | 8.46 | 10.83 | 28.01^*^ | 9.56 | 12.17 | 27.30^*^ | 8.56 | 11.22 | 31.07^*^ | 0.669 |
| Parkinson’s disease | 3.61 | 2.91 | -19.39^*^ | 3.70 | 3.10 | -16.22^*^ | 3.54 | 2.98 | -15.82 | 0.022^#^ |
| Epilepsy | 1.34 | 1.72 | 28.36^*^ | 1.48 | 1.89 | 27.70^*^ | 1.36 | 1.79 | 31.62^*^ | 0.345 |
| hemicrania | 0.00 | 0.00 | 0.00 | 0.00 | 0.00 | 0.00 | 0.00 | 0.00 | 0.00 | - |
| Mental and substance use disorders | 5.49 | 3.58 | -34.79^*^ | 2.38 | 1.75 | -26.47^*^ | 4.98 | 3.41 | -31.53^*^ | <0.001^#^ |
| Schizophrenia | 0.91 | 0.93 | 2.20 | 0.57 | 0.61 | 7.02 | 0.78 | 0.88 | 12.82 | 0.001^#^ |
| Drug use disorders | 2.76 | 1.66 | -39.86 | 0.98 | 0.65 | -33.67^*^ | 2.51 | 1.58 | -37.05^*^ | 0.039^#^ |
| [Depressive](file:///D:\\Users\\nfernandez\\Program%20Files%20(x86)\\Youdao\\Dict\\7.5.2.0\\resultui\\dict\\%3fkeyword=depressive)[disorder](file:///D:\\Users\\nfernandez\\Program%20Files%20(x86)\\Youdao\\Dict\\7.5.2.0\\resultui\\dict\\%3fkeyword=disorder) | 0.00 | 0.00 | 0.00 | 0.00 | 0.00 | 0.00 | 0.00 | 0.00 | 0.00 | - |
| [infantile](file:///D:\\Users\\nfernandez\\Program%20Files%20(x86)\\Youdao\\Dict\\7.5.2.0\\resultui\\dict\\%3fkeyword=infantile)[autism](file:///D:\\Users\\nfernandez\\Program%20Files%20(x86)\\Youdao\\Dict\\7.5.2.0\\resultui\\dict\\%3fkeyword=autism) | 0.00 | 0.00 | 0.00 | 0.00 | 0.00 | 0.00 | 0.00 | 0.00 | 0.00 | - |
| Diabetes, urogenital, blood, and endocrine diseases | 27.64 | 22.81 | -17.47^*^ | 20.21 | 17.21 | -14.84^*^ | 27.19 | 23.49 | -13.61^*^ | <0.001^#^ |
| Diabetes mellitus | 8.41 | 7.58 | -9.87^*^ | 6.45 | 5.88 | -8.84 | 8.50 | 7.99 | -6.00^*^ | <0.001^#^ |
| Chronic kidney disease | 13.12 | 10.96 | -16.46^*^ | 9.49 | 8.02 | -15.49^*^ | 12.47 | 11.00 | -11.79 | <0.001^#^ |
| Gynecological diseases | 0.00 | 0.00 | 0.00 | 0.00 | 0.00 | 0.00 | 0.00 | 0.00 | 0.00 | - |
| Hemoglobinopathies and hemolytic anemia | 2.63 | 1.37 | -47.91^*^ | 1.66 | 1.01 | -39.16^*^ | 2.81 | 1.55 | -44.84^*^ | 0.011^#^ |
| Musculoskeletal disorders | 0.70 | 0.61 | -12.86 | 0.51 | 0.42 | -17.65 | 0.70 | 0.64 | -8.57 | 0.388 |
| Rheumatoid arthritis | 0.32 | 0.29 | -9.38 | 0.22 | 0.19 | -13.64 | 0.33 | 0.30 | -9.09 | 0.628 |
| [Osteoarthritis](file:///D:\\Users\\nfernandez\\Program%20Files%20(x86)\\Youdao\\Dict\\7.5.2.0\\resultui\\dict\\%3fkeyword=osteoarthritis) | 0.00 | 0.00 | 0.00 | 0.00 | 0.00 | 0.00 | 0.00 | 0.00 | 0.00 | - |
| Low back and neck pain | 0.00 | 0.00 | 0.00 | 0.00 | 0.00 | 0.00 | 0.00 | 0.00 | 0.00 | - |
| Low back pain | 0.00 | 0.00 | 0.00 | 0.00 | 0.00 | 0.00 | 0.00 | 0.00 | 0.00 | - |
| Neck pain | 0.00 | 0.00 | 0.00 | 0.00 | 0.00 | 0.00 | 0.00 | 0.00 | 0.00 | - |
| Other non-communicable diseases | 5.32 | 6.34 | 19.17 | 4.16 | 5.60 | 34.62^*^ | 4.72 | 5.47 | 15.89^*^ | 0.015^#^ |
| Congenital anomalies | 5.03 | 6.08 | 20.87 | 3.93 | 5.37 | 36.64 | 4.49 | 5.26 | 17.15^*^ | <0.001^#^ |
| Skin and subcutaneous diseases | 0.24 | 0.19 | -20.83 | 0.20 | 0.17 | -15.00^*^ | 0.19 | 0.15 | -21.05^*^ | 0.674 |
| Sense organ diseases | 0.00 | 0.00 | 0.00 | 0.00 | 0.00 | 0.00 | 0.00 | 0.00 | 0.00 | - |
| Oral disorders | 0.00 | 0.00 | 0.00 | 0.00 | 0.00 | 0.00 | 0.00 | 0.00 | 0.00 | - |
| Injuries | 83.09 | 45.13 | -45.69^*^ | 46.64 | 28.36 | -39.19^*^ | 77.01 | 44.75 | -41.89^*^ | <0.001^#^ |
| Transport injuries | 40.02 | 20.13 | -49.70^*^ | 21.29 | 11.92 | -44.01^*^ | 36.47 | 20.47 | -43.87^*^ | <0.001^#^ |
| Road injuries | 37.27 | 19.42 | -47.89^*^ | 20.64 | 11.67 | -43.46^*^ | 34.21 | 19.86 | -41.95^*^ | <0.001^#^ |
| Other transport injuries | 2.75 | 0.71 | -74.18^*^ | 0.65 | 0.25 | -61.54^*^ | 2.27 | 0.60 | -73.57^*^ | <0.001^#^ |
| Unintentional injuries | 27.18 | 17.64 | -35.10^*^ | 18.60 | 12.50 | -32.80^*^ | 24.64 | 16.53 | -32.91^*^ | <0.001^#^ |
| Falls | 8.21 | 6.46 | -21.32^*^ | 5.77 | 4.74 | -17.85^*^ | 7.42 | 6.10 | -17.79^*^ | 0.695 |
| Drowning | 7.47 | 4.24 | -43.24^*^ | 5.54 | 3.16 | -42.96^*^ | 6.79 | 3.97 | -41.53^*^ | 0.045^#^ |
| Fire, heat, and hot substances | 1.80 | 0.68 | -62.22^*^ | 0.90 | 0.37 | -58.89^*^ | 1.66 | 0.65 | -60.84^*^ | 0.446 |
| Poisonings | 2.40 | 1.47 | -38.75^*^ | 1.34 | 0.86 | -35.82^*^ | 2.17 | 1.38 | -36.41^*^ | 0.021^#^ |
| Intentional injury | 15.56 | 7.25 | -53.41^*^ | 6.57 | 3.88 | -40.94^*^ | 15.59 | 7.66 | -50.87^*^ | 0.659 |
| Self-harm and interpersonal violence | 10.85 | 5.45 | -49.77^*^ | 4.92 | 3.12 | -36.59^*^ | 10.80 | 5.79 | -46.39^*^ | <0.001^#^ |
| Interpersonal violence | 4.71 | 1.80 | -61.78^*^ | 1.65 | 0.76 | -53.94^*^ | 4.79 | 1.87 | -60.96^*^ | 0.494 |
| Other diarrhea, lower respiratory and common infectious disease | 1.17 | 0.88 | -24.79 | 1.03 | 0.70 | -32.04^*^ | 1.14 | 0.77 | -32.46 | 0.02^#^ |
| Other neglected tropical disease | 0.14 | 0.10 | -28.57 | 0.11 | 0.06 | -45.45^*^ | 0.14 | 0.09 | -35.71 | 0.65 |
| Other nutritional disease | 0.95 | 1.14 | 20.00^*^ | 0.60 | 0.65 | 8.33^*^ | 1.08 | 1.28 | 18.52^*^ | 0.004^#^ |
| Other hepatitis | 0.24 | 0.09 | -62.50 | 0.09 | 0.04 | -55.56^*^ | 0.30 | 0.13 | -56.67^*^ | 0.519 |
| Other Communicable, maternal, neonatal, and nutritional diseases | 0.21 | 0.19 | -9.52 | 0.09 | 0.09 | 0.00 ^*^ | 0.25 | 0.22 | -12.00^*^ | 0.14 |
| Other cancer | 19.15 | 18.34 | -4.23^*^ | 15.49 | 14.91 | -3.74^*^ | 19.29 | 19.64 | 1.81^*^ | <0.001^#^ |
| Other cardiovascular disease | 4.18 | 3.66 | -12.44^*^ | 3.32 | 3.06 | -7.83^*^ | 4.10 | 3.75 | -8.54 | 0.48 |
| Other chronic respiratory disease | 0.51 | 0.80 | 56.86^*^ | 0.30 | 0.49 | 63.33 | 0.53 | 0.84 | 58.49^*^ | 0.124 |
| Other Cirrhosis | 21.21 | 14.12 | -33.43^*^ | 12.74 | 8.46 | -33.59^*^ | 22.19 | 15.18 | -31.59^*^ | <0.001^#^ |
| Other digestive disease | 5.28 | 4.16 | -21.21^*^ | 3.07 | 2.41 | -21.50^*^ | 5.35 | 4.37 | -18.32^*^ | 0.001^#^ |
| Other Neurological disorders | 0.71 | 0.56 | -21.13 | 0.64 | 0.56 | -12.50 | 0.70 | 0.60 | -14.29^*^ | 0.6 |
| Other mental and substance use disorders | 1.83 | 0.98 | -46.45^*^ | 0.83 | 0.49 | -40.96^*^ | 1.70 | 0.96 | -43.53^*^ | 0.15 |
| Other diabetes, urogenital, blood, and endocrine diseases | 3.48 | 2.90 | -16.67 | 2.61 | 2.31 | -11.49^*^ | 3.40 | 2.96 | -12.94^*^ | 0.001^#^ |
| Other Musculoskeletal disorders | 0.37 | 0.32 | -13.51 | 0.29 | 0.23 | -20.69 | 0.37 | 0.34 | -8.11 | 0.396 |
| Others | 0.05 | 0.07 | 40.00 | 0.03 | 0.06 | 100.00 | 0.04 | 0.06 | 50.00 | 0.756 |
| Other unintentional injury | 7.31 | 4.79 | -34.47^*^ | 5.04 | 3.37 | -33.13 ^*^ | 6.61 | 4.43 | -32.98^*^ | 0.296 |
| Other injury | 0.33 | 0.10 | -69.70 | 0.19 | 0.07 | -63.16 ^*^ | 0.30 | 0.10 | -66.67 | 0.532 |

***: Statistical significance using Cochran-Armitage trend test for year 2005 to 2015 comparison (P< 0.05); #: statistical significance using poisson regression model**

**for culture regions comparison (P< 0.05).**

**E-Table 2. Age-Standardized mortality rates for 116 diseases of female in Hakka, Canton and Hoklo cultural regions, between 2005 and 2015**

| **All cause of death** | **Hakka culture region** | | | **Canton culture region** | | | **Hoklo culture region** | | | **P value** | |
| --- | --- | --- | --- | --- | --- | --- | --- | --- | --- | --- | --- |
|  | **2005** | **2015** | **Change** | **2005** | **2015** | **Change** | **2005** | **2015** | **Change** |  |  |
| All cause of death | 435.99 | 308.70 | -29.20^*^ | 338.41 | 256.71 | -24.14^*^ | 426.20 | 310.93 | -27.05^*^ | <0.001^#^ | |
| Communicable, maternal, neonatal, and nutritional diseases | 30.15 | 25.70 | -14.76^*^ | 21.15 | 18.68 | -11.68^*^ | 29.24 | 23.86 | -18.40^*^ | <0.001^#^ | |
| HIV/AIDS and tuberculosis | 5.33 | 4.19 | -21.39^*^ | 2.06 | 1.89 | -8.25 | 5.59 | 4.34 | -22.36^*^ | 0.426 | |
| Tuberculosis | 4.61 | 3.48 | -24.51^*^ | 1.55 | 1.13 | -27.10 | 4.93 | 3.63 | -26.37^*^ | 0.005^#^ | |
| HIV/AIDS | 0.72 | 0.71 | -1.39 | 0.51 | 0.76 | 49.02^*^ | 0.66 | 0.71 | 7.58 | 0.456 | |
| Diarrhea, lower respiratory and other common infectious diseases | 16.05 | 13.02 | -18.88^*^ | 12.34 | 9.75 | -20.99^*^ | 15.88 | 12.40 | -21.91^*^ | 0.145 | |
| Diarrheal diseases | 2.40 | 2.12 | -11.67 | 2.10 | 1.65 | -21.43^*^ | 2.16 | 1.83 | -15.28 | 0.773 | |
| Intestinal infectious diseases | 1.59 | 0.89 | -44.03 | 1.03 | 0.69 | -33.01^*^ | 1.72 | 0.93 | -45.93^*^ | <0.001^#^ | |
| Lower respiratory infections | 4.29 | 6.37 | 48.48^*^ | 3.97 | 5.23 | 31.74^*^ | 4.27 | 6.15 | 44.03 | 0.047^#^ | |
| Meningitis | 3.90 | 2.34 | -40.00^*^ | 2.60 | 1.47 | -43.46^*^ | 3.39 | 2.05 | -39.53^*^ | <0.001^#^ | |
| Measles | 2.85 | 0.65 | -77.19^*^ | 1.97 | 0.30 | -84.77^*^ | 3.30 | 0.82 | -75.15^*^ | <0.001^#^ | |
| Neglected tropical diseases and malaria | 0.73 | 0.10 | -86.30^*^ | 0.45 | 0.33 | -26.67 | 0.36 | 0.08 | -77.78 | 0.999 | |
| Malaria | 0.00 | 0.00 | 0.00 | 0.00 | 0.00 | 0.00 | 0.00 | 0.00 | 0.00 | - | |
| Rabies | 0.56 | 0.06 | -89.29 | 0.36 | 0.22 | -38.89 | 0.28 | 0.05 | -82.14 | 0.701 | |
| Intestinal nematode infections | 0.00 | 0.00 | 0.00 | 0.00 | 0.00 | 0.00 | 0.00 | 0.00 | 0.00 | - | |
| Maternal disorders | 0.45 | 0.12 | -73.33 ^*^ | 0.67 | 0.23 | -65.67^*^ | 0.42 | 0.11 | -73.81^*^ | 0.345 | |
| Neonatal disorders | 5.23 | 6.67 | 27.53^*^ | 3.85 | 5.10 | 32.47^*^ | 4.75 | 5.39 | 13.47^*^ | <0.001^#^ | |
| Nutritional deficiencies | 1.16 | 1.11 | -4.31^*^ | 1.01 | 0.97 | -3.96^*^ | 1.13 | 1.10 | -2.65^*^ | 0.004^#^ | |
| Iron-deficiency anemia | 0.01 | 0.01 | 0.00 | 0.01 | 0.01 | 0.00^*^ | 0.01 | 0.01 | 0.00 | 0.096 | |
| Other communicable, maternal, neonatal, and nutritional diseases | 1.20 | 0.50 | -58.33^*^ | 0.77 | 0.41 | -46.75 ^*^ | 1.10 | 0.43 | -60.91^*^ | 0.199 | |
| Sexually transmitted diseases excluding HIV | 0.40 | 0.15 | -62.50^*^ | 0.38 | 0.09 | -76.32^*^ | 0.31 | 0.12 | -61.29^*^ | 0.631 | |
| Hepatitis | 0.66 | 0.24 | -63.64^*^ | 0.30 | 0.23 | -23.33 | 0.66 | 0.22 | -66.67^*^ | 0.340 | |
| Acute hepatitis A | 0.34 | 0.07 | -79.41 | 0.13 | 0.03 | -76.92 | 0.34 | 0.07 | -79.41^*^ | 0.291 | |
| Acute hepatitis B | 0.22 | 0.12 | -45.45 | 0.11 | 0.15 | 36.36 | 0.22 | 0.10 | -54.55 | 0.597 | |
| Acute hepatitis C | 0.01 | 0.01 | 0.00 | 0.01 | 0.01 | 0.00 | 0.02 | 0.02 | 0.00 | 0.308 | |
| Non-communicable diseases | 372.82 | 264.90 | -28.95^*^ | 298.25 | 225.69 | -24.33^*^ | 368.12 | 270.23 | -26.59 | <0.001^#^ | |
| Neoplasms | 90.34 | 65.67 | -27.31^*^ | 86.96 | 66.51 | -23.52^*^ | 88.83 | 68.93 | -22.40 | 0.002^#^ | |
| esophageal cancer | 2.48 | 1.38 | -44.35^*^ | 1.80 | 0.90 | -50.00 ^*^ | 2.73 | 1.62 | -40.66^*^ | <0.001^#^ | |
| Stomach cancer | 7.23 | 5.87 | -18.81^*^ | 7.27 | 5.14 | -29.30^*^ | 7.05 | 6.31 | -10.50^*^ | 0.406 | |
| Liver cancer | 9.29 | 4.97 | -46.50^*^ | 7.84 | 4.01 | -48.85^*^ | 8.72 | 5.26 | -39.68^*^ | 0.001^#^ | |
| Tracheal, bronchus, and lung cancer | 24.74 | 12.14 | -50.93^*^ | 16.46 | 11.32 | -31.23^*^ | 25.28 | 12.67 | -49.88^*^ | 0.014^#^ | |
| Breast cancer | 9.52 | 8.33 | -12.50 | 11.09 | 8.67 | -21.82 | 8.87 | 8.71 | -1.80^*^ | <0.001^#^ | |
| Cervical cancer | 2.46 | 4.42 | 79.67^*^ | 4.07 | 6.15 | 51.11^*^ | 2.21 | 4.30 | 94.57 | 0.787 | |
| Uterine cancer | 3.47 | 1.65 | -52.45 | 2.69 | 1.09 | -59.48^*^ | 3.67 | 1.71 | -53.41^*^ | 0.398 | |
| Prostate cancer | 0.00 | 0.00 | 0.00 | 0.00 | 0.00 | 0.00 | 0.00 | 0.00 | 0.00 | - | |
| Colon and rectum cancer | 4.68 | 4.84 | 3.42 | 6.61 | 5.81 | -12.10^*^ | 4.55 | 5.21 | 14.51 | 0.001^#^ | |
| Nasopharyngeal cancer | 4.48 | 3.42 | -23.66 | 5.62 | 3.66 | -34.88^*^ | 4.29 | 3.65 | -14.92 | 0.045^#^ | |
| Pancreatic cancer | 1.58 | 2.07 | 31.01^*^ | 2.42 | 2.50 | 3.31^*^ | 1.54 | 2.13 | 38.31^*^ | 0.808 | |
| Ovarian cancer | 2.48 | 2.85 | 14.92^*^ | 3.86 | 3.64 | -5.70^*^ | 2.43 | 3.00 | 23.46 | 0.029^#^ | |
| Kidney cancer | 0.76 | 0.76 | 0.00 | 1.12 | 0.95 | -15.18 | 0.78 | 0.86 | 10.26^*^ | 0.015^#^ | |
| Bladder cancer | 1.37 | 0.63 | -54.01^*^ | 1.18 | 0.55 | -53.39^*^ | 1.39 | 0.70 | -49.64^*^ | 0.013^#^ | |
| Thyroid cancer | 0.97 | 0.73 | -24.74 | 1.17 | 0.76 | -35.04 | 0.88 | 0.73 | -17.05^*^ | 0.076 | |
| Leukemia | 4.09 | 2.88 | -29.58^*^ | 3.60 | 2.68 | -25.56^*^ | 3.93 | 2.97 | -24.43^*^ | 0.022^#^ | |
| Cardiovascular diseases | 162.02 | 121.00 | -25.32^*^ | 122.64 | 98.15 | -19.97^*^ | 160.00 | 124.36 | -22.28^*^ | <0.001^#^ | |
| Rheumatic heart disease | 7.09 | 3.22 | -54.58^*^ | 4.66 | 2.32 | -50.21^*^ | 6.99 | 3.33 | -52.36^*^ | 0.003^#^ | |
| Ischemic heart disease | 39.08 | 33.28 | -14.84^*^ | 35.97 | 35.77 | -0.56^*^ | 36.54 | 33.20 | -9.14^*^ | <0.001^#^ | |
| Cerebrovascular disease | 97.11 | 67.57 | -30.42^*^ | 65.17 | 42.41 | -34.92^*^ | 98.89 | 70.88 | -28.32^*^ | <0.001^#^ | |
| Ischemic stroke | 40.34 | 25.89 | -35.82 | 24.85 | 18.59 | -25.19^*^ | 40.67 | 29.17 | -28.28^*^ | <0.001^#^ | |
| Hemorrhagic stroke | 56.77 | 41.68 | -26.58^*^ | 40.32 | 23.82 | -40.92^*^ | 58.22 | 41.71 | -28.36^*^ | <0.001^#^ | |
| Hypertensive heart disease | 12.44 | 11.66 | -6.27^*^ | 12.26 | 13.54 | 10.44^*^ | 11.35 | 11.53 | 1.59^*^ | <0.001^#^ | |
| Cardiomyopathy and myocarditis | 1.16 | 0.89 | -23.28^*^ | 0.67 | 0.56 | -16.42 | 1.16 | 0.92 | -20.69 | 0.763 | |
| Atrial fibrillation and flutter | 1.26 | 1.41 | 11.90^*^ | 1.10 | 1.24 | 12.73^*^ | 1.24 | 1.44 | 16.13 | 0.149 | |
| Aortic aneurysm | 0.67 | 0.58 | -13.43 | 0.46 | 0.42 | -8.70 | 0.66 | 0.60 | -9.09 | 0.126 | |
| Chronic respiratory diseases | 63.68 | 29.93 | -53.00^*^ | 40.48 | 17.43 | -56.94^*^ | 63.79 | 29.31 | -54.05^*^ | <0.001^#^ | |
| Chronic obstructive pulmonary disease | 56.24 | 27.62 | -50.89^*^ | 37.21 | 16.78 | -54.90^*^ | 55.97 | 27.15 | -51.49^*^ | <0.001^#^ | |
| Asthma | 7.16 | 1.91 | -73.32^*^ | 3.13 | 0.46 | -85.30^*^ | 7.55 | 1.78 | -76.42^*^ | <0.001^#^ | |
| Cirrhosis | 6.51 | 3.59 | -44.85^*^ | 4.02 | 2.44 | -39.30^*^ | 6.68 | 3.90 | -41.62 | <0.001^#^ | |
| Cirrhosis due to alcohol use | 0.00 | 0.00 | 0.00 | 0.00 | 0.00 | 0.00 | 0.00 | 0.00 | 0.00 | - | |
| Digestive diseases | 6.71 | 3.90 | -41.88^*^ | 4.78 | 2.93 | -38.70^*^ | 6.49 | 3.88 | -40.22 | <0.001^#^ | |
| Peptic ulcer disease | 2.33 | 1.15 | -50.64^*^ | 1.40 | 0.68 | -51.43 | 2.19 | 1.09 | -50.23^*^ | 0.737 | |
| Pancreatitis | 0.71 | 0.45 | -36.62^*^ | 0.71 | 0.52 | -26.76^*^ | 0.75 | 0.52 | -30.67^*^ | 0.155 | |
| Neurological disorders | 13.91 | 17.74 | 27.53^*^ | 16.77 | 19.03 | 13.48^*^ | 13.81 | 17.12 | 23.97^*^ | 0.014^#^ | |
| Alzheimer’s disease and other dementias | 9.55 | 11.64 | 21.88^*^ | 11.19 | 11.69 | 4.47^*^ | 9.50 | 11.21 | 18.00^*^ | <0.001^#^ | |
| Parkinson’s disease | 2.34 | 3.72 | 58.97 | 3.38 | 5.19 | 53.55^*^ | 2.27 | 3.64 | 60.35^*^ | 0.017^#^ | |
| Epilepsy | 1.56 | 1.95 | 25.00^*^ | 1.74 | 1.76 | 1.15^*^ | 1.57 | 1.87 | 19.11^*^ | 0.208 | |
| hemicrania | 0.00 | 0.00 | 0.00 | 0.00 | 0.00 | 0.00 | 0.00 | 0.00 | 0.00 | - | |
| Mental and substance use disorders | 1.44 | 0.99 | -31.25 | 0.65 | 0.50 | -23.08 | 1.47 | 1.06 | -27.89 | 0.324 | |
| Schizophrenia | 0.40 | 0.40 | 0.00 | 0.29 | 0.29 | 0.00 | 0.43 | 0.46 | 6.98 | 0.945 | |
| Drug use disorders | 0.89 | 0.52 | -41.57 | 0.29 | 0.17 | -41.38^*^ | 0.89 | 0.52 | -41.57 | 0.008^#^ | |
| [Depressive](file:///D:\\Users\\nfernandez\\Program%20Files%20(x86)\\Youdao\\Dict\\7.5.2.0\\resultui\\dict\\%3fkeyword=depressive)[disorder](file:///D:\\Users\\nfernandez\\Program%20Files%20(x86)\\Youdao\\Dict\\7.5.2.0\\resultui\\dict\\%3fkeyword=disorder) | 0.00 | 0.00 | 0.00 | 0.00 | 0.00 | 0.00 | 0.00 | 0.00 | 0.00 | - | |
| [infantile](file:///D:\\Users\\nfernandez\\Program%20Files%20(x86)\\Youdao\\Dict\\7.5.2.0\\resultui\\dict\\%3fkeyword=infantile)[autism](file:///D:\\Users\\nfernandez\\Program%20Files%20(x86)\\Youdao\\Dict\\7.5.2.0\\resultui\\dict\\%3fkeyword=autism) | 0.00 | 0.00 | 0.00 | 0.00 | 0.00 | 0.00 | 0.00 | 0.00 | 0.00 | - | |
| Diabetes, urogenital, blood, and endocrine diseases | 22.20 | 15.61 | -29.68^*^ | 16.66 | 12.81 | -23.11^*^ | 21.33 | 15.65 | -26.63 | 0.001^#^ | |
| Diabetes mellitus | 8.86 | 4.60 | -48.08^*^ | 5.68 | 3.04 | -46.48^*^ | 8.73 | 4.89 | -43.99 ^*^ | <0.001^#^ | |
| Chronic kidney disease | 7.61 | 6.93 | -8.94 | 6.58 | 6.00 | -8.81^*^ | 6.99 | 6.67 | -4.58 | 0.039^#^ | |
| Gynecological diseases | 0.84 | 0.63 | -25.00^*^ | 0.63 | 0.68 | 7.94^*^ | 0.88 | 0.65 | -26.14^*^ | 0.102 | |
| Hemoglobinopathies and hemolytic anemia | 2.36 | 1.66 | -29.66 | 1.99 | 1.68 | -15.58^*^ | 2.31 | 1.68 | -27.27 | 0.051 | |
| Musculoskeletal disorders | 1.45 | 1.06 | -26.90^*^ | 1.66 | 1.27 | -23.49^*^ | 1.47 | 1.16 | -21.09 | 0.164 | |
| Rheumatoid arthritis | 0.51 | 0.39 | -23.53 | 0.58 | 0.47 | -18.97 | 0.53 | 0.43 | -18.87 | 0.516 | |
| [Osteoarthritis](file:///D:\\Users\\nfernandez\\Program%20Files%20(x86)\\Youdao\\Dict\\7.5.2.0\\resultui\\dict\\%3fkeyword=osteoarthritis) | 0.00 | 0.00 | 0.00 | 0.00 | 0.00 | 0.00 | 0.00 | 0.00 | 0.00 | - | |
| Low back and neck pain | 0.00 | 0.00 | 0.00 | 0.00 | 0.00 | 0.00 | 0.00 | 0.00 | 0.00 | - | |
| Low back pain | 0.00 | 0.00 | 0.00 | 0.00 | 0.00 | 0.00 | 0.00 | 0.00 | 0.00 | - | |
| Neck pain | 0.00 | 0.00 | 0.00 | 0.00 | 0.00 | 0.00 | 0.00 | 0.00 | 0.00 | - | |
| Other non-communicable diseases | 4.57 | 5.41 | 18.38^*^ | 3.62 | 4.62 | 27.62 | 4.26 | 4.86 | 14.08^*^ | 0.792 | |
| Congenital anomalies | 4.31 | 5.02 | 16.47^*^ | 3.41 | 4.34 | 27.27^*^ | 4.03 | 4.64 | 15.14^*^ | 0.045^#^ | |
| Skin and subcutaneous diseases | 0.22 | 0.33 | 50.00^*^ | 0.17 | 0.22 | 29.41 | 0.19 | 0.16 | -15.79 | 0.719 | |
| Sense organ diseases | 0.00 | 0.00 | 0.00 | 0.00 | 0.00 | 0.00 | 0.00 | 0.00 | 0.00 | - | |
| Oral disorders | 0.00 | 0.00 | 0.00 | 0.00 | 0.00 | 0.00 | 0.00 | 0.00 | 0.00 | - | |
| Injuries | 33.02 | 18.11 | -45.15^*^ | 19.01 | 12.33 | -35.14^*^ | 28.84 | 16.85 | -41.57^*^ | <0.001^#^ | |
| Transport injuries | 11.31 | 5.65 | -50.04^*^ | 6.44 | 3.76 | -41.61^*^ | 9.64 | 5.42 | -43.78^*^ | 0.283 | |
| Road injuries | 10.58 | 4.99 | -52.84^*^ | 6.08 | 3.34 | -45.07^*^ | 9.11 | 4.92 | -45.99^*^ | 0.502 | |
| Other transport injuries | 0.72 | 0.65 | -9.72 | 0.36 | 0.43 | 19.44 | 0.54 | 0.49 | -9.26 | 0.426 | |
| Unintentional injuries | 11.88 | 8.53 | -28.20^*^ | 8.04 | 6.28 | -21.89^*^ | 10.22 | 7.62 | -25.44^*^ | 0.005^#^ | |
| Falls | 1.95 | 2.13 | 9.23^*^ | 1.80 | 1.99 | 10.56^*^ | 1.63 | 1.89 | 15.95^*^ | 0.020^#^ | |
| Drowning | 3.97 | 2.45 | -38.29^*^ | 2.75 | 1.77 | -35.64^*^ | 3.41 | 2.25 | -34.02 | 0.496 | |
| Fire, heat, and hot substances | 1.06 | 0.77 | -27.36^*^ | 0.65 | 0.55 | -15.38 | 0.87 | 0.65 | -25.29^*^ | 0.961 | |
| Poisonings | 2.57 | 1.41 | -45.14^*^ | 1.36 | 0.77 | -43.38^*^ | 2.33 | 1.33 | -42.92^*^ | 0.024^#^ | |
| Intentional injury | 9.69 | 3.87 | -60.06^*^ | 4.44 | 2.24 | -49.55^*^ | 8.86 | 3.74 | -57.79 | <0.001^#^ | |
| Self-harm and interpersonal violence | 8.19 | 3.29 | -59.83^*^ | 3.78 | 1.87 | -50.53^*^ | 7.57 | 3.23 | -57.33^*^ | 0.749 | |
| Interpersonal violence | 1.51 | 0.58 | -61.59^*^ | 0.67 | 0.37 | -44.78^*^ | 1.29 | 0.52 | -59.69^*^ | <0.001^#^ | |
| Other diarrhea, lower respiratory and common infectious disease | 1.01 | 0.65 | -35.64 | 0.68 | 0.41 | -39.71^*^ | 1.05 | 0.62 | -40.95^*^ | 0.013^#^ | |
| Other neglected tropical disease | 0.16 | 0.04 | -75.00 | 0.09 | 0.11 | 22.22 | 0.09 | 0.03 | -66.67 | 0.011^#^ | |
| Other nutritional disease | 1.15 | 1.10 | -4.35^*^ | 1.00 | 0.96 | -4.00^*^ | 1.13 | 1.09 | -3.54^*^ | 0.001^#^ | |
| Other hepatitis | 0.09 | 0.03 | -66.67 | 0.05 | 0.05 | 0.00 | 0.09 | 0.03 | -66.67 | 0.645 | |
| Other Communicable, maternal, neonatal, and nutritional diseases | 0.14 | 0.10 | -28.57 | 0.09 | 0.08 | -11.11 | 0.13 | 0.09 | -30.77^*^ | 0.003^#^ |  |
| Other cancer | 10.75 | 8.73 | -18.79^*^ | 10.18 | 8.67 | -14.83^*^ | 10.52 | 9.09 | -13.59^*^ | <0.001^#^ |  |
| Other cardiovascular disease | 3.21 | 2.40 | -25.23^*^ | 2.34 | 1.90 | -18.80^*^ | 3.18 | 2.46 | -22.64 | 0.513 |  |
| Other chronic respiratory disease | 0.28 | 0.39 | 39.29^*^ | 0.14 | 0.20 | 42.86 | 0.27 | 0.38 | 40.74 | 0.465 |  |
| Other Cirrhosis | 6.51 | 3.59 | -44.85^*^ | 4.02 | 2.44 | -39.30 ^*^ | 6.68 | 3.90 | -41.62 | 0.001^#^ |  |
| Other digestive disease | 3.67 | 2.29 | -37.60 | 2.67 | 1.74 | -34.83^*^ | 3.54 | 2.27 | -35.88^*^ | 0.014^#^ |  |
| Other Neurological disorders | 0.46 | 0.42 | -8.70 | 0.46 | 0.38 | -17.39 | 0.46 | 0.41 | -10.87 | 0.096 |  |
| Other mental and substance use disorders | 0.14 | 0.08 | -42.86 | 0.07 | 0.04 | -42.86^*^ | 0.15 | 0.08 | -46.67 | 0.579 |  |
| Other diabetes, urogenital, blood, and endocrine diseases | 2.54 | 1.78 | -29.92 | 1.79 | 1.41 | -21.23^*^ | 2.42 | 1.77 | -26.86 | 0.704 |  |
| Other Musculoskeletal disorders | 0.94 | 0.67 | -28.72 | 1.08 | 0.80 | -25.93^*^ | 0.94 | 0.73 | -22.34 | 0.900 |  |
| Others | 0.04 | 0.07 | 75.00 | 0.03 | 0.06 | 100.00 | 0.04 | 0.06 | 50.00 | 0.301 |  |
| Other unintentional injury | 2.33 | 1.77 | -24.03^*^ | 1.48 | 1.20 | -18.92 | 1.97 | 1.50 | -23.86 | 0.071 |  |
| Other injury | 0.14 | 0.07 | -50.00 | 0.08 | 0.05 | -37.50 | 0.12 | 0.06 | -50.00 | 0.180 |  |

***: Statistical significance using the test of Cochran-Armitage trend for year 2005 to 2015 comparison (P< 0.05); #: statistical significance using poisson regression model**

**for comparison of different cultural regions (P< 0.05).**

**E-Table 3. Age-Standardized DALY rates for 116 diseases of male in Hakka, Canton and Hoklo cultural regions, between 2005 and 2015**

| **All cause of death** | **Hakka culture region** | | | **Canton culture region** | | | **Hoklo culture region** | | | **P value** |
| --- | --- | --- | --- | --- | --- | --- | --- | --- | --- | --- |
|  | **2005** | **2015** | **Change** | **2005** | **2015** | **Change** | **2005** | **2015** | **Change** |  |
| All cause of death | 30573.40 | 23887.80 | -21.87^*^ | 20290.30 | 16891.80 | -16.75^*^ | 29879.70 | 24591.90 | -17.70^*^ | <0.001^#^ |
| Communicable, maternal, neonatal, and nutritional diseases | 3192.65 | 3205.27 | 0.40^*^ | 2368.96 | 2078.34 | -12.27^*^ | 3286.82 | 3039.07 | -7.54^*^ | <0.001^#^ |
| HIV/AIDS and tuberculosis | 417.17 | 489.51 | 17.34^*^ | 302.39 | 261.93 | -13.38^*^ | 491.56 | 539.13 | 9.68^*^ | <0.001^#^ |
| Tuberculosis | 349.83 | 369.65 | 5.67^*^ | 210.29 | 149.51 | -28.90^*^ | 432.53 | 445.31 | 2.95^*^ | <0.001^#^ |
| HIV/AIDS | 67.35 | 119.86 | 77.97^*^ | 92.10 | 112.42 | 22.06^*^ | 59.03 | 93.81 | 58.92^*^ | <0.001^#^ |
| Diarrhea, lower respiratory and other common infectious diseases | 831.32 | 689.79 | -17.02^*^ | 670.32 | 518.13 | -22.70^*^ | 788.13 | 603.60 | -23.41^*^ | <0.001^#^ |
| Diarrheal diseases | 153.41 | 102.60 | -33.12^*^ | 97.17 | 58.34 | -39.96^*^ | 117.18 | 78.79 | -32.76^*^ | <0.001^#^ |
| Intestinal infectious diseases | 10.10 | 12.02 | 19.01^*^ | 16.32 | 12.06 | -26.10^*^ | 8.94 | 8.96 | 0.22^*^ | <0.001^#^ |
| Lower respiratory infections | 248.29 | 330.64 | 33.17^*^ | 219.94 | 253.57 | 15.29 | 231.82 | 288.79 | 24.58^*^ | <0.001^#^ |
| Meningitis | 149.91 | 77.86 | -48.06^*^ | 94.71 | 50.56 | -46.62^*^ | 127.47 | 62.53 | -50.95^*^ | <0.001^#^ |
| Measles | 136.35 | 48.78 | -64.22^*^ | 117.71 | 36.46 | -69.03^*^ | 166.12 | 54.58 | -67.14^*^ | <0.001^#^ |
| Neglected tropical diseases and malaria | 560.92 | 580.89 | 3.56^*^ | 449.05 | 301.75 | -32.80^*^ | 575.42 | 524.67 | -8.82^*^ | <0.001^#^ |
| Malaria | 3.43 | 2.03 | -40.82^*^ | 3.48 | 1.24 | -64.37^*^ | 3.26 | 1.71 | -47.55^*^ | 0.001^#^ |
| Rabies | 29.39 | 10.24 | -65.16^*^ | 29.68 | 10.02 | -66.24^*^ | 27.24 | 8.65 | -68.25^*^ | <0.001^#^ |
| Intestinal nematode infections | 376.36 | 436.70 | 16.03^*^ | 287.27 | 188.64 | -34.33^*^ | 375.90 | 373.21 | -0.72^*^ | <0.001^#^ |
| Maternal disorders | 0.00 | 0.00 | 0.00 | 0.00 | 0.00 | 0.00 | 0.00 | 0.00 | 0.00 | - |
| Neonatal disorders | 653.23 | 748.65 | 14.61^*^ | 519.59 | 612.48 | 17.88^*^ | 644.81 | 669.26 | 3.79^*^ | <0.001^#^ |
| Nutritional deficiencies | 563.86 | 587.28 | 4.15^*^ | 350.64 | 344.77 | -1.67^*^ | 592.43 | 582.02 | -1.76^*^ | <0.001^#^ |
| Iron-deficiency anemia | 520.41 | 535.23 | 2.85^*^ | 319.49 | 310.87 | -2.70^*^ | 544.38 | 528.82 | -2.86^*^ | <0.001^#^ |
| Other communicable, maternal, neonatal, and nutritional diseases | 166.14 | 109.15 | -34.30 ^*^ | 76.97 | 39.29 | -48.95^*^ | 194.46 | 120.39 | -38.09^*^ | <0.001^#^ |
| Sexually transmitted diseases excluding HIV | 59.43 | 38.62 | -35.02^*^ | 39.85 | 15.97 | -59.92^*^ | 72.28 | 46.29 | -35.96^*^ | <0.001^#^ |
| Hepatitis | 70.45 | 32.59 | -53.74^*^ | 27.64 | 13.16 | -52.39^*^ | 83.92 | 39.40 | -53.05^*^ | <0.001^#^ |
| Acute hepatitis A | 11.80 | 15.26 | 29.32^*^ | 9.66 | 8.21 | -15.01^*^ | 11.81 | 15.25 | 29.13^*^ | 0.123 |
| Acute hepatitis B | 43.76 | 11.63 | -73.42^*^ | 11.13 | 2.44 | -78.08^*^ | 55.48 | 17.09 | -69.20^*^ | <0.001^#^ |
| Acute hepatitis C | 5.99 | 2.11 | -64.77^*^ | 3.13 | 0.81 | -74.12^*^ | 6.11 | 2.61 | -57.28^*^ | <0.001^#^ |
| Non-communicable diseases | 22797.60 | 18238.10 | -20.00^*^ | 15472.20 | 13365.30 | -13.62^*^ | 22407.10 | 19184.50 | -14.38^*^ | <0.001^#^ |
| Neoplasms | 5346.63 | 4057.13 | -24.12^*^ | 4092.09 | 3170.22 | -22.53^*^ | 5383.42 | 4410.85 | -18.07^*^ | <0.001^#^ |
| esophageal cancer | 310.93 | 240.78 | -22.56^*^ | 250.44 | 185.14 | -26.07^*^ | 335.66 | 289.75 | -13.68^*^ | <0.001^#^ |
| Stomach cancer | 627.87 | 473.59 | -24.57 | 445.46 | 315.47 | -29.18^*^ | 640.71 | 526.08 | -17.89^*^ | <0.001^#^ |
| Liver cancer | 1353.35 | 971.73 | -28.20^*^ | 982.48 | 699.63 | -28.79^*^ | 1283.27 | 1009.36 | -21.34^*^ | <0.001^#^ |
| Tracheal, bronchus, and lung cancer | 1267.16 | 932.95 | -26.37^*^ | 863.34 | 726.81 | -15.81^*^ | 1287.84 | 989.02 | -23.20^*^ | <0.001^#^ |
| Breast cancer | 23.43 | 13.06 | -44.26^*^ | 16.87 | 8.82 | -47.72^*^ | 23.33 | 13.98 | -40.08^*^ | <0.001^#^ |
| Cervical cancer | 0.00 | 0.00 | 0.00 | 0.00 | 0.00 | 0.00 | 0.00 | 0.00 | 0.00 | - |
| Uterine cancer | 0.00 | 0.00 | 0.00 | 0.00 | 0.00 | 0.00 | 0.00 | 0.00 | 0.00 | - |
| Prostate cancer | 17.43 | 13.90 | -20.25^*^ | 16.89 | 12.84 | -23.98^*^ | 17.85 | 15.78 | -11.60^*^ | <0.001^#^ |
| Colon and rectum cancer | 199.50 | 222.10 | 11.33^*^ | 213.68 | 233.01 | 9.05^*^ | 211.78 | 259.85 | 22.70^*^ | <0.001^#^ |
| Nasopharyngeal cancer | 519.88 | 331.08 | -36.32^*^ | 436.87 | 272.86 | -37.54^*^ | 546.74 | 384.93 | -29.60^*^ | <0.001^#^ |
| Pancreatic cancer | 85.27 | 90.88 | 6.58^*^ | 92.27 | 83.12 | -9.92^*^ | 91.35 | 101.92 | 11.57^*^ | <0.001^#^ |
| Ovarian cancer | 0.00 | 0.00 | 0.00 | 0.00 | 0.00 | 0.00 | 0.00 | 0.00 | 0.00 | - |
| Kidney cancer | 27.75 | 25.95 | -6.49^*^ | 33.54 | 28.29 | -15.65 | 30.34 | 30.75 | 1.35 | <0.001^#^ |
| Bladder cancer | 39.40 | 24.23 | -38.50^*^ | 34.53 | 19.69 | -42.98^*^ | 44.88 | 30.76 | -31.46^*^ | <0.001^#^ |
| Thyroid cancer | 19.37 | 13.78 | -28.86^*^ | 19.06 | 12.53 | -34.26^*^ | 17.35 | 13.36 | -23.00^*^ | 0.028^#^ |
| Leukemia | 265.21 | 191.29 | -27.87^*^ | 228.59 | 169.60 | -25.81^*^ | 265.07 | 201.71 | -23.90^*^ | <0.001^#^ |
| Cardiovascular diseases | 5392.89 | 4114.96 | -23.70^*^ | 3848.92 | 3176.32 | -17.48^*^ | 5300.27 | 4324.13 | -18.42^*^ | <0.001^#^ |
| Rheumatic heart disease | 156.26 | 85.91 | -45.02^*^ | 91.26 | 55.58 | -39.10^*^ | 151.75 | 88.77 | -41.50^*^ | <0.001^#^ |
| Ischemic heart disease | 1979.38 | 1732.39 | -12.48^*^ | 1722.07 | 1564.28 | -9.16^*^ | 1912.52 | 1788.90 | -6.46^*^ | <0.001^#^ |
| Cerebrovascular disease | 2830.40 | 1853.78 | -34.50^*^ | 1723.04 | 1199.55 | -30.38^*^ | 2832.80 | 2015.08 | -28.87^*^ | <0.001^#^ |
| Ischemic stroke | 1158.38 | 733.96 | -36.64^*^ | 738.77 | 503.56 | -31.84^*^ | 1162.30 | 817.92 | -29.63^*^ | <0.001^#^ |
| Hemorrhagic stroke | 1672.02 | 1119.81 | -33.03^*^ | 984.28 | 695.99 | -29.29^*^ | 1670.50 | 1197.16 | -28.34^*^ | <0.001^#^ |
| Hypertensive heart disease | 142.27 | 191.42 | 34.55^*^ | 133.34 | 182.10 | 36.57^*^ | 128.54 | 176.73 | 37.49^*^ | <0.001^#^ |
| Cardiomyopathy and myocarditis | 87.12 | 72.75 | -16.49^*^ | 42.59 | 38.21 | -10.28^*^ | 82.27 | 69.95 | -14.98^*^ | 0.126 |
| Atrial fibrillation and flutter | 53.87 | 63.82 | 18.47^*^ | 44.94 | 56.45 | 25.61^*^ | 53.11 | 67.21 | 26.55^*^ | <0.001^#^ |
| Aortic aneurysm | 44.86 | 39.91 | -11.03 | 28.43 | 28.08 | -1.23 | 43.91 | 42.57 | -3.05^*^ | 0.003^#^ |
| Chronic respiratory diseases | 2093.33 | 1402.90 | -32.98^*^ | 1235.25 | 835.13 | -32.39^*^ | 2163.80 | 1503.20 | -30.53^*^ | <0.001^#^ |
| Chronic obstructive pulmonary disease | 1935.21 | 1294.99 | -33.08^*^ | 1170.76 | 782.81 | -33.14^*^ | 2002.05 | 1400.70 | -30.04^*^ | <0.001^#^ |
| Asthma | 135.88 | 75.38 | -44.52^*^ | 54.56 | 35.58 | -34.79^*^ | 140.39 | 71.11 | -49.35^*^ | <0.001^#^ |
| Cirrhosis | 692.94 | 413.87 | -40.27^*^ | 359.79 | 217.12 | -39.65^*^ | 726.63 | 446.89 | -38.50^*^ | <0.001^#^ |
| Cirrhosis due to alcohol use | 1.07 | 1.19 | 11.21 | 0.87 | 0.97 | 11.49 | 1.04 | 1.20 | 15.38 | 0.962 |
| Digestive diseases | 474.84 | 337.33 | -28.96^*^ | 236.08 | 176.66 | -25.17^*^ | 469.89 | 360.25 | -23.33^*^ | <0.001^#^ |
| Peptic ulcer disease | 180.95 | 100.79 | -44.30^*^ | 82.77 | 48.22 | -41.74^*^ | 176.84 | 105.51 | -40.34^*^ | <0.001^#^ |
| Pancreatitis | 28.52 | 15.77 | -44.71^*^ | 18.99 | 10.55 | -44.44^*^ | 31.52 | 18.80 | -40.36^*^ | <0.001^#^ |
| Neurological disorders | 669.82 | 559.38 | -16.49^*^ | 611.38 | 587.29 | -3.94^*^ | 661.11 | 609.89 | -7.75 | <0.001^#^ |
| Alzheimer’s disease and other dementias | 156.89 | 171.80 | 9.50^*^ | 167.54 | 192.46 | 14.87^*^ | 158.02 | 183.30 | 16.00^*^ | <0.001^#^ |
| Parkinson’s disease | 99.34 | 61.35 | -38.24^*^ | 93.81 | 61.87 | -34.05^*^ | 98.74 | 66.00 | -33.16^*^ | 0.016^#^ |
| Epilepsy | 59.75 | 66.62 | 11.50^*^ | 59.30 | 77.10 | 30.02^*^ | 58.16 | 70.66 | 21.49^*^ | 0.968 |
| hemicrania | 239.00 | 173.55 | -27.38^*^ | 190.43 | 166.40 | -12.62^*^ | 232.73 | 193.67 | -16.78^*^ | <0.001^#^ |
| Mental and substance use disorders | 2952.68 | 2403.87 | -18.59^*^ | 1175.39 | 1114.26 | -5.20^*^ | 2662.12 | 2309.69 | -13.24^*^ | <0.001^#^ |
| Schizophrenia | 428.76 | 634.96 | 48.09^*^ | 130.04 | 236.03 | 81.51^*^ | 329.48 | 591.18 | 79.43^*^ | <0.001^#^ |
| Drug use disorders | 598.26 | 262.73 | -56.08^*^ | 91.14 | 46.26 | -49.24^*^ | 460.75 | 211.85 | -54.02^*^ | <0.001^#^ |
| [Depressive](file:///D:\\Users\\nfernandez\\Program%20Files%20(x86)\\Youdao\\Dict\\7.5.2.0\\resultui\\dict\\%3fkeyword=depressive)[disorder](file:///D:\\Users\\nfernandez\\Program%20Files%20(x86)\\Youdao\\Dict\\7.5.2.0\\resultui\\dict\\%3fkeyword=disorder) | 585.94 | 491.45 | -16.13 | 287.60 | 273.65 | -4.85^*^ | 590.63 | 514.44 | -12.90^*^ | <0.001^#^ |
| [infantile](file:///D:\\Users\\nfernandez\\Program%20Files%20(x86)\\Youdao\\Dict\\7.5.2.0\\resultui\\dict\\%3fkeyword=infantile)[autism](file:///D:\\Users\\nfernandez\\Program%20Files%20(x86)\\Youdao\\Dict\\7.5.2.0\\resultui\\dict\\%3fkeyword=autism) | 251.52 | 197.89 | -21.32^*^ | 121.55 | 110.41 | -9.16^*^ | 231.89 | 187.34 | -19.21^*^ | <0.001^#^ |
| Diabetes, urogenital, blood, and endocrine diseases | 1673.29 | 1360.14 | -18.71^*^ | 1139.19 | 1022.40 | -10.25^*^ | 1627.86 | 1440.89 | -11.49^*^ | <0.001^#^ |
| Diabetes mellitus | 718.68 | 623.94 | -13.18^*^ | 471.87 | 435.31 | -7.75^*^ | 714.43 | 683.69 | -4.30^*^ | <0.001^#^ |
| Chronic kidney disease | 432.48 | 317.99 | -26.47^*^ | 282.27 | 217.67 | -22.89^*^ | 403.56 | 324.98 | -19.47^*^ | <0.001^#^ |
| Gynecological diseases | 0.00 | 0.00 | 0.00 | 0.00 | 0.00 | 0.00 | 0.00 | 0.00 | 0.00 | - |
| Hemoglobinopathies and hemolytic anemia | 285.26 | 206.32 | -27.67^*^ | 175.79 | 163.16 | -7.18^*^ | 279.83 | 218.30 | -21.99^*^ | 0.020^#^ |
| Musculoskeletal disorders | 1432.35 | 1413.85 | -1.29^*^ | 1065.00 | 1074.25 | 0.87^*^ | 1413.48 | 1541.20 | 9.04^*^ | <0.001^#^ |
| Rheumatoid arthritis | 39.06 | 46.91 | 20.10^*^ | 23.52 | 26.20 | 11.39 | 39.23 | 54.51 | 38.95^*^ | <0.001^#^ |
| [Osteoarthritis](file:///D:\\Users\\nfernandez\\Program%20Files%20(x86)\\Youdao\\Dict\\7.5.2.0\\resultui\\dict\\%3fkeyword=osteoarthritis) | 116.72 | 149.21 | 27.84^*^ | 78.97 | 101.83 | 28.95^*^ | 121.65 | 168.16 | 38.23^*^ | 0.263 |
| Low back and neck pain | 986.10 | 939.65 | -4.71^*^ | 868.99 | 860.95 | -0.93^*^ | 1049.51 | 1118.97 | 6.62^*^ | <0.001^#^ |
| Low back pain | 530.03 | 505.34 | -4.66^*^ | 475.25 | 474.37 | -0.19^*^ | 561.29 | 597.09 | 6.38^*^ | <0.001^#^ |
| Neck pain | 456.07 | 434.31 | -4.77^*^ | 393.74 | 386.58 | -1.82^*^ | 488.23 | 521.88 | 6.89^*^ | <0.001^#^ |
| Other non-communicable diseases | 2068.83 | 2174.67 | 5.12^*^ | 1709.14 | 1991.67 | 16.53^*^ | 1998.53 | 2237.47 | 11.96^*^ | <0.001^#^ |
| Congenital anomalies | 588.58 | 715.84 | 21.62^*^ | 431.78 | 633.10 | 46.63^*^ | 534.55 | 683.47 | 27.86^*^ | <0.001^#^ |
| Skin and subcutaneous diseases | 468.87 | 397.11 | -15.30^*^ | 285.99 | 238.39 | -16.64^*^ | 403.05 | 349.42 | -13.31^*^ | <0.001^#^ |
| Sense organ diseases | 824.45 | 880.06 | 6.75^*^ | 740.57 | 854.02 | 15.32^*^ | 881.10 | 1013.31 | 15.01^*^ | <0.001^#^ |
| Oral disorders | 182.80 | 175.58 | -3.95^*^ | 130.89 | 143.71 | 9.79^*^ | 176.29 | 186.34 | 5.70^*^ | <0.001^#^ |
| Injuries | 4583.13 | 2444.38 | -46.67^*^ | 2449.12 | 1448.12 | -40.87^*^ | 4185.79 | 2368.37 | -43.42^*^ | <0.001^#^ |
| Transport injuries | 2105.63 | 1032.07 | -50.99^*^ | 1096.99 | 592.88 | -45.95^*^ | 1890.80 | 1033.08 | -45.36^*^ | <0.001^#^ |
| Road injuries | 1942.69 | 992.31 | -48.92^*^ | 1064.13 | 581.66 | -45.34^*^ | 1757.34 | 1001.65 | -43.00^*^ | <0.001^#^ |
| Other transport injuries | 162.94 | 39.76 | -75.60^*^ | 32.85 | 11.22 | -65.84^*^ | 133.46 | 31.43 | -76.45^*^ | <0.001^#^ |
| Unintentional injuries | 1693.22 | 1074.31 | -36.55^*^ | 1082.76 | 715.47 | -33.92^*^ | 1504.34 | 983.46 | -34.63^*^ | <0.001^#^ |
| Falls | 551.72 | 410.26 | -25.64^*^ | 351.37 | 276.49 | -21.31^*^ | 491.76 | 385.16 | -21.68^*^ | <0.001^#^ |
| Drowning | 391.39 | 205.94 | -47.38^*^ | 285.99 | 147.42 | -48.45^*^ | 346.27 | 180.80 | -47.79^*^ | <0.001^#^ |
| Fire, heat, and hot substances | 106.34 | 37.26 | -64.96^*^ | 45.47 | 16.95 | -62.72^*^ | 94.66 | 33.03 | -65.11^*^ | <0.001^#^ |
| Poisonings | 113.87 | 55.28 | -51.45^*^ | 54.07 | 28.49 | -47.31^*^ | 97.98 | 48.61 | -50.39^*^ | <0.001^#^ |
| Intentional injury | 763.90 | 330.91 | -56.68^*^ | 258.26 | 135.28 | -47.62^*^ | 771.95 | 344.94 | -55.32^*^ | 0.060 |
| Self-harm and interpersonal violence | 472.86 | 212.93 | -54.97^*^ | 166.61 | 91.62 | -45.01^*^ | 473.74 | 223.31 | -52.86^*^ | <0.001^#^ |
| Interpersonal violence | 291.04 | 117.99 | -59.46^*^ | 91.65 | 43.66 | -52.36^*^ | 298.21 | 121.62 | -59.22^*^ | <0.001^#^ |
| Other diarrhea, lower respiratory and common infectious disease | 133.26 | 117.89 | -11.53^*^ | 124.46 | 107.14 | -13.92^*^ | 136.61 | 109.95 | -19.52^*^ | 0.038^#^ |
| Other neglected tropical disease | 151.74 | 131.92 | -13.06^*^ | 128.63 | 101.86 | -20.81^*^ | 169.02 | 141.09 | -16.52^*^ | <0.001^#^ |
| Other nutritional disease | 43.45 | 52.04 | 19.77^*^ | 31.16 | 33.90 | 8.79 | 48.05 | 53.20 | 10.72^*^ | <0.001^#^ |
| Other hepatitis | 8.90 | 3.60 | -59.55^*^ | 3.72 | 1.70 | -54.30^*^ | 10.52 | 4.45 | -57.70^*^ | <0.001^#^ |
| Other Communicable, maternal, neonatal, and nutritional diseases | 36.27 | 37.93 | 4.58^*^ | 9.47 | 10.16 | 7.29 | 38.26 | 34.70 | -9.30^*^ | <0.001^#^ |
| Other cancer | 590.09 | 511.81 | -13.27^*^ | 458.07 | 402.39 | -12.16^*^ | 587.24 | 543.58 | -7.43^*^ | <0.001^#^ |
| Other cardiovascular disease | 98.73 | 74.99 | -24.05^*^ | 63.25 | 52.06 | -17.69^*^ | 95.36 | 74.92 | -21.43^*^ | <0.001^#^ |
| Other chronic respiratory disease | 22.24 | 32.53 | 46.27^*^ | 9.93 | 16.73 | 68.48^*^ | 21.36 | 31.40 | 47.00^*^ | <0.001^#^ |
| Other Cirrhosis | 691.87 | 412.68 | -40.35^*^ | 358.92 | 216.15 | -39.78^*^ | 725.59 | 445.70 | -38.57^*^ | <0.001^#^ |
| Other digestive disease | 265.37 | 220.78 | -16.80^*^ | 134.32 | 117.89 | -12.23^*^ | 261.53 | 235.94 | -9.78^*^ | <0.001^#^ |
| Other Neurological disorders | 114.84 | 86.05 | -25.07^*^ | 100.32 | 89.47 | -10.82^*^ | 113.46 | 96.25 | -15.17^*^ | <0.001^#^ |
| Other mental and substance use disorders | 1088.19 | 816.84 | -24.94^*^ | 545.05 | 447.90 | -17.82^*^ | 1049.37 | 804.88 | -23.30^*^ | <0.001^#^ |
| Other diabetes, urogenital, blood, and endocrine diseases | 236.88 | 211.90 | -10.55^*^ | 209.26 | 206.26 | -1.43^*^ | 230.04 | 213.92 | -7.01^*^ | <0.001^#^ |
| Other Musculoskeletal disorders | 290.48 | 278.09 | -4.27^*^ | 93.52 | 85.27 | -8.82^*^ | 203.09 | 199.56 | -1.74^*^ | <0.001^#^ |
| Others | 4.13 | 6.07 | 46.97 | 119.91 | 122.46 | 2.13^*^ | 3.53 | 4.94 | 39.94^*^ | <0.001^#^ |
| Other unintentional injury | 529.88 | 365.57 | -31.01^*^ | 345.87 | 246.12 | -28.84^*^ | 473.68 | 335.86 | -29.10^*^ | 0.748 |
| Other injury | 20.38 | 7.08 | -65.26^*^ | 11.11 | 4.48 | -59.68^*^ | 18.70 | 6.89 | -63.16^*^ | <0.001^#^ |

***: Statistical significance using the test of Cochran-Armitage trend for year 2005 to 2015 comparison (P< 0.05); #: statistical significance using Poisson regression model**

**For comparison of different cultural regions (P< 0.05).**

**E-Table 4. Age-Standardized DALY rates for 116 diseases of female in Hakka, Canton and Hoklo cultural regions, between 2005 and 2015**

| **All cause of death** | **Hakka culture region** | | | **Canton culture region** | | | **Hoklo culture region** | | | **P value** |
| --- | --- | --- | --- | --- | --- | --- | --- | --- | --- | --- |
|  | **2005** | **2015** | **Change** | **2005** | **2015** | **Change** | **2005** | **2015** | **Change** |  |
| All cause of death | 20955.20 | 16033.10 | -23.49^*^ | 13875.60 | 11854.90 | -14.56^*^ | 19896.70 | 16078.50 | -19.19^*^ | <0.001^#^ |
| Communicable, maternal, neonatal, and nutritional diseases | 2369.39 | 2103.79 | -11.21^*^ | 1567.16 | 1490.74 | -4.88^*^ | 2274.61 | 1945.24 | -14.48^*^ | <0.001^#^ |
| HIV/AIDS and tuberculosis | 244.99 | 225.72 | -7.87^*^ | 82.61 | 89.53 | 8.38^*^ | 275.13 | 228.80 | -16.84^*^ | <0.001^#^ |
| Tuberculosis | 226.13 | 202.44 | -10.48^*^ | 68.40 | 60.78 | -11.14^*^ | 257.65 | 205.47 | -20.25^*^ | <0.001^#^ |
| HIV/AIDS | 18.85 | 23.27 | 23.45 ^*^ | 14.22 | 28.75 | 102.18^*^ | 17.48 | 23.33 | 33.47^*^ | 0.322 |
| Diarrhea, lower respiratory and other common infectious diseases | 585.71 | 503.70 | -14.00^*^ | 376.76 | 289.67 | -23.12^*^ | 644.37 | 481.67 | -25.25^*^ | <0.001^#^ |
| Diarrheal diseases | 44.77 | 76.88 | 71.72^*^ | 33.60 | 44.38 | 32.08^*^ | 38.40 | 62.70 | 63.28^*^ | <0.001^#^ |
| Intestinal infectious diseases | 15.27 | 7.76 | -49.18^*^ | 9.34 | 8.01 | -14.24^*^ | 15.90 | 7.44 | -53.21^*^ | <0.001^#^ |
| Lower respiratory infections | 77.20 | 188.14 | 143.70^*^ | 62.58 | 126.53 | 102.19^*^ | 72.71 | 168.72 | 132.05^*^ | <0.001^#^ |
| Meningitis | 85.26 | 65.28 | -23.43^*^ | 46.66 | 32.23 | -30.93^*^ | 71.93 | 55.53 | -22.80^*^ | <0.001^#^ |
| Measles | 213.83 | 54.26 | -74.62^*^ | 137.72 | 23.38 | -83.02^*^ | 246.40 | 68.00 | -72.40^*^ | <0.001^#^ |
| Neglected tropical diseases and malaria | 478.39 | 211.36 | -55.82^*^ | 210.01 | 236.79 | 12.75^*^ | 255.22 | 175.09 | -31.40^*^ | <0.001^#^ |
| Malaria | 0.00 | 0.00 | 0.00 | 0.00 | 0.00 | 0.00 | 0.00 | 0.00 | 0.00 | - |
| Rabies | 27.77 | 2.72 | -90.21^*^ | 22.07 | 14.70 | -33.39^*^ | 12.17 | 2.32 | -80.94^*^ | <0.001^#^ |
| Intestinal nematode infections | 289.16 | 154.93 | -46.42^*^ | 98.74 | 112.98 | 14.42^*^ | 135.40 | 106.66 | -21.23^*^ | <0.001^#^ |
| Maternal disorders | 29.82 | 10.93 | -63.35^*^ | 44.96 | 16.79 | -62.66^*^ | 29.03 | 10.50 | -63.83^*^ | <0.001^#^ |
| Neonatal disorders | 571.45 | 713.69 | 24.89^*^ | 458.46 | 535.03 | 16.70^*^ | 595.50 | 635.41 | 6.70 | <0.001^#^ |
| Nutritional deficiencies | 378.49 | 386.32 | 2.07^*^ | 333.53 | 281.40 | -15.63^*^ | 398.40 | 370.02 | -7.12 | <0.001^#^ |
| Iron-deficiency anemia | 316.95 | 329.35 | 3.91^*^ | 276.50 | 230.69 | -16.57^*^ | 340.52 | 317.38 | -6.80^*^ | <0.001^#^ |
| Other communicable, maternal, neonatal, and nutritional diseases | 80.54 | 52.07 | -35.35 ^*^ | 60.82 | 41.51 | -31.75^*^ | 76.97 | 43.75 | -43.16^*^ | <0.001^#^ |
| Sexually transmitted diseases excluding HIV | 44.45 | 24.40 | -45.11^*^ | 37.02 | 10.19 | -72.47 ^*^ | 40.45 | 20.68 | -48.88^*^ | 0.006^#^ |
| Hepatitis | 23.66 | 16.41 | -30.64^*^ | 12.48 | 20.07 | 60.82^*^ | 24.91 | 14.27 | -42.71^*^ | 0.016^#^ |
| Acute hepatitis A | 12.04 | 4.87 | -59.55^*^ | 4.46 | 1.66 | -62.78^*^ | 12.92 | 5.45 | -57.82^*^ | <0.001^#^ |
| Acute hepatitis B | 8.66 | 9.16 | 5.77 | 5.58 | 14.37 | 157.53^*^ | 8.84 | 6.62 | -25.11^*^ | <0.001^#^ |
| Acute hepatitis C | 0.26 | 0.40 | 53.85 | 0.30 | 0.32 | 6.67 | 0.37 | 0.51 | 37.84 | <0.001^#^ |
| Non-communicable diseases | 16815.90 | 12930.50 | -23.11^*^ | 11404.70 | 9746.11 | -14.54^*^ | 16130.10 | 13232.30 | -17.97^*^ | <0.001^#^ |
| Neoplasms | 2555.60 | 1706.01 | -33.24 | 2297.98 | 1619.35 | -29.53^*^ | 2492.20 | 1796.17 | -27.93^*^ | <0.001^#^ |
| esophageal cancer | 56.79 | 26.83 | -52.76^*^ | 36.23 | 13.87 | -61.72^*^ | 64.16 | 32.60 | -49.19^*^ | <0.001^#^ |
| Stomach cancer | 184.00 | 140.38 | -23.71 ^*^ | 177.02 | 113.26 | -36.02^*^ | 180.26 | 153.00 | -15.12^*^ | <0.001^#^ |
| Liver cancer | 245.54 | 120.85 | -50.78^*^ | 199.21 | 89.51 | -55.07^*^ | 230.16 | 129.28 | -43.83 ^*^ | <0.001^#^ |
| Tracheal, bronchus, and lung cancer | 721.18 | 313.72 | -56.50^*^ | 448.54 | 280.85 | -37.39^*^ | 717.81 | 329.24 | -54.13^*^ | <0.001^#^ |
| Breast cancer | 271.89 | 233.10 | -14.27^*^ | 310.84 | 235.52 | -24.23^*^ | 253.96 | 246.62 | -2.89^*^ | <0.001^#^ |
| Cervical cancer | 58.07 | 106.40 | 83.23^*^ | 96.67 | 146.40 | 51.44^*^ | 52.38 | 104.25 | 99.03^*^ | <0.001^#^ |
| Uterine cancer | 88.38 | 36.48 | -58.72^*^ | 61.94 | 19.64 | -68.29^*^ | 94.62 | 38.58 | -59.23^*^ | <0.001^#^ |
| Prostate cancer | 0.00 | 0.00 | 0.00 | 0.00 | 0.00 | 0.00^*^ | 0.00 | 0.00 | 0.00 | - |
| Colon and rectum cancer | 116.28 | 116.26 | -0.02^*^ | 162.80 | 135.17 | -16.97^*^ | 114.13 | 127.07 | 11.34^*^ | 0.015^#^ |
| Nasopharyngeal cancer | 112.14 | 79.67 | -28.95 ^*^ | 137.52 | 80.67 | -41.34^*^ | 108.14 | 86.53 | -19.98^*^ | <0.001^#^ |
| Pancreatic cancer | 33.78 | 43.40 | 28.48^*^ | 51.42 | 49.79 | -3.17^*^ | 33.38 | 45.27 | 35.62^*^ | 0.567 |
| Ovarian cancer | 57.90 | 64.49 | 11.38^*^ | 90.13 | 79.77 | -11.49 | 57.59 | 69.03 | 19.86^*^ | <0.001^#^ |
| Kidney cancer | 14.40 | 13.12 | -8.89^*^ | 20.71 | 15.30 | -26.12^*^ | 15.10 | 15.39 | 1.92^*^ | <0.001^#^ |
| Bladder cancer | 30.43 | 10.39 | -65.86^*^ | 22.89 | 7.54 | -67.06^*^ | 31.52 | 12.15 | -61.45^*^ | <0.001^#^ |
| Thyroid cancer | 21.15 | 13.44 | -36.45^*^ | 23.45 | 12.41 | -47.08^*^ | 19.23 | 13.51 | -29.75^*^ | 0.374 |
| Leukemia | 192.62 | 124.08 | -35.58^*^ | 158.49 | 106.60 | -32.74^*^ | 183.20 | 125.20 | -31.66^*^ | <0.001^#^ |
| Cardiovascular diseases | 3229.66 | 2078.86 | -35.63^*^ | 2028.24 | 1414.56 | -30.26^*^ | 3169.06 | 2145.14 | -32.31^*^ | <0.001^#^ |
| Rheumatic heart disease | 237.74 | 101.84 | -57.16^*^ | 117.32 | 56.85 | -51.54^*^ | 232.38 | 106.21 | -54.29^*^ | <0.001^#^ |
| Ischemic heart disease | 818.69 | 596.08 | -27.19^*^ | 643.48 | 530.81 | -17.51^*^ | 761.65 | 591.92 | -22.28^*^ | <0.001^#^ |
| Cerebrovascular disease | 1796.17 | 1099.84 | -38.77^*^ | 1017.47 | 612.76 | -39.78^*^ | 1819.29 | 1164.56 | -35.99^*^ | <0.001^#^ |
| Ischemic stroke | 756.64 | 460.44 | -39.15^*^ | 387.17 | 284.30 | -26.57^*^ | 761.85 | 523.85 | -31.24^*^ | <0.001^#^ |
| Hemorrhagic stroke | 1039.52 | 639.41 | -38.49^*^ | 630.30 | 328.46 | -47.89^*^ | 1057.44 | 640.70 | -39.41^*^ | <0.001^#^ |
| Hypertensive heart disease | 201.03 | 136.68 | -32.01^*^ | 153.31 | 126.36 | -17.58^*^ | 181.53 | 134.40 | -25.96^*^ | <0.001^#^ |
| Cardiomyopathy and myocarditis | 46.26 | 38.36 | -17.08^*^ | 19.53 | 18.24 | -6.61^*^ | 46.35 | 39.44 | -14.91 | <0.001^#^ |
| Atrial fibrillation and flutter | 43.03 | 49.37 | 14.73^*^ | 28.16 | 33.94 | 20.53^*^ | 41.88 | 50.12 | 19.68^*^ | 0.206 |
| Aortic aneurysm | 15.14 | 11.27 | -25.56^*^ | 8.63 | 7.10 | -17.73^*^ | 14.86 | 11.79 | -20.66^*^ | 0.001^#^ |
| Chronic respiratory diseases | 1234.86 | 612.72 | -50.38^*^ | 619.02 | 281.66 | -54.50^*^ | 1206.95 | 590.97 | -51.04^*^ | 0.042^#^ |
| Chronic obstructive pulmonary disease | 977.42 | 488.52 | -50.02^*^ | 548.21 | 255.24 | -53.44^*^ | 961.68 | 484.76 | -49.59^*^ | <0.001^#^ |
| Asthma | 241.54 | 103.15 | -57.29^*^ | 65.16 | 18.05 | -72.30^*^ | 231.25 | 87.70 | -62.08^*^ | <0.001^#^ |
| Cirrhosis | 173.65 | 82.59 | -52.44^*^ | 90.30 | 49.30 | -45.40^*^ | 177.66 | 91.36 | -48.58^*^ | <0.001^#^ |
| Cirrhosis due to alcohol use | 0.42 | 0.45 | 7.14^*^ | 0.55 | 0.64 | 16.36 | 0.49 | 0.55 | 12.24 | 0.023^#^ |
| Digestive diseases | 321.23 | 219.97 | -31.52^*^ | 164.23 | 122.46 | -25.43^*^ | 300.89 | 217.60 | -27.68^*^ | <0.001^#^ |
| Peptic ulcer disease | 89.77 | 45.71 | -49.08^*^ | 40.58 | 20.67 | -49.06^*^ | 83.16 | 43.56 | -47.62^*^ | <0.001^#^ |
| Pancreatitis | 14.29 | 8.44 | -40.94^*^ | 12.77 | 8.32 | -34.85^*^ | 15.31 | 9.82 | -35.86^*^ | <0.001^#^ |
| Neurological disorders | 800.77 | 841.90 | 5.14^*^ | 726.51 | 676.37 | -6.90^*^ | 787.39 | 790.21 | 0.36^*^ | <0.001^#^ |
| Alzheimer’s disease and other dementias | 158.81 | 171.72 | 8.13^*^ | 176.12 | 162.28 | -7.86^*^ | 159.36 | 165.50 | 3.85^*^ | <0.001^#^ |
| Parkinson’s disease | 50.50 | 78.65 | 55.74^*^ | 67.39 | 99.24 | 47.26^*^ | 49.61 | 77.09 | 55.39^*^ | <0.001^#^ |
| Epilepsy | 71.43 | 94.24 | 31.93^*^ | 67.36 | 78.13 | 15.99 ^*^ | 69.35 | 88.67 | 27.86^*^ | <0.001^#^ |
| hemicrania | 376.67 | 359.25 | -4.62 | 296.44 | 237.75 | -19.80^*^ | 368.34 | 330.64 | -10.24^*^ | 0.563 |
| Mental and substance use disorders | 2507.38 | 1970.80 | -21.40^*^ | 848.99 | 781.56 | -7.94 ^*^ | 2386.09 | 2033.21 | -14.79^*^ | <0.001^#^ |
| Schizophrenia | 264.63 | 444.59 | 68.00^*^ | 89.84 | 174.50 | 94.23^*^ | 279.16 | 569.72 | 104.08^*^ | <0.001^#^ |
| Drug use disorders | 299.72 | 104.52 | -65.13^*^ | 29.14 | 11.63 | -60.09^*^ | 283.55 | 102.83 | -63.73^*^ | <0.001^#^ |
| [Depressive](file:///D:\\Users\\nfernandez\\Program%20Files%20(x86)\\Youdao\\Dict\\7.5.2.0\\resultui\\dict\\%3fkeyword=depressive)[disorder](file:///D:\\Users\\nfernandez\\Program%20Files%20(x86)\\Youdao\\Dict\\7.5.2.0\\resultui\\dict\\%3fkeyword=disorder) | 973.38 | 717.83 | -26.25^*^ | 290.41 | 210.67 | -27.46^*^ | 869.53 | 622.58 | -28.40^*^ | <0.001^#^ |
| [infantile](file:///D:\\Users\\nfernandez\\Program%20Files%20(x86)\\Youdao\\Dict\\7.5.2.0\\resultui\\dict\\%3fkeyword=infantile)[autism](file:///D:\\Users\\nfernandez\\Program%20Files%20(x86)\\Youdao\\Dict\\7.5.2.0\\resultui\\dict\\%3fkeyword=autism) | 96.22 | 69.83 | -27.43^*^ | 41.97 | 36.96 | -11.94^*^ | 91.29 | 69.33 | -24.06^*^ | <0.001^#^ |
| Diabetes, urogenital, blood, and endocrine diseases | 1892.76 | 1416.47 | -25.16^*^ | 1015.23 | 872.95 | -14.01 ^*^ | 1706.13 | 1388.51 | -18.62^*^ | <0.001^#^ |
| Diabetes mellitus | 922.97 | 493.04 | -46.58^*^ | 363.34 | 241.64 | -33.49^*^ | 830.10 | 491.04 | -40.85^*^ | <0.001^#^ |
| Chronic kidney disease | 262.06 | 237.18 | -9.49^*^ | 186.32 | 175.15 | -6.00^*^ | 232.86 | 221.14 | -5.03^*^ | <0.001^#^ |
| Gynecological diseases | 310.43 | 301.61 | -2.84^*^ | 222.52 | 209.91 | -5.67^*^ | 291.44 | 311.86 | 7.01^*^ | 0.128 |
| Hemoglobinopathies and hemolytic anemia | 280.43 | 317.65 | 13.27^*^ | 173.21 | 195.96 | 13.13^*^ | 238.43 | 298.52 | 25.20^*^ | <0.001^#^ |
| Musculoskeletal disorders | 1959.89 | 1826.06 | -6.83^*^ | 1927.06 | 1985.76 | 3.05^*^ | 1908.20 | 1996.38 | 4.62^*^ | <0.001^#^ |
| Rheumatoid arthritis | 73.58 | 77.00 | 4.65^*^ | 69.61 | 85.84 | 23.32^*^ | 73.04 | 93.36 | 27.82^*^ | <0.001^#^ |
| [Osteoarthritis](file:///D:\\Users\\nfernandez\\Program%20Files%20(x86)\\Youdao\\Dict\\7.5.2.0\\resultui\\dict\\%3fkeyword=osteoarthritis) | 178.30 | 216.56 | 21.46^*^ | 173.51 | 231.06 | 33.17^*^ | 185.73 | 244.37 | 31.57^*^ | <0.001^#^ |
| Low back and neck pain | 1153.06 | 1084.66 | -5.93^*^ | 1185.34 | 1211.30 | 2.19^*^ | 1145.30 | 1194.97 | 4.34^*^ | <0.001^#^ |
| Low back pain | 608.65 | 563.63 | -7.40 | 629.06 | 641.18 | 1.93^*^ | 606.67 | 626.36 | 3.25^*^ | <0.001^#^ |
| Neck pain | 544.40 | 521.03 | -4.29^*^ | 556.28 | 570.13 | 2.49^*^ | 538.64 | 568.60 | 5.56^*^ | <0.001^#^ |
| Other non-communicable diseases | 2140.14 | 2175.08 | 1.63^*^ | 1687.14 | 1942.15 | 15.11^*^ | 1995.47 | 2182.75 | 9.39^*^ | <0.001^#^ |
| Congenital anomalies | 580.84 | 624.22 | 7.47^*^ | 457.93 | 601.73 | 31.40^*^ | 563.39 | 668.19 | 18.60^*^ | <0.001^#^ |
| Skin and subcutaneous diseases | 612.14 | 551.02 | -9.98 | 342.96 | 320.51 | -6.55^*^ | 463.66 | 422.06 | -8.97^*^ | <0.001^#^ |
| ^#^Sense organ diseases | 766.47 | 817.83 | 6.70^*^ | 662.68 | 754.57 | 13.87^*^ | 789.21 | 896.74 | 13.63^*^ | <0.001^#^ |
| Oral disorders | 176.89 | 176.32 | -0.32^*^ | 150.83 | 164.32 | 8.94^*^ | 175.78 | 190.98 | 8.65^*^ | 0.628 |
| Injuries | 1769.86 | 998.86 | -43.56^*^ | 903.77 | 618.05 | -31.61^*^ | 1492.02 | 900.93 | -39.62^*^ | <0.001^#^ |
| Transport injuries | 612.37 | 335.69 | -45.18^*^ | 327.88 | 211.00 | -35.65^*^ | 511.21 | 317.56 | -37.88^*^ | <0.001^#^ |
| Road injuries | 577.00 | 292.66 | -49.28^*^ | 311.77 | 182.44 | -41.48^*^ | 486.32 | 285.92 | -41.21^*^ | <0.001^#^ |
| Other transport injuries | 35.37 | 43.03 | 21.66^*^ | 16.11 | 28.55 | 77.22^*^ | 24.89 | 31.65 | 27.16^*^ | <0.001^#^ |
| Unintentional injuries | 702.86 | 502.88 | -28.45^*^ | 398.47 | 324.35 | -18.60^*^ | 575.86 | 433.53 | -24.72^*^ | <0.001^#^ |
| Falls | 145.32 | 161.38 | 11.05^*^ | 109.59 | 128.63 | 17.37^*^ | 115.56 | 142.38 | 23.21^*^ | <0.001^#^ |
| Drowning | 171.27 | 97.12 | -43.29^*^ | 98.02 | 55.56 | -43.32^*^ | 140.02 | 83.99 | -40.02^*^ | <0.001^#^ |
| Fire, heat, and hot substances | 60.90 | 42.87 | -29.61^*^ | 29.15 | 26.96 | -7.51^*^ | 46.82 | 33.87 | -27.66^*^ | <0.001^#^ |
| Poisonings | 101.33 | 42.43 | -58.13^*^ | 42.88 | 18.10 | -57.79^*^ | 87.35 | 38.35 | -56.10^*^ | <0.001^#^ |
| Intentional injury | 444.42 | 155.04 | -65.11^*^ | 172.22 | 79.39 | -53.90^*^ | 396.32 | 145.05 | -63.40^*^ | <0.001^#^ |
| Self-harm and interpersonal violence | 351.73 | 119.43 | -66.04^*^ | 133.61 | 56.29 | -57.87 ^*^ | 319.33 | 114.45 | -64.16^*^ | <0.001^#^ |
| Interpersonal violence | 92.69 | 35.61 | -61.58^*^ | 38.61 | 23.10 | -40.17^*^ | 76.99 | 30.60 | -60.25^*^ | <0.001^#^ |
| Other diarrhea, lower respiratory and common infectious disease | 149.37 | 111.37 | -25.44^*^ | 86.85 | 55.15 | -36.50^*^ | 199.03 | 119.27 | -40.07^*^ | <0.001^#^ |
| Other neglected tropical disease | 161.46 | 53.71 | -66.73^*^ | 89.20 | 109.11 | 22.32^*^ | 107.66 | 66.12 | -38.58^*^ | <0.001^#^ |
| Other nutritional disease | 61.53 | 56.97 | -7.41^*^ | 57.03 | 50.70 | -11.10^*^ | 57.87 | 52.63 | -9.05^*^ | 0.991 |
| Other hepatitis | 2.70 | 1.98 | -26.67^*^ | 2.13 | 3.72 | 74.65 | 2.77 | 1.69 | -38.99^*^ | 0.723 |
| Other Communicable, maternal, neonatal, and nutritional diseases | 12.42 | 11.26 | -9.34^*^ | 11.33 | 11.25 | -0.71^*^ | 11.61 | 8.80 | -24.20^*^ | <0.001^#^ |
| Other cancer | 351.03 | 263.37 | -24.97^*^ | 300.13 | 233.05 | -22.35^*^ | 336.57 | 268.45 | -20.24^*^ | 0.613 |
| Other cardiovascular disease | 71.60 | 45.43 | -36.55^*^ | 40.36 | 28.50 | -29.39^*^ | 71.12 | 46.71 | -34.32^*^ | <0.001^#^ |
| Other chronic respiratory disease | 15.91 | 21.05 | 32.31^*^ | 5.65 | 8.37 | 48.14 ^*^ | 14.02 | 18.51 | 32.03^*^ | <0.001^#^ |
| Other Cirrhosis | 173.23 | 82.15 | -52.58^*^ | 89.75 | 48.66 | -45.78^*^ | 177.17 | 90.81 | -48.74^*^ | <0.001^#^ |
| Other digestive disease | 217.17 | 165.82 | -23.65^*^ | 110.88 | 93.47 | -15.70^*^ | 202.42 | 164.21 | -18.88 | <0.001^#^ |
| Other Neurological disorders | 143.35 | 138.03 | -3.71^*^ | 119.20 | 98.97 | -16.97^*^ | 140.73 | 128.31 | -8.83^*^ | <0.001^#^ |
| Other mental and substance use disorders | 873.43 | 634.04 | -27.41^*^ | 397.63 | 347.79 | -12.53^*^ | 862.57 | 668.75 | -22.47 ^*^ | <0.001^#^ |
| Other diabetes, urogenital, blood, and endocrine diseases | 116.88 | 66.98 | -42.69^*^ | 69.85 | 50.29 | -28.00^*^ | 113.30 | 65.95 | -41.79^*^ | <0.001^#^ |
| Other Musculoskeletal disorders | 554.94 | 447.84 | -19.30^*^ | 498.59 | 457.56 | -8.23 | 504.12 | 463.69 | -8.02^*^ | <0.001^#^ |
| Others | 3.78 | 5.69 | 50.53^*^ | 72.74 | 101.01 | 38.86^*^ | 3.44 | 4.78 | 38.95^*^ | <0.001^#^ |
| Other unintentional injury | 224.03 | 159.08 | -28.99^*^ | 118.84 | 95.10 | -19.98^*^ | 186.10 | 134.93 | -27.50^*^ | <0.001^#^ |
| Other injury | 10.21 | 5.24 | -48.68^*^ | 5.21 | 3.31 | -36.47^*^ | 8.64 | 4.78 | -44.68^*^ | <0.001^#^ |

***: Statistical significance using the test of Cochran-Armitage trend for year 2005 to 2015 comparison (P< 0.05); #: statistical significance using Poisson regression model for comparison of different cultural regions (P< 0.05).**

**Discussion**

Understanding of the regional discrepancies and similarities in disease burden may help provide not only thte interventions at individual levels, but also policy development at the governmental level. At the individual level, we need to advocate promoting health education that improves the awareness of diseases by avoiding harmful risk factors during pregnancy (such as smoking and drinking) or specific local environmental and cultural factors (such as massive consumption of pickled vegetables and processed meat, or ingestion of raw salted shellfish). Moreover, the government should improve the awareness of the substantial disease burden and its risk factors, through policy development and decision making: 1) enhancing the ability of management, diagnosis and treatment of the caregivers [1]; 2) promoting healthy diet programs at the government level (including healthy diet implementation [2]) and preempting laws on nutrition labeling contents [3] to prevent diet related disease including cardiovascular disease, or diabetes mellitus; 3) despite the launch of Healthy China 2030 campaign, we have witnessed a slow progress in reducing tobacco consumption. There was a notable burden of cardiovascular, COPD, tracheal, bronchial and lung cancer, all of which were related to smoking. Hence, the government should support and facilitate comprehensive tobacco prevention and control measures and strategies, encompassing sustained surveillance of smoking, reinforcing smoking cessation in the public place and in communities nationwide, improving the effective delivery and use of clinical and other preventive services, providing the funding to community-based organization to educate the public and mass media; 4) tackling indoor and outdoor air pollution by following the national transition to a greener economy has become a priority given that air pollution is the fourth leading risk factor at the national level for deaths and DALYs, especially for cardiovascular and respiratory diseases [4].

**Reference:**

1. Allotey P, Reidpath DD, Yasin S, Chan CK, de-Graft Aikins A. Rethinking health-care systems: a focus on chronicity. *Lancet* 2011; 377:450–51.
2. He FJ, Brinsden HC, MacGregor GA. Salt reduction in the United Kingdom: a successful experiment in public health. *J Hum Hypertens*. 2014; 28:345-52.
3. Pomeranz JL, Zellers L, Bare M, Pertschuk M. State Preemption of Food and Nutrition Policies and Litigation: Undermining Government's Role in Public Health. *Am J Prev Med*. 2019; 56:47-57.
4. Zhou M, Wang H, Zeng X, Yin P, Zhu J, Chen W, et al. Mortality, morbidity, and risk factors in China and its provinces, 1990-2017: a systematic analysis for the Global Burden of Disease Study 2017. *Lancet*. 2019. pii: S0140-6736(19)30427-1.
